# Genome Sequencing of *Xanthomonas vasicola* Pathovar *vasculorum* Reveals Variation in Plasmids and Genes Encoding Lipopolysaccharide Synthesis, Type-IV Pilus and Type-III Secretion Effectors

**DOI:** 10.3390/pathogens3010211

**Published:** 2014-03-18

**Authors:** Arthur Wasukira, Max Coulter, Noorah Al-Sowayeh, Richard Thwaites, Konrad Paszkiewicz, Jerome Kubiriba, Julian Smith, Murray Grant, David J. Studholme

**Affiliations:** 1 Biosciences, University of Exeter, Geoffrey Pope Building, Stocker Road, Exeter EX4 4QD, UK; E-Mails: awasukira@gmail.com (A.W.); mc343@exeter.ac.uk (M.C.); nama203@exeter.ac.uk (N.A.-S.); k.h.paszkiewicz@exeter.ac.uk (K.P.); m.r.grant@exeter.ac.uk (M.G.); 2 National Crops Resources Research Institute (NaCRRI), Kampala 7084, Uganda; E-Mail: jkubiriba@kari.go.ug; 3 The Food and Environment Research Agency, Sand Hutton, York YO41 1LZ, UK; E-Mails: richard.thwaites@fera.gsi.gov.uk (R.T.); julian.smith@fera.gsi.gov.uk (J.S.)

**Keywords:** gumming disease, sugarcane, banana *Xanthomonas* wilt

## Abstract

*Xanthomonas vasicola* pathovar *vasculorum* (*Xvv*) is the bacterial agent causing gumming disease in sugarcane. Here, we compare complete genome sequences for five isolates of *Xvv* originating from sugarcane and one from maize. This identified two distinct types of lipopolysaccharide synthesis gene clusters among *Xvv* isolates: one is similar to that of *Xanthomonas axonopodis* pathovar *citri* (*Xac*) and is probably the ancestral type, while the other is similar to those of the sugarcane-inhabiting species, *Xanthomonas sacchari*. Four of six *Xvv* isolates harboured sequences similar to the *Xac* plasmid, pXAC47, and showed a distinct Type-IV pilus (T4P) sequence type, whereas the T4P locus of the other two isolates resembled that of the closely related banana pathogen, *Xanthomonas campestris* pathovar *musacearum* (*Xcm*). The *Xvv* isolate from maize has lost a gene encoding a homologue of the virulence effector, *xopAF*, which was present in all five of the sugarcane isolates, while *xopL* contained a premature stop codon in four out of six isolates. These findings shed new light on evolutionary events since the divergence of *Xvv* and *Xcm*, as well as further elucidating the relationships between the two closely related pathogens.

## 1. Introduction

The bacterial genus, *Xanthomonas*, includes many economically important pathogens of crop plants [1]. Genome sequencing of species and pathovars of *Xanthomonas* has led to insights into the evolution and mechanisms of virulence and their ability to overcome host defences [2]. One pathovar whose study at the molecular level has been relatively limited is *Xanthomonas vasicola* pathovar *vasculorum* (*Xvv*).

Strains of *Xvv* are responsible for gumming disease in sugarcane and are also found naturally infecting some other monocotyledonous hosts, including maize and sorghum [3]. In addition to the importance of gumming disease and *Xvv* *per se*, strains of this pathovar share a recent common ancestor with the causal agent of banana *Xanthomonas* wilt, namely *Xanthomonas campestris* pathovar *musacearum* (*Xcm*), with which they share an identical *gyrB* DNA sequence [4]. Strains of *Xvv* do not cause disease in banana [4], but *Xcm* can infect maize and sugarcane.

The narrow genetic diversity in *Xcm* [5,6] suggests a population bottleneck, and *Xcm* may represent a single clonal lineage of the species, *X. vasicola*, or a closely related species, that has recently emerged to colonise bananas in east Africa. Whole-genome sequence data can provide greater phylogenetic resolution than analysis of a single gene fragment. Although all isolates shared identical *gyrB* amplicon sequences, analyses of genome-wide sequence data revealed that *Xcm* and *Xvv* comprise two distinct clades, although they are closely related to each other [7]. This suggests that *Xcm* did not arise from *Xvv*, but rather from some currently unknown close relative of *Xvv*, possibly another pathovar belonging to the same species (*X.* *vasicola*).

Given the close phylogenetic relationship between *Xcm* and *Xvv*, molecular comparison between *Xcm* and *Xvv* is of importance for understanding the evolution of *Xcm* and the adaptation to banana. To better understand the range of diversity within *Xvv*, we analysed available genome sequence data for four isolates of *Xvv* (described in previous publications [7,8]) and new sequence data from a further two isolates of *Xvv*.

There has been some confusion in the literature regarding the taxonomy of the *Xvv* strains, whose genome sequences we analyse here. Lewis Ivey and colleagues [9] listed strains NCPPB (National Collection of Plant Pathogenic Bacteria) 702, NCPPB 1326 and NCPPB 206 as *X. axonopodis* pathovar *vasculorum.* In contrast, Qhobela and Claflin [10] classified strain NCPPB 1326 as *X. campestris* pathovar *vasculorum*. However, Vauterin and colleagues [11] proposed that these strains be included in the species, *X. vasicola* (as pathovar *vasculorum*), on the basis of genomic DNA hybridization studies. A subsequent analysis of cellular fatty acid profiles also confirmed a close affinity between these strains and strains of the species, *X. vasicola*, and only rather distant similarity to species *X. axonopodis* and *X. campestris* [3]. Rademaker and colleagues [12] also list strain NCPPB 206 (synonymous with LMG 8284) as belonging to species *X. vasicola* (pathovar *vasculorum*, *i.e.*, *Xvv*). Overall, the available evidence overwhelmingly supports the inclusion of these isolates in species *X. vasicola*, and therefore, in this manuscript, we refer to them as *Xvv* (rather than as *X. campestris* or *X. axonopodis*).

We previously generated draft genome sequences for one isolate of *Xcm* and one isolate of *Xvv* and identified several differences between them that might contribute to their distinct host ranges [8]; we found differences between *Xcm* NCPPB 4381 and *Xvv* NCPPB 702 in their repertoires of Type-III secretion system (T3SS) effectors, lipopolysaccharide (LPS) synthesis genes and Type-IV pilus (T4P) genes, but these differences were between just a pair of isolates, and so, it was unclear how generalisable these results were to other isolates of *Xcm* and *Xvv*. In a more recent publication, we reported sequencing the genomes of a further 13 isolates of *Xcm* and three isolates of *Xvv* [7]. The main focus of that study [7] was genetic variation among isolates of *Xcm*, revealing two main phylogenetic groups (or sub-lineages) among *Xcm*. That previous study [7] reported a list of genes that were consistently conserved in *Xcm* and absent from the four *Xvv* genome sequences then available, and the study used data from the four *Xvv* genome sequences to generate a phylogenetic tree; however, no further analysis of the *Xvv* genome sequences was reported.

The current study extends the previous work by systematically searching for genetic differences among *Xvv* isolates, rather than focusing on variation between *Xvv* and *Xcm* or variation among isolates of *Xcm*. Furthermore, included in the current study are sequence data from two additional strains of *Xvv*, bringing the total number of sequenced *Xvv* isolates up to six. The sequence analyses presented here revealed large differences in gene-content among isolates of *Xvv*, both among isolates from sugarcane and also between isolates from sugarcane and a single isolate from maize. Some of these differences in gene content are ascribed to the gain and loss of plasmids, though many of the differences are likely to be chromosomally located.

We also report genetic differences implicated in important extracellular structures, such as lipopolysaccharide (LPS) synthesis, the T4P and candidate substrates (*i.e*., effectors) of the T3SS. Furthermore, further analysis of previously published data revealed some hitherto undetected differences in gene content among *Xcm* isolates, including a homologue of T3SS effector XopL and a plasmid.

## 2. Results and Discussion

**Figure 1:**
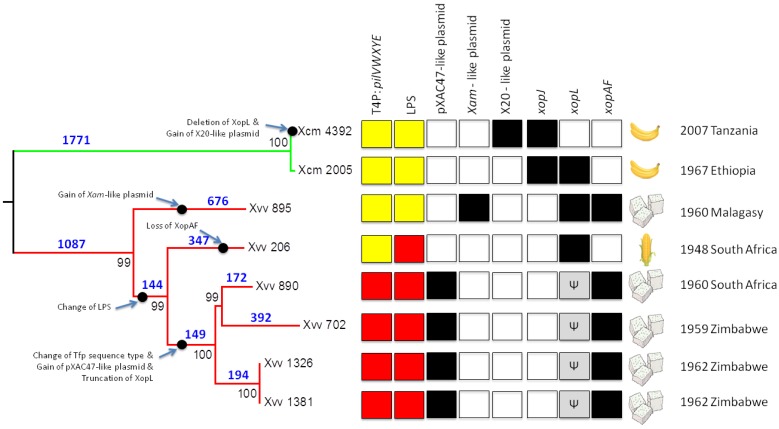
Overview of some key genetic changes during the evolution of *Xvv* and *Xcm*. The phylogenetic tree on the left-hand side (left) was inferred using the maximum parsimony method based on 39,665 single-nucleotide variants with respect to the chromosome of *Xoo* MAFF 311018. Bootstrap values are expressed as percentages of 500 trials in black. The tree is drawn to scale with branch lengths calculated using the average pathway method. For clarity, the numbers of single-nucleotide differences are indicated on the branches in blue boldface. The square boxes indicate presence (black) or absence (white). The grey boxes marked “Ψ” denote that the *xopL* gene is interrupted by a premature stop codon. The two distinct types of the T4P *pilVWXYE* gene cluster (see Section 2.7 and Figure 4) are indicated, respectively, by yellow and red shading. The two different sequence types of the lipopolysaccharide (LPS) gene cluster (see Section 2.8 and Figure 6) are indicated, respectively, by yellow and red shading. The illustrations on the right-hand side (right) indicate the origin of each bacterial isolate: banana/enset, sugarcane or maize.

**Figure 2:**
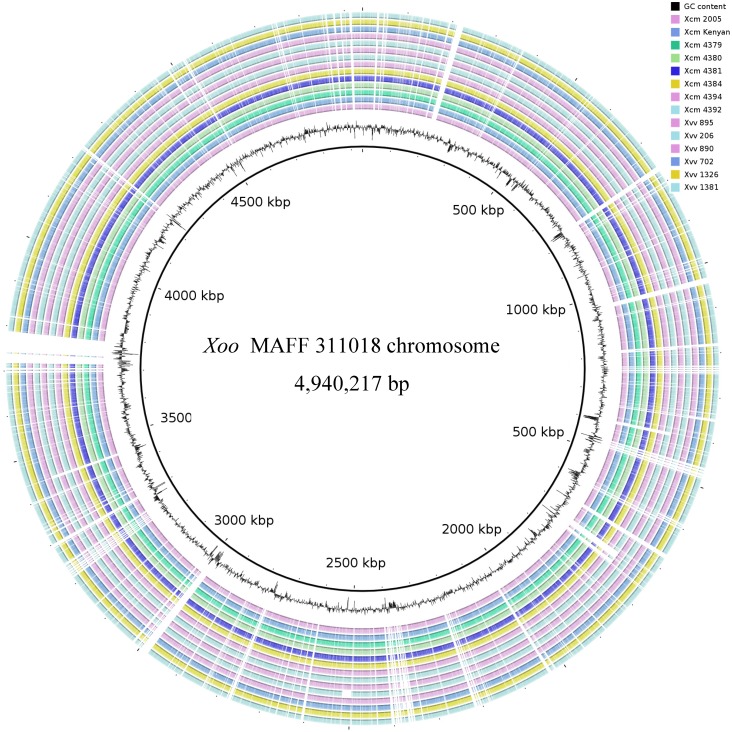
Global comparison of *Xvv* and *Xcm* genomes with reference to the *Xoo* chromosome sequence. We aligned each of the genome sequence assemblies (the GenBank accession numbers are listed in Table 1) against the *Xoo* MAFF 311018 chromosome sequence using Basic Local Alignment Search Tool Nucleotide tool BLASTN. The innermost ring indicates the genomic position. The next ring is a plot of G + C content. The remaining 14 concentric rings indicate the presence or absence of BLASTN hits at that position, with one ring corresponding to each of 14 *Xcm* and *Xvv* genome assemblies. To aid clarity, each ring is represented in a different colour. Positions covered by BLASTN alignments are indicated with a solid colour; whitespace gaps represent genomic regions not covered by the BLASTN alignments. The accession numbers of the genome assemblies used in this figure are listed in Table 3. The graphical view of the alignments was rendered using BLAST Ring Image Generator (BRIG).

**Figure 3:**
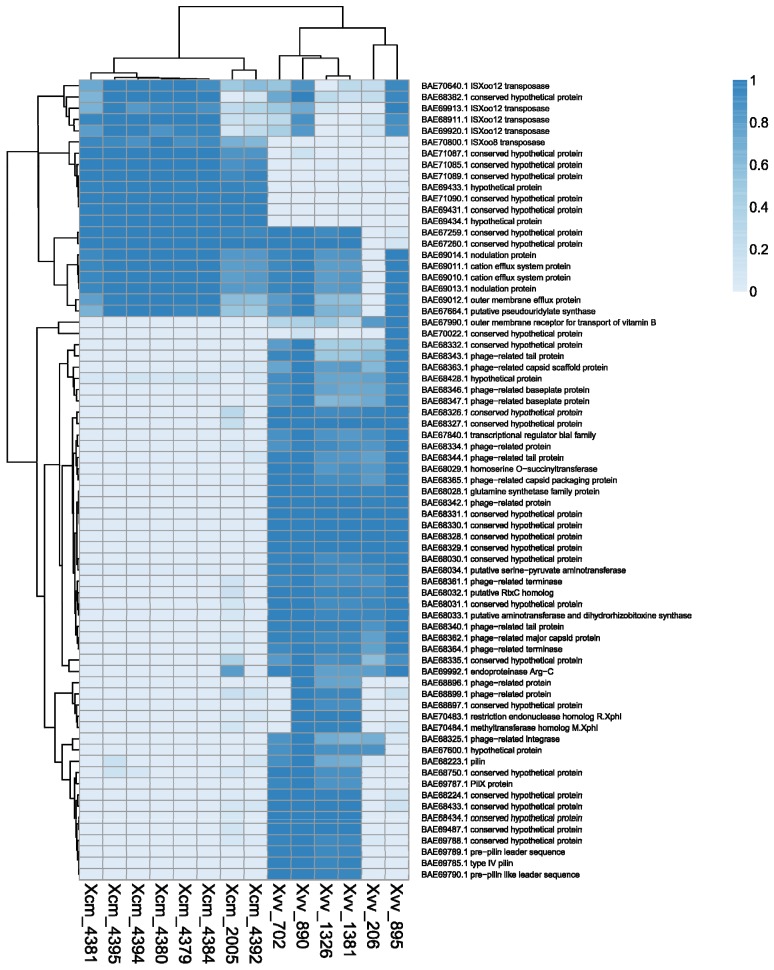
Heatmap depicting *Xvv* and *Xcm* gene-contents with reference to the *Xoo* chromosome sequence. We aligned each of the sets of genomic DNA sequence reads (SRA accession numbers listed in Table 2) against the *Xoo* MAFF 311018 chromosome sequence using BWA. We used the coverageBed tool from BEDtools to calculate the breadth of coverage of each *Xoo* gene by genomic sequence data from each *Xvv* and *Xcm* isolate.

**Figure 4:**
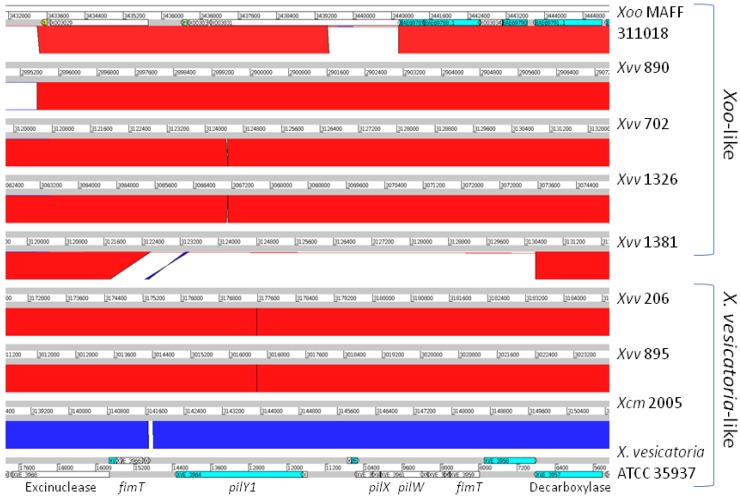
Two sequence types of the *pilVWXYE* gene cluster in *Xvv*: *Xoo*-like and *X. vesicatoria*-like. BLASTN alignments between the genome assemblies are displayed using the Artemis Comparison Tool (ACT). In the chromosome of *Xoo* MAFF 311018, this cluster falls between Positions 3436570 and 3443633 and includes locus tags XOO3030 to XOO3035. It includes the accessions BAE6788, BAE6789, BAE69785, BAE69787 and BAE69790 that appear in the heatmap in Figure 3; accession numbers: AP008229.1 (*Xoo* MAFF 311018) and AEQV01000175.1 (*X. vesicatoria* ATCC 35937). Each genome is represented by a horizontal white bar, labelled with the genomic position, flanked above and below by grey bars. Between each adjacent pair of genomes, BLASTN alignments are indicated by red blocks (for alignments between the positive strands of both sequences) or blue blocks (for alignments between the opposite strands of both sequences). Note, however, that the orientation of the BLASTN alignment (and hence, red or blue colouring) is not biologically significant, but rather just reflects the direction in which the double-stranded genome sequence has been represented in the database. Positions of annotated genes are indicated on the genome sequence of *X. vesicatoria* ATCC 35937. Note that *Xvv* 890, *Xvv* 702, *Xvv* 1326 and *Xvv* 1381 share sequence similarity along the whole length of this genomic region, and for most of the region, they also share sequence similarity with *Xoo* MAFF 311018. They differ, over the central part of this genomic region, from *Xvv* 206, *Xvv* 895 and *Xcm* 2005, all of which show sequence similarity to *X. vesicatoria* ATCC 35937 over this region. See Section 2.7.1.

**Figure 5:**
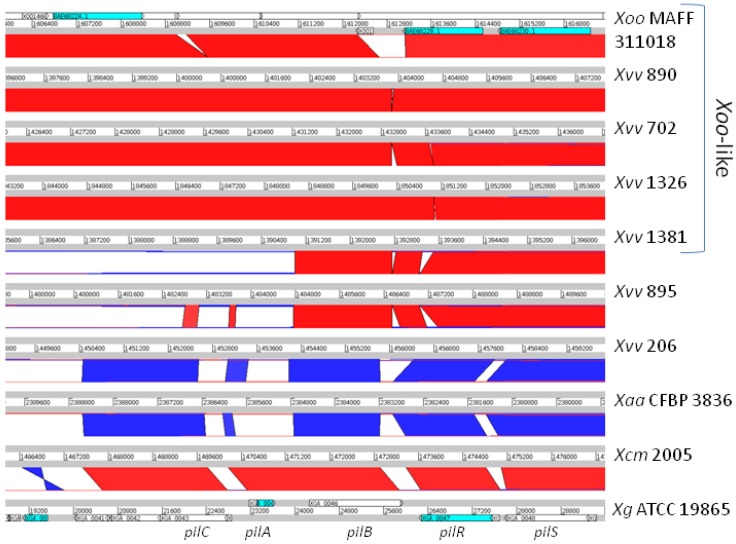
Multiple sequence types of the *pilCABRS* gene cluster in *Xvv*. BLASTN alignments between the genome assemblies are displayed using the Artemis Comparison Tool (ACT). In *Xoo* MAFF 311018, this cluster is located between Positions 1613866 and 1616597 and encompasses Loci XOO1467 to XOO1475; accession numbers: AP008229.1 (*Xoo* MAFF 311018); AUWN01000008.1 (*X.* *alfalfae* subsp. *alfalfae* (*Xaa*) CFBP 3836); and AEQX01000002.1 (*X. gardneri* (*Xg*) ATCC 19865). Each genome is represented by a horizontal white bar, labelled with the genomic position, flanked above and below by grey bars. Between each adjacent pair of genomes, BLASTN alignments are indicated by red blocks (for alignments between the positive strands of both sequences) or blue blocks (for alignments between the opposite strands of both sequences). Note, however, that the orientation of the BLASTN alignment (and hence, blue or red colouring) is not biologically significant, but rather just reflects the direction in which the double-stranded genome sequence has been represented in the database. Positions of annotated genes are indicated on the genome sequence of *X. vesicatoria* ATCC 35937. Note that *Xvv* 890, *Xvv* 702, *Xvv* 1326 and *Xvv* 1381 share sequence similarity along the whole length of this genomic region, and for most of the region, they also share sequence similarity with *Xoo* MAFF 311018. However, the *pilC* and *pilA* genes are highly divergent among *Xvv* 895, *Xvv* 206 and *Xcm* 2005, with *Xcm* 2005 having *X. gardneri*-like *pilC* and *pilA* genes. *Xvv* 895 and *Xvv* 206 have *Xaa*-like *pilC* and *pilA* genes. See Section 2.7.2.

**Figure 6:**
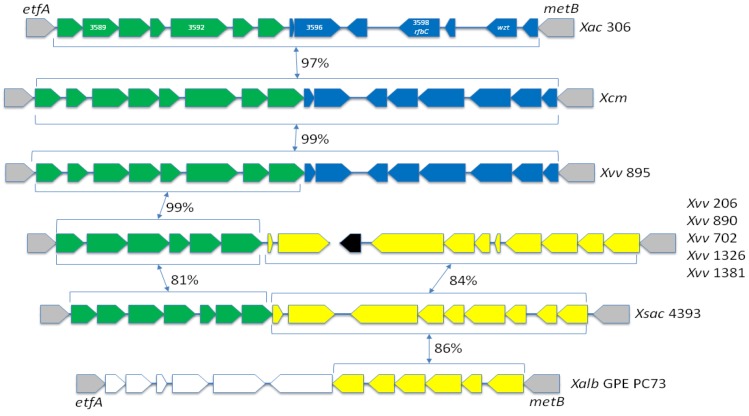
LPS biosynthesis gene clusters found in sequenced genomes of *Xvv* and related LPS clusters from other xanthomonads. The arrows represent predicted genes and are coloured according to blocks of sequence similarity. In all xanthomonads sequenced to date, the LPS cluster lies between the highly conserved *etfA* and *metB* genes; these are indicated by grey arrows. The black arrow indicates a gene encoding a mobile element protein that is not conserved in *Xvv* NCPPB 895 nor in *Xsac* NCPPB 4393. Pairwise nucleotide sequence identities between blocks of genes are given as percentages. Sequences from each of the *Xcm* sequences share at least 99% identity with *Xcm* NCPPB 2005 over the full length of the LPS cluster. The sequences from *Xvv* NCPPB 206, 890, 1326 and 1381 all share at least 99% identity with that of *Xvv* NCPPB 702. Note that the LPS cluster falls into two distinct scaffolds in the genome assembly for *Xvv* NCPPB 702. Accession numbers: NC_003919.1 (Xac 306); AKBE01000022.1 (*Xcm* NCPPB 2005); AKBO01000066.1 (*Xvv* NCPPB 895); ACHS01000119.1 and ACHS01000380.1 (*Xvv* NCPPB 702); AGDB01000011.1 (*Xsac* NCPPB 4393); FP565176.1 (*Xalb* GPE PC73); AKBM01000022.1 and AKBM01000058.1 (*Xvv* 206); AKBN01000985.1, AKBN01000620.1 and AKBN01000538.1 (*Xvv* 890); AKBK01000238.1 and AKBK01000490.1 (*Xvv* 1381); and AKBL01000107.1 and AKBL01000264.1 (*Xvv* 1826).

By comparison of genome sequence data, we identified several likely important events in the evolutionary history of *Xvv* and *Xcm*; these are summarized in Figure 1 and include the acquisitions of plasmids and the exchange of genes encoding LPS biosynthesis and T4P, as well as the loss and gain of candidate T3SS effector genes. The evidence supporting the proposal of each of these events is presented in Subsections 2.2–2.9 and Figure 2, Figure 3, Figure 4, Figure 5 and Figure 6. Further details of these findings are available in the Supplementary Material, which contains an additional 19 figures. The main findings were that: a plasmid similar to pXAC47 is found in a clade of four *Xvv* isolates from sugarcane (Section 2.4); the most ancestrally branching *Xvv* sugarcane isolate (NCPPB 895) may contain a plasmid similar to that from a cassava pathogen (Section 2.5); some (but not all) *Xcm* isolates harbour sequences similar to that from a plasmid in a cotton pathogen (Section 2.6); there is considerable variation in T4P genes among *Xvv* isolates (Section 2.7); there are two distinct sequence types of the LPS biosynthesis gene cluster among *Xvv* isolates (Section 2.8); and *Xvv* isolates vary with respect to their repertoires of putative T3SS effector genes (Section 2.9).

### 2.1. Overview of Sequence Data

Table 1 gives a brief description of the bacterial isolates from which the sequencing data used in this study originate. Table 2 lists summary statistics for the raw sequence data and Table 2 summarises the *de novo* assembly statistics. The sequence data from *Xcm* and for one *Xvv* isolate (*Xvv* 702) were described in previous publications [7,8]. The sequence data for four of the *Xvv* isolates were mentioned in a previous publication [7], but assembly statistics were not given. Therefore, we provide details of the assemblies here (Table 3). The contiguity of the assemblies for the two newly presented isolates (*Xvv* 890 and *Xvv* 895) are much lower than those of the previously presented assemblies (see N_50_ in Table 3). However, it should be noted that the incompleteness of *de novo* assemblies does not invalidate the results presented in the current study, since our inferences are based upon comparisons of alignments of raw reads rather than comparisons between assemblies; these alignments consist of unassembled read-pairs aligned against various reference genome sequences, and several examples of such alignments are illustrated in the Supplementary Material. The single exception is Figure 2, in which the comparison consists of alignments between *de novo* assemblies; it is possible that some gaps in the alignments in Figure 2 could arise through the incompleteness of the *de novo* assemblies. Figure 4 and Figure 5 are based on alignments between *de novo* assemblies, but the findings were also validated by the inspection of alignments of raw (unassembled) sequence reads (see Supplementary Material).

Five out of the six *Xvv* strains were originally isolated from sugarcane; the exception is 206, which was isolated from maize. Genomes of *Xvv* 890 and 895 were newly sequenced for this study. Genome sequences of *Xvv* 206, 1326 and 1381 were reported in a previous publication [7], but with only very limited analysis, since the focus of that study was on single-nucleotide polymorphism (SNP) in *Xcm*. The genome sequence of *Xvv* 702 was previously reported and compared against that of *Xcm* 4381 [8]. We also included genome sequence data from several isolates of *Xcm* that have previously been published [7,8], because these are the sequenced genomes most closely related to *Xvv* and probably belong to the same species, *X. vasicola* [4].

**Table 1:** Bacterial isolates and sequence datasets used in this study.

Isolate (NCPPB number)	Source and date of isolation	Source
*Xvv* 206 ^a^	South Africa 1948	*Zea mays*
*Xvv* 702 ^b^	Zimbabwe 1959	*Saccharum officinarum*
*Xvv* 890 ^c^	South Africa 1960	*S. officinarum*
*Xvv* 895 ^c^	Malagasy Republic 1960	*S. officinarum*
*Xvv* 1326 ^a^	Zimbabwe 1962	*S. officinarum*
*Xvv* 1381 ^a^	Zimbabwe 1962	*S. officinarum*
*Xcm* 2005 ^a^	Ethiopia 1967	*Ensete ventricosum*
*Xcm* 2251 ^a^	Ethiopia 1969	*Musa* sp.
*Xcm* 4379 ^a^	Uganda 2007	*Musa* sp.
*Xcm* 4380 ^a^	Uganda 2007	*Musa* sp.
*Xcm* 4381 ^b^	Uganda 2007	*Musa* sp.
*Xcm* 4383 ^a^	Uganda 2007	*Musa* sp.
*Xcm* 4384 ^a^	Uganda 2007	*Musa* sp.
*Xcm* 4387 ^a^	D. R. Congo 2007	*Musa* sp.
*Xcm* 4389 ^a^	Rwanda 2007	*Musa* sp.
*Xcm* 4392 ^a^	Tanzania 2007	*Musa* sp.
*Xcm* 4394 ^a^	Tanzania 2007	*Musa* sp.
*Xcm* 4395 ^a^	Tanzania 2007	*Musa* sp.
*Xcm* “Kenyan” ^d^	Kenya (year not known)	*Musa* sp.

^a^ These sequence data were previously reported in [7]. ^b^ These sequence data were previously reported in [8]. ^c^ These sequences were newly generated for this study. ^d^ This sequence assembly was submitted to GenBank by the International Institute of Tropical Agriculture in 2011, but no accompanying manuscript has been published to the best of our knowledge. NCPPB, National Collection of Plant Pathogenic Bacteria.

**Table 2:** Summary of sequence data.

Isolate (NCPPB number)	Number of read pairs	Read length	Coverage	SRA accession
*Xvv* 206 ^a^	2,579,404	76	70 x	SRR494500.3
*Xvv* 702 ^b^	2,913,785	36	35 x	SRR020202.3
*Xvv* 890 ^c^	4,843,028	67	58 x	SRR1045340
*Xvv* 895 ^c^	2,867,513	67	50 x	SRR1045341
*Xvv* 1326 ^a^	2,365,912	76	63 x	SRR494491.5
*Xvv* 1381 ^a^	2,450,234	76	66 x	SRR494499.3
*Xcm* 2005 ^a^	2,536,030	76	72 x	SRR489154.7
*Xcm* 4379 ^a^	3,652,875	76	102 x	SRR494484.2
*Xcm* 4380 ^a^	4,069,509	76	113 x	SRR494485.2
*Xcm* 4381 ^b^	5,052,905	36	56 x	SRR020203.3
*Xcm* 4384 ^a^	1,976,797	76	55 x	SRR494488.2
*Xcm* 4392 ^a^	2,554,161	76	72 x	SRR494498.3
*Xcm* 4394 ^a^	3,295,956	76	92 x	SRR494489.1
*Xcm* 4395 ^a^	4,117,662	76	117 x	SRR494490.2

^a^ These sequence data were previously reported in [7]. ^b^ These sequence data were previously reported in [8]. ^c^ These sequences were newly generated for this study.

**Table 3:** Summary statistics for *de novo* genome sequence assemblies.

Isolate (NCPPB number)	Number of scaffolds	Scaffolds N_50_	Total length (b.p.)	GenBank accession
*Xvv* 206 ^a^	177	103,376	4,825,935	AKBM00000000.1
*Xvv* 702 ^b^	97	205,000	5,478,002	ACHS00000000.1
*Xvv* 890 ^c^	2,565	3,627	4,951,053	AKBN00000000.1
*Xvv* 895 ^c^	1,229	8,362	4,803,807	AKBO00000000.1
*Xvv* 1326 ^a^	253	74,455	4,951,570	AKBK00000000.1
*Xvv* 1381 ^a^	137	108,088	4,958,101	AKBL00000000.1
*Xcm* 2005 ^a^	156	61,233	4,692,764	AKBE00000000.1
*Xcm* 4379 ^a^	95	147,554	4,758,198	AKBF00000000.1
*Xcm* 4380 ^a^	87	149,435	4,751,644	AKBG00000000.1
*Xcm* 4381 ^b^	115	143,688	4,793,900	ACHT00000000.1
*Xcm* 4384 ^a^	84	157,780	4,741,777	AKBH00000000.1
*Xcm* 4392 ^a^	162	90,974	4,728,564	AKBI01000000.1
*Xcm* 4394 ^a^	85	151,917	4,792,617	AKBJ00000000.1
*Xcm* “Kenyan” ^d^	510	36,401	4,907,936	AGFQ01000000.1

^a^ These sequence data were previously reported in [7]. ^b^ These sequence data were previously reported in [8]. ^c^ These sequences were newly generated for this study. ^d^ This sequence assembly was submitted to GenBank by the International Institute of Tropical Agriculture in 2011, but no accompanying manuscript has been published to the best of our knowledge.

### 2.2. Phylogenetic Relationships among Xvv Strains

We generated a phylogenetic reconstruction of the sequenced *Xvv* and *Xcm* strains based on single-nucleotide polymorphisms called against the reference sequence of the *X. oryzae* pathovar *oryzae* (*Xoo*) MAFF 311018 [13]. The maximum parsimony phylogenetic tree is shown in Figure 1; the maximum likelihood method produced identical topology, and the topology is consistent with the tree that we previously presented [7]. The *Xvv* strains form a monophyletic clade closely related to, but distinct from, *Xcm* and the genetic distances among these sequenced *Xvv* isolates are considerably larger than those among *Xcm* isolates.

Within the sequenced *Xvv* isolates, the single isolate from maize falls within the diversity of sugarcane isolates; in other words, there are not separate monophyletic groups for isolates from the two different hosts. Two of the sugarcane-derived isolates (*Xvv* 1326 and 1381) are indistinguishable on the basis of the SNPs used to generate the tree in Figure 1. They had both been collected from sugarcane in Zimbabwe in 1962 and may be essentially two isolates of the same bacterial population. However, these two isolates are genetically distinct from the isolate collected from sugarcane in Zimbabwe three years earlier (*i.e*., *Xvv* 702).

### 2.3. Global Genomic Comparison of Genomes of Xvv and Xcm Isolates

Alignment of our genome assemblies against a closely related reference chromosome sequence (*Xoo* MAFF 311018) suggested numerous differences in gene content both between *Xvv* and *Xcm* and also among *Xvv* isolates (see Figure 2). Particularly noticeable in Figure 2 is a region of the *Xoo* genome (centred at position 2,508 Mb) that is absent from *Xvv* 206, the isolate from maize. This consists of an 18-kb region of the genome, including the eight loci XOO2253–XOO2263 (GenBank accession numbers: BAE69008.1–BAE69018.1) that includes several predicted efflux proteins of unknown function. The absence of this region in *Xvv* 206 was confirmed by the alignment of sequence reads against the *Xoo* reference genome, independently of any *de novo* assembly.

We identified *Xoo* genes that are differentially present or missing in each of the *Xvv* and *Xcm* isolates (Figure 3). To do this, we aligned the Illumina sequence reads against the *Xoo* chromosome sequence using BWA [14]. We then calculated the breadth of coverage of each *Xoo* gene in each *Xvv* and *Xcm* isolate using the coverageBed tools from the BEDtools suite [15]. Note that this analysis involved the alignment of raw sequence reads against the reference; it was not dependent on any *de novo* assembly of our sequence data. We confirmed differential presence/absence by PCR amplification from genomic DNA (Figure 4). Breadths of coverage for each gene are indicated by the heatmap in Figure 3, which reveals numerous genes, whose presence distinguishes *Xcm* from *Xvv* and also several that distinguish among *Xvv* isolates. However, this approach is limited to the analysis of genes that are in the chromosome of *Xoo*. It excludes plasmids, as well as chromosomal genes in *Xvv* or *Xcm* that are not conserved in *Xoo*. Therefore, we also performed similar analyses based on using genomic assemblies of *Xvv* and *Xcm* genomes instead of the *Xoo* chromosome sequence. The results of these analyses are presented as heatmaps in the Supplementary Material. Some of these differences in gene content are discussed in more detail below.

### 2.4. Xvv Isolates NCPPB 890, 702, 1326 and 1381 Contain Sequences Similar to Plasmid pXAC47

We searched for evidence of plasmids in the *Xvv* and *Xcm* genomes by aligning the Illumina reads against all bacterial plasmid sequences in the RefSeq database as of the October 31, 2013. The *Xvv* Isolates 890, 702, 1326 and 1381 all yielded Illumina sequence reads that cover about 70% of the length of plasmid pXAC47 from *Xanthomonas* *axonopodis* pathovar *citri* (*Xac*) 29-1 (the RefSeq accession number is NC_020798.1). The genomes of *Xvv* Isolates 890, 702, 1326 and 1381 all contain sequences with extensive similarity to pXAC47, while the other isolates of *Xvv* (and *Xcm*) do not.

### 2.5. Xvv Isolate NCPPB 895 Contains Sequences Similar to a Plasmid from X. axonopodis pv. manihotis (Xam)

We found extensive sequence similarity between *Xvv* 895 and sequences from several recently published draft genome sequences [16] of *Xam*, a pathogen of cassava. These sequences are not found in the other sequenced *Xvv* isolates nor in the sequenced *Xcm* isolates. Specifically, a 34.7-kb *Xvv* 890 contig (GenBank: AKBO01000002.1) shared 99% nucleotide sequence identity with contigs from *Xam* strains NG1, UA306, ORST17 and ORST X27 originally isolated from Nigeria, Columbia, Congo and Togo (the GenBank accession numbers for these *Xam* sequences are, respectively: AKEG01000263.1, AKEN01000114.1, AKEH01000160.1 and AKEJ01000091.1).

Some other strains of *Xam* also contained some slightly less-similar sequences, sharing up to 89% nucleotide sequence identity, including strains IBSBF725, IBSBF436, IBSBF285, IBSBF2820 and UA324. Most of the 41.4-kb sequence from *Xam* ORST X27 is conserved in *Xvv* 895, but absent from the other sequenced *Xvv* and *Xcm* isolates. This sequence is annotated as a (partial) plasmid [16], presumably because it contains several conjugative transfer genes. Overall, these observations indicate that *Xvv* 895 has acquired a plasmid that is closely related to plasmids that are circulating in *Xam* strains, suggesting the inter-species exchange of plasmids over a wide geographic range.

### 2.6. Xcm Isolates NCPPB 4379, 4380, 4383, 4384, 4392 and 4395 Contain Sequences Similar to a Plasmid from *X. citri* pv. *malvacearum* Strain X20

Previously, we found no evidence for the presence of plasmids in *Xcm* 4381 [8]. However, examination of data from *Xcm* Isolates 4379, 4380, 4383, 4384, 4392 and 4395 revealed extensive sequence similarity to a recently reported plasmid sequence from *X. citri* pv. *malvacearum* strain X20, a highly virulent pathogen of cotton isolated in Burkina Faso [17]. This 39-kb plasmid sequence (GenBank: CM002030.1) was not present in any of the sequenced *Xvv* isolates nor in any of the *Xcm* isolates belonging to Sub-lineage I (as defined by [7]); it appears to be restricted to *Xcm* Sub-lineage II among the isolates of which it is present in all, except *Xcm* 4381. The most parsimonious explanation for this pattern of distribution is that this plasmid was acquired by the common ancestor of *Xcm* Sub-lineage II and subsequently lost in *Xcm* 4381. The GenBank accession number for the corresponding plasmid sequence in the *Xcm* 4384 *de novo* assembly is AKBH01000036.1. The average nucleotide sequence identity between the *X. citri* pv. *Malvacearum* plasmid and *Xcm* was 92%, somewhat lower than the 99% identity between *Xvv* 895 and *Xam* plasmid sequences.

### 2.7. Genetic Variation in the Type-IV Pilus (T4P) Among Isolates of Xvv

The global analyses of gene content (see Figure 3) revealed differences among the *Xvv* isolates with respect to their T4P genes (Figure 4 and Figure 5). For example, Figure 3 shows that *Xvv* Isolates 702, 890, 1326 and 1381 are distinguished from the other sequenced isolates by the presence of several T4P-related genes (including GenBank accessions: BAE68223, BAE6788, BAE6789, BAE69785, BAE69787 and BAE69790). 

#### 2.7.1. The *pilVWXYE* Gene Cluster

In *Xanthomonas* species, the T4P apparatus is encoded by clusters of genes scattered over several genomic locations, including two large clusters containing *pilVWXYE* and *pilCABRS*, respectively. The first of these two clusters falls between a gene encoding an excinuclease ABC subunit B and a gene encoding a decarboxylase-family protein. We found two distinct sequence types at this locus among *Xvv* isolates. The corresponding gene cluster in *Xvv* 702 encodes homologues of FimT, PilV, PilW, PilX, PilY and PilE and is highly conserved (at least 99% identical nucleotide sequence) in *Xvv* Isolates 890, 1326 and 1381 and *Xoo* (92% identity). However, the corresponding gene cluster is quite different in the other two sequenced *Xvv* isolates, namely 206 and 895. These two isolates share 99% nucleotide sequence identity with *Xcm* NCPPB 4381 and the other sequenced isolates of *Xcm* and 90% identity with *X. vesicatoria* ATCC 35937 [18].

In summary, there are two distinct sequence types at this *pilVWXYE* locus among *Xvv* isolates: (i) *Xoo*-like (in *Xvv* 890, 702, 1326 and 1381); and (ii) *X. vesicatoria*-like (*Xvv* 895, 206 and all *Xcm*); see Figure 4. The most parsimonious explanation for this pattern is horizontal transfer of the locus in a common ancestor of *Xvv* Isolates 890, 702, 1326 and 1381 after splitting from the *Xvv* 206 lineage (see Figure 1). A less parsimonious explanation would be multiple acquisitions of the same sequence.

#### 2.7.2. The *pilCABRS* Gene Cluster

In addition to the *pilVWXYE* gene cluster described above, there is also variation at the *pilCABRS* gene cluster, with *pilA* (locus tag XOO1468 and accession number BAE68223 in *Xoo*) being particularly variable (Figure 5). The pattern of variation at this locus is similar to that at *pilVWXYE*, insofar as *Xvv* Isolates 890, 702, 1326 and 1381 have a *Xoo*-like sequence at this locus (94% nucleotide sequence identity between *Xvv* and *Xoo*), whereas *Xvv* Isolates 206 and 895 have a different sequence type.

The *pilA* genes are of different sequence types in *Xvv* 206, *Xvv* 895 and *Xcm*. The nucleotide sequence of *Xvv* 206 *pilC* and *pilA* is 87% identical to those of *Xcm*. This degree of sequence identity is significantly lower than for the core genome; most orthologous genes share at least 99% nucleotide sequence identity between *Xvv* and *Xcm*. Apart from *Xcm*, the next most similar sequence to *Xvv* 206 *pilC* and *pilA* comes from *X.* *alfalfae* subsp. *alfalfae* (*Xaa*) CFBP 3836 (79% identity). In *Xvv* 895, *pilB* is 95% identical to *Xvv* 206 and 94% identical to *Xcm*. However, at the *pilA* locus, there is further variation between *Xvv* 206, *Xvv* 895 and *Xcm*. The most similar sequence (as of November, 2013) in the public databases to the *pilA* gene of *Xvv* 895 is *X.* *alfalfae* subsp. *alfalfae* CFBP 3836 (90% identity). The *pilA* of *Xcm* shares 94% nucleotide sequence identity with *X. gardneri* ATCC 19865 [18] and shows no detectable nucleotide sequence similarity with any other sequence in the public databases.

The pattern of sequence variation in the *pilCABRS* gene cluster indicates multiple superimposed horizontal transfer events, resulting in *Xvv* having three distinct sequence types of *pilA*: (i) the *Xoo*-type in *Xvv* 702, 890, 1326 and 1381; (ii) the *Xvv* 895 type that is 94% identical to *Xaa*; and (iii) the *Xvv* 206 type that is 79% identical to *Xaa*. The *pilA* of *Xcm* belongs to a fourth, *X. gardneri*-like type.

#### 2.7.3. Concluding Remarks about Variation in T4P Genes

The T4P is a key virulence factor for phytopathogenic bacteria [19]. It performs a range of functions, including twitching motility [20,21,22,23,24,25,26] and cell-to-cell adhesion [27,28], thereby playing a role in the formation of micro-colonies and biofilms [29,30,31,32], and PilA has been implicated in the transmission of the pathogen to seed [31]. It is clear from our results that there have been several horizontal genetic transfers resulting in the replacement of T4P genes with alternative alleles in *X. vasicola*. It is not clear what functional significance, if any, arises from such allele exchanges, and there is no clear-cut correlation between T4P sequence type and host plant species. However, given the key role of the T4P in bacteria—plant interactions and the previously reported observation that some T4P genes are under selection in *Xanthomonas* species [33]—this may warrant further investigation. It is also possible that a phage might exert a selective pressure on T4P; for example, some phages require the T4P to infect *Pseudomonas aeruginosa* [34].

### 2.8. There Are Two Distinct Sequence Types of LPS Biosynthesis Clusters in *Xvv*

The lipopolysaccharide (LPS) molecules that cover the outer membranes of Gram-negative bacteria [35,36] can be an important virulence factor in plant pathogens [37,38,39,40,41,42,43,44,45,46]. Horizontal transfer has led to hyper-variability among different strains within individual *Xanthomonas* species [47,48,49]. 

We previously reported [8] that the LPS locus in *Xcm* 4381 most closely matches that of *X.* *axonopodis* pv. *citri* (*Xac*) strain 306 [50], whereas half of the LPS locus in *Xvv* 702 was not detectably similar to *Xcm* 4381, but rather resembled that of *X. albilineans* strain GPE PC73 [8,51]. We [8] and subsequently others [52] pointed out that this pattern of sequence similarity is incongruent with the close phylogenetic relationship between *Xcm* 4381 and *Xvv* 702 and indicates recent horizontal transfer in one or both strains.

Sequencing of additional isolates indicated that the LPS biosynthesis gene cluster DNA sequence is highly conserved among *Xcm* isolates [7]. However, in the present study, additional genome sequencing revealed variation in this locus among isolates of *Xvv*. As illustrated in Figure 6, *Xvv* 895 shares 99% nucleotide sequence identity with isolates of *Xcm*, which, in turn, share 97% identity with *Xac* 306. However, in *Xvv* 206, 890, 1326 and 1381, the LPS cluster shares at least 99% identity to that of *Xvv* 702. Approximately one half of the LPS cluster in these *Xvv* isolates (adjacent to *etfA*) is 99% identical to that of *Xvv* 895 and *Xcm*. However, the other half (adjacent to *metB*) shares no detectable sequence similarity with *Xvv* 895, but it does share 84% identity with *X. sacchari* [53] and 86% with *X. albilineans* [8,51].

As Pieretti and colleagues noted [52], it is interesting that the *Xvv* 702-type LPS cluster is common to three distinct *Xanthomonas* species that all inhabit the xylem of sugarcane, and thus, there might be opportunities for these species to come into contact with each other and exchange genetic material. However, it is unlikely that this type of LPS is uniquely adapted to evading recognition as a pathogen-associated molecular pattern [54,55] by sugarcane, since *Xvv* 895, isolated from sugarcane, has a *Xac*-type LPS cluster, as does *Xcm*, which can also infect sugarcane [4]. Furthermore, *Xvv* 206 has the *Xvv* 702-type LPS cluster and was originally isolated from maize; this further emphasizes that there is not a clear correlation between LPS cluster type and host plant species. Drivers for variation in the LPS might be interactions with phages [36,37,56,57] or with insect vectors [58].

### 2.9. Xvv Isolates Differ in Their Repertoires of T3SS Effector Genes

The repertoire of T3SS effectors can significantly influence a bacterial phytopathogen’s host range [59,60,61,62]. Therefore, differences in T3SS repertoires between *Xcm* and *Xvv* are of great interest, as they might partly explain the ability of *Xcm* to cause disease in banana, whilst *Xvv* appears to be non-pathogenic in banana. We previously compared the set of T3SS effectors encoded by *Xcm* 4381 against the set encoded by *Xvv* 702 [8]. In that previous study, we identified only a few differences. Specifically, *Xcm* encodes two homologues of XopJ that are absent from the genome of *Xvv* 702, and *Xvv*702 encodes a homologue of XopAF that is absent from *Xcm* 4381 [8]. However, in the present study, utilizing additional sequence data, we found that XopAF is absent from the genome of *Xvv* 206, though it is present in *Xvv* 895, 890, 1326 and 1381, as well as in *Xvv* 702. It is absent from all sequenced isolates of *Xcm*.

#### 2.9.1. Gene for XopAF is Absent from Xvv 206, Isolated from Maize

We previously reported that *Xvv* 702 encodes a homologue of XopAF (GenBank: ACHS01000051.1, bases 7184–7783; RefSeq: WP_010364039) that is not present in the genome of *Xcm* 4381 [8]. This sequence shares 86% amino acid sequence identity with the XopAF (also known as AvrXv3) from *X. euvesicatoria* that was originally identified as an avirulence factor, inducing the hypersensitive response (HR) in resistant tomato and pepper plants [63]. It is also identical at the amino acid sequence level to proteins encoded by *X. translucens* pv. *translucens* (*Xtt*) DSM 18974 (RefSeq: WP_003475568). In the present study, we found that this *xopAF* gene is present in *Xvv* 895, 890, 1326 and 1381, as well as in *Xvv* 702. It is not present in *Xcm* nor in *Xvv* 206. Thus, XopAF is encoded by *Xvv* isolates from sugarcane, but not by the *Xvv* isolate from maize and not by *Xcm* isolates from banana and enset.

This begs the question of whether XopAF1 contributes to the limitation of host range in *Xvv*; that is, one might hypothesise that XopAF confers avirulence in banana and that the absence of XopAF in *Xcm* enables its pathogenicity in banana. In a recent comparative study of the genomes of different pathotypes of *X. citri* pv. *citri* (*Xca*) [64], the authors noted that XopAF was encoded in the genomes of a narrow-host-range strain, but absent from a closely related broad-host-range strain. Therefore, they hypothesised that XopAF might confer avirulence and contribute to the limitation of the host range. However, their mutational analysis showed that *xopAF* did not affect host range, but it did contribute to the ability of *X. citri* pv. *citri* pathotype A^w^ (*Xcaw*) to grow in a Mexican lime host plant. 

It should be noted that the predicted XopAF protein in *Xcaw* (RefSeq: WP_007652722.1) is much more divergent from the originally described sequence from *X. euvesicatoria* (WP_008577605.1), sharing only 31% amino acid sequence identity, whereas the *Xvv* protein shares 86% identity with *X. euvesicatoria* XopAF. Therefore, the *Xvv* XopAF protein is likely to interact with plants differently. Furthermore, the host plants in question are very different with *X. euvesicatoria* and *Xcaw* infecting dicots and *Xvv* and *Xcm* infecting monocots. However, it is reasonable to suppose that the *Xvv* protein is likely to be a T3SS effector and a potential avirulence factor, given the 86% identity between it and the experimentally characterised *X. euvesicatoria* XopAF [63]. XopAF contains a DNA-binding domain at its C terminus and may allow the pathogen to manipulate its host by affecting the expression of plant genes. It would be interesting to test whether heterologous expression of *xopAF* in *Xcm* would cause avirulence in banana and whether deletion of *xopAF* in *Xvv* would have any impact on virulence in sugarcane or maize.

In *Xvv* 702, the *xopAF* gene falls between Positions 7181 and 7543, on the reverse strand in GenBank accession ACHS01000051.1. This resides within a genomic region that also encodes several phage-associated proteins, including phage-related lytic enzyme, phage-tail protein, baseplate assembly protein J, phage-tail fibres and phage-tail fibre protein, and may result from the integration of a pro-phage into the *Xvv* genome. In the wheat pathogen, *Xtt* DSM 18974, the *xopAF* gene encoding an identical protein sequence is located in a different genomic context; it resides at Positions 40277 to 40933 in GenBank accession CAPJ01000122.1 (locus tag: BN444_00905). This region of the *Xtt* genome does not contain any obvious phage-related genes, but does contain a predicted transposase for insertion sequence element IS629 (locus tag: BN444_00906), suggesting a mechanism for the mobility of this gene.

#### 2.9.2. Gene Encoding Homologue of XopL Underwent Truncation in Common Ancestor of *Xvv* 890, 702, 1326 and 1381

We also found polymorphism with respect to a *xopL*-like gene in *Xvv*. The genomes of *Xvv* isolates 206 and 895 each encode a protein with 70% amino acid sequence identity to XopL from *X. campestris* pv. *vesicatoria* (*Xcv*) strain 85-10 (RefSeq: YP_364951.1) [65]. The GenBank accession numbers for the *Xvv* 895 and 206 sequences are AKBO01000570.1 (Positions 1804–3768) and AKBM01000211.1 (Positions 29591–31555), where they encode a full-length protein. However, in *Xvv* Isolates 890, 702, 1326 and 1381, there is a single-nucleotide C→Asubstitution resulting in a TCA codon being transformed to a TAA stop codon. This substitution occurs at Position 14191 in GenBank accession ACHS01000315.1 and is predicted to result in the protein being truncated to 265 amino acids (compared to the full-length 654 amino acids).

This *xopL*-like sequence is completely absent from *Xcm* isolates belonging to Sub-lineage II (*Xcm* 4379, 4380, 4381, 4383, 4384, 4392 and 4395). However, it is present in *Xcm* isolates belonging to Sub-lineage I (*Xcm* 2005, 2251, 4387 and 4389), where each genome encodes a full-length protein. Thus, it appears that this *xopL* homologue may have been lost twice: (i) completely deleted in a common ancestor of *Xcm* Sub-lineage II; and (ii) truncated by a premature stop codon in a common ancestor of *Xvv* 890, 702, 1326 and 1381.

It has recently been demonstrated that XopL possesses E3 ubiquitin ligase activity, induces plant cell death and subverts plant immunity and that the ligase activity is associated with the C-terminal region of the protein [66]. The premature stop codon found in *xopL* of some *Xvv* isolates has split the XopL-encoding open reading frame (ORF) into two.

Interestingly, there is a candidate plant inducible promoter (PIP) box [67,68,69,70] upstream of the second ORF, which corresponds to the C-terminal region of XopL, in which the E3 ubiquitin ligase resides. This suggests the hypothesis that this truncated ORF still has the potential to be expressed and be induced *in planta* and that it might still have biochemical activity, though it is unclear whether it would be a substrate of the T3SS. This potential PIP box (sequence TTCCGgcgaacatgcagcaaTTCGC) is located at Positions 14107 to 14137 in ACHS01000315.1, which is approximately 160 bp upstream of the C-terminal XopL ORF at 14296 to 15366. There is another PIP box (sequence TTCGCtacgataaagatgacTTCGC) located at 13300 to 13347, which is approximately 50 bp upstream of the ORF homologous to the XopL N terminus located at 13395 to 14192. The complete set of predicted PIP boxes in *Xvv* 702 and *Xcm* 4381 is tabulated in the Supplementary Material.

#### 2.9.3. Absence of Genes Encoding Homologues of XopJ Distinguishes *Xcm* from *Xvv*

In addition to the homologues of XopAF and XopL, a further two potential T3SS show differential presence/absence among our sequenced strains. We previously reported that *Xcm* 4381 encodes two homologues of XopJ that are absent from *Xvv* 702 [8]. Our subsequent analyses have confirmed that these are conserved in all the sequenced *Xcm* isolates and are absent from all the sequenced *Xvv*. Therefore, these predicted T3SS effectors remain as candidates for contributing to the differences in host range between *Xvv* and *Xcm*.

## 3. Experimental Section

### 3.1. Sources of Bacterial Strains

Bacterial strains were obtained from the National Collection of Plant Pathogenic Bacteria (NCPPB) at The Food and Environment Research Agency, UK (Fera). DNA library preparation and genome sequencing using the Illumina GA2x were performed using standard Illumina protocols, as previously described [7,8].

### 3.2. Preparation of Genomic DNA

For DNA preparation, bacterial strains were grown overnight at 28 °C in 10 mL King Broth shaken at 200 rpm. Bacterial cells were harvested by centrifugation and re-suspended in TE buffer (50 mM Tris-HCl, 40 mM EDTA, pH 8.0) containing 12 µL of 20 mg/mL lysozyme and 10 mg/mL RNase and incubated at 25 °C for 10 min with 17 µL 10% sodium dodecyl sulphate, then incubated on ice for 5 min. Proteins were dissolved with 170 µL of 8 M ammonium acetate, vortexed vigorously for 30 s centrifuged at 4 °C and for 15 min. DNA was precipitated with isopropanol and re-dissolved in 100 μL of 10 mM Tris, pH 8.0, and 1 mM Na_2_EDTA.

### 3.3. Genome Sequencing

We used the Illumina GA2x platform to sequence genomes of *Xvv* strains NCPPB 895 and 890, generating paired sequence reads of length 67 nucleotides, according to the manufacturer’s instructions.

### 3.4. Alignment of Sequence Reads Against Reference Genome Sequences

We used BWA [14] to align GA2x sequence reads against a reference genome sequence and used IGV [71] to visualize the alignments and SAMtools [72] to manipulate the alignments and convert between formats.

### 3.5. SNP Calling and Phylogenetic Analysis

We used a very conservative approach to infer SNPs from the alignments of Illumina reads against the previously published *Xoo* reference draft genome assembly. To avoid false positives and false negatives, we only used those regions of the *Xoo* genome with a coverage depth of 10 or more for every sequenced *Xcm* and *Xvv* genome and where there was at least 95% consensus among the sequence reads within each isolate. Just over 30% of the length (1,507,606 out of 4,940,217 nt) of the *Xoo* genome fulfilled these two criteria. In other words, for 30% of the *Xoo* chromosome, there was sufficient quantity and consistency in our data to be almost certain of the sequence in all of the eight isolates (six *Xvv* and two *Xcm*; see Figure 1); for the remaining 70% of the genome, there was some degree of ambiguity in the data for one or more of the isolates. The phylogenetic tree was inferred using the Maximum Parsimony method implemented in MEGA5 [73] based on 39,665 single-nucleotide variants with respect to the chromosome of *Xoo* MAFF 311018. Bootstrap values were calculated as percentages of 500 trials. 

### 3.6. Genome Assembly

*De novo* assembly of Illumina sequence reads was performed using Velvet 1.1.04 [74]. We discarded any sequence reads that contained one or more “N” prior to assembly. It is difficult or impossible to predict the optimal parameter values for Velvet assembly. Therefore, we generated assemblies using a range of combinations of hash length and coverage cut-off and chose the assemblies giving the largest N_50_ values. For the newly presented assemblies (*i.e.,* for *Xvv* 890 and 895), the parameter values were for *Xvv* 895: hash length = 25 and coverage cut-off = 2 and for *Xvv* 890: hash length = 29 and coverage cut-off = 3.

### 3.7. Identification of Presence and Absence of Genes

We used BEDtools [15] to infer the breadths of coverage for genomic features based on Binary Alignment Map (BAM) files from the Burrows-Wheeler Aligner (BWA) and General Feature Format (GFF) files from Rapid Annotation using Subsystem Technology (RAST) [75]. We used the pheatmap package in R to generate heatmaps [76]. However, it should be noted that incompleteness of *de novo* assemblies do not invalidate these, since our inferences are based upon comparisons of alignments of raw reads rather than comparisons between assemblies; these alignments consist of unassembled read-pairs aligned with BWA against various reference genome sequences, and several examples of such alignments are illustrated in the Supplementary Material. The single exception is Figure 2, in which the comparison consists of alignments between *de novo* assemblies; it is possible that some gaps in the alignments in Figure 2 could arise through the incompleteness of the *de novo* assemblies. Figure 4 and Figure 5 are based on BWA alignments between de novo assemblies, but the findings were also validated by the inspection of alignments of raw (unassembled) sequence reads (see the Supplementary Material).

### 3.8. Visualisation of Genome-Wide Patterns of Sequence Conservation

We used BLASTN [77] to align assembled sequences and visualized the alignments using the Artemis Comparison Tool [78] and BLAST Ring Image Generator (BRIG) [79], which is a wrapper for Circular Genome Viewer (CGView) [80].

### 3.9. Identification of Potential PIP Boxes

We built a profile hidden Markov model (HMM) based on a multiple sequence alignment of 22 known PIP boxes from *X. vesicatoria* (from Table 3 in [69]) using hmmb from the HMMER 1.8.5 package [81]. The DNA sequence was scanned against this profile-HMM using hmmls from HMMER 1.8.5 with a bit-score cut-off of 10.0. 

## 4. Conclusions

Here, we analyse draft genome sequences for two isolates of *Xvv* to augment the four previously published [7,8] draft genome sequences of *Xvv*. Comparative analyses of these genome sequences and previously published genome sequences of the closely related pathovar, *Xcm*, have revealed extensive differences in gene content among *Xvv*. This manuscript describes some of these differences in detail, including differences in plasmid content, LPS biosynthesis clusters, T4P and T3SS effectors. The main evolutionary events are summarized graphically in Figure 1. As well as providing some insight into evolutionary events within *Xvv*, these sequence analyses also further refine our understanding of the genomic differences between *Xvv* and the very closely related *Xcm*, which is a recently emerging pathogen in banana and enset; the availability of the sequence from multiple isolates allows us to distinguish between inherent variation within *Xvv* that might confound attempts to identify important genetic differences between the two pathovars and for functional analysis of important virulence factors.

It is clear that *Xcm* is genetically highly monomorphic [5,6,7]; here, we show that, apart from several phage-related genes and the SNPs in the core genome described previously [7], the few genetic differences among *Xcm* isolates can be explained by the acquisition of a plasmid in Xcm Sub-lineage II, which is not present in Sub-lineage I, nor in at least one isolate of Sub-lineage II (*Xcm* 4381). Additionally, the two *Xcm* sub-lineages differ in that members of Sub-lineage II have lost a gene encoding a homologue of XopL; interestingly, this gene has acquired a premature stop codon in four of the six *Xvv* isolates, suggesting that isolates of both *Xcm* and *Xvv* have independently converged on eliminating XopL.

In contrast to the limited genetic diversity within *Xcm*, there is considerable diversity within *Xvv*, both at the level of SNPs in the core genome and at the level of gene content. Some of the differences in gene content are ascribable to the acquisition of two different plasmids (one in *Xvv* 895 and one in *Xvv* 890, 702, 1326 and 1381), but there are also differences in chromosomally located gene clusters, such as those encoding LPS biosynthesis and T4P.

Overall, this work suggests hypotheses for future work towards understanding the molecular basis for the ability of *Xcm* to emerge as an important pathogen of banana and enset. For example, one consistent difference is that all sequenced *Xcm* isolates encode two homologues of XopJ that are absent from all sequenced isolates of *Xvv*. In *X. campestris* pv. *vesicatoria*, this T3SS effector has been shown to interfere with salicylic acid-dependent defence responses to attenuate the onset of necrosis and to alter host transcription [82]; it will be enlightening to test the contribution of the two XopJ homologues in *Xcm* interaction with banana and enset. Furthermore, understanding the emergence of Xcm will require the study of genome sequences of a wider range of strains within the species, *X. vasicola*, to which *Xcm* probably belongs [4]; several isolates are available in strain collections for *X. vasicola* pv. *holcicola* [83], but there is also a need to survey other as yet unknown members of the species that might inhabit the centre of origin, perhaps colonizing other monocot plants.

## Supplementary Files

Supplementary File 1 Supplementary Materials (TAR, 5271 KB)
