# Supplementary material for: Genome Sequencing of Xanthomonas vasicola Pathovar vasculorum Reveals Variation in Plasmids and Genes Encoding Lipopolysaccharide Synthesis, Type-IV Pilus and Type-III Secretion Effectors
Source: Pathogens. 2014 Mar 18;3(1):211–37. doi: 10.3390/pathogens3010211 (PMC4235730; doi:10.3390/pathogens3010211)
Supplement: Supplementary File 1 — Supplementary Materials (TAR, 5271 KB) [file pathogens-03-00211-s001.tar › supplementary/index.html]

Genome sequencing of Xanthomonas vasicola pathovar vasculorum reveals variation in lipopolysaccharide synthesis, type-IV pilus, plasmids and type-III secretion effectors


## Genome sequencing of *Xanthomonas vasicola* pathovar *vasculorum* reveals variation in lipopolysaccharide synthesis, type-IV pilus, plasmids and type-III secretion effectors

### Supplementary data for manuscript by Wasukira *et al*.

|  |  |
| --- | --- |
| Phylogenetic relationships between *Xvv*, *Xcm* and *Xoo*. The phylogenetic tree on the left was inferred using the Maximum Parsimony method based on 39,665 single-nucleotide variants with respect to the chromosome of Xoo MAFF 311018. Bootstrap values are expressed as percentages of 500 trials. The tree is drawn to scale with branch lengths calculated using the average pathway method and are in the units of the number of changes over the whole sequence. |  |
| Figure S1. Comparison of gene-content based on alignment against the *Xcm* NCPPB 2005 genome assembly. |  |
| Figure S2. Comparison of gene-content based on alignment against the *Xcm* NCPPB 4384 genome assembly. |  |
| Figure S3. Comparison of gene-content based on alignment against the *Xvv* NCPPB 895 genome assembly. |  |
| Figure S4. Comparison of gene-content based on alignment against the *Xvv* NCPPB 206 genome assembly. |  |
| Figure S5. Comparison of gene-content based on alignment against the *Xvv* NCPPB 1326 genome assembly. |  |
| Figure S6. The genomes of *Xvv* NCPPB 890, 702, 1326 and 1381 contain extensive sequence similarity to *X. axonopodis* pv. *citri* 29-1 plasmid pXAC47. |  |
| Figure S7. The genomes of *Xcm* NCPPB 4379, 4380, 4383, 4384, 4392 and 4395 each contain extensive sequence similarity to *X. citri* pv. *malvacearum* X20 unnamed plasmid. Illumina genomic sequence reads are aligned against CM002030.1. |  |
| Figure S8. The genomes of *Xcm* NCPPB 895 contains extensive sequence similarity to plasmid sequences from *X. citri* pv. *manihotis*. Illumina genomic DNA sequence reads are aligned against AKEJ01000091.1. |  |
| Figure S9. *Xvv* NCPPB 206, 890, 702, 1326 and 1381 share a common LPS biosynthesis cluster that is distinct from that of *Xvv* 895 and *Xcm*. Illumina genomic sequence reads are aligned against ACHS01000119.1 | . |
| Figure S10. *Xvv* NCPPB 206, 890, 702, 1326 and 1381 share a common LPS biosynthesis cluster that is distinct from that of *Xvv* 895 and *Xcm*. Illumina genomic sequence reads are aligned against ACHS01000380.1. |  |
| Figure S11. *Xvv* NCPPB 895 and *Xcm* share a common LPS biosynthesis cluster that is distinct from those of *Xvv* NCPPB 206, 890, 702, 1326 and 1381. Illumina genomic sequence reads are aligned against AKBE01000022.1. |  |
| Figure S12. The TFP loci of *Xvv* NCPPB 890, 702, 1326 and 1381 are similar to each other but different to those of *Xvv* NCPPB 895 and 206. Illumina genomic DNA sequence reads are aligned against ACHS01000345.1. |  |
| Figure S13. The TFP loci of *Xvv* NCPPB 895 and 206 are similar to those of *Xcm* and different from those of *Xvv* NCPPB 890, 702, 1326 and 1381. Illumina genomic DNA sequence reads are aligned against AKBM01000028.1. |  |
| Figure S14. *Xvv* 702, 890 1326 and 1381 contain *xopAF*-like gene that is absent from *Xvv* 206 and from *Xcm*. Illumina genomic DNA sequence reads are aligned against ACHS01000051.1. |  |
| Figure S15. *Xcm* genomes contain a xopJ-like gene that is absent from *Xvv* genomes. Illumina genomic DNA sequence reads are aligned against AKBE00000192.1. |  |
| Figure S16. *Xcm* genomes contain a xopJ-like gene that is absent from *Xvv* genomes. Illumina genomic DNA sequence reads are aligned against AKBE00000064.1. |  |
| Figure S17. A *xopL*-like gene is present in *Xcm* 2005, 2251, 4387, and 4389 but absent from *Xcm* 4379, 4380, 4381, 4383, 4384, 4394 and 4395. Illumina genomic DNA sequence reads are aligned against AKBE00000144.1. |  |
| Figure S18. The *xopL*-like gene contains a C->A substitution resulting in a TCA codon being transformed to a TAA stop codon in *Xvv* 890, 702, 1326 and 1381. Illumina genomic DNA sequence reads are aligned against AKBE00000144.1. |  |
| Figure S19. The *xopL*-like gene contains a C->A substitution resulting in a TCA codon being transformed to a TAA stop codon in *Xvv* 890, 702, 1326 and 1381. Illumina genomic DNA sequence reads are aligned against ACHS01000315.1. |  |
| Data file 1. Multiple sequence alignment of SNPs on which phylogentic analysis in the manuscript is based. In FastA format. | FastA file |
| Data file 2. Candidate PIP boxes in the genome sequence of *Xvv* 702. | *Xvv* 702 PIP boxes |
| Data file 3. Candidate PIP boxes in the genome sequence of *Xcm* 4381. | *Xcm* 4381 PIP boxes |
