# Supplementary material for: Genome Sequencing of Xanthomonas vasicola Pathovar vasculorum Reveals Variation in Plasmids and Genes Encoding Lipopolysaccharide Synthesis, Type-IV Pilus and Type-III Secretion Effectors
Source: Pathogens. 2014 Mar 18;3(1):211–37. doi: 10.3390/pathogens3010211 (PMC4235730; doi:10.3390/pathogens3010211)
Supplement: Supplementary File 1 — Supplementary Materials (TAR, 5271 KB) [file pathogens-03-00211-s001.tar › supplementary/images/gene-content-versus-Xcm2005.pdf]

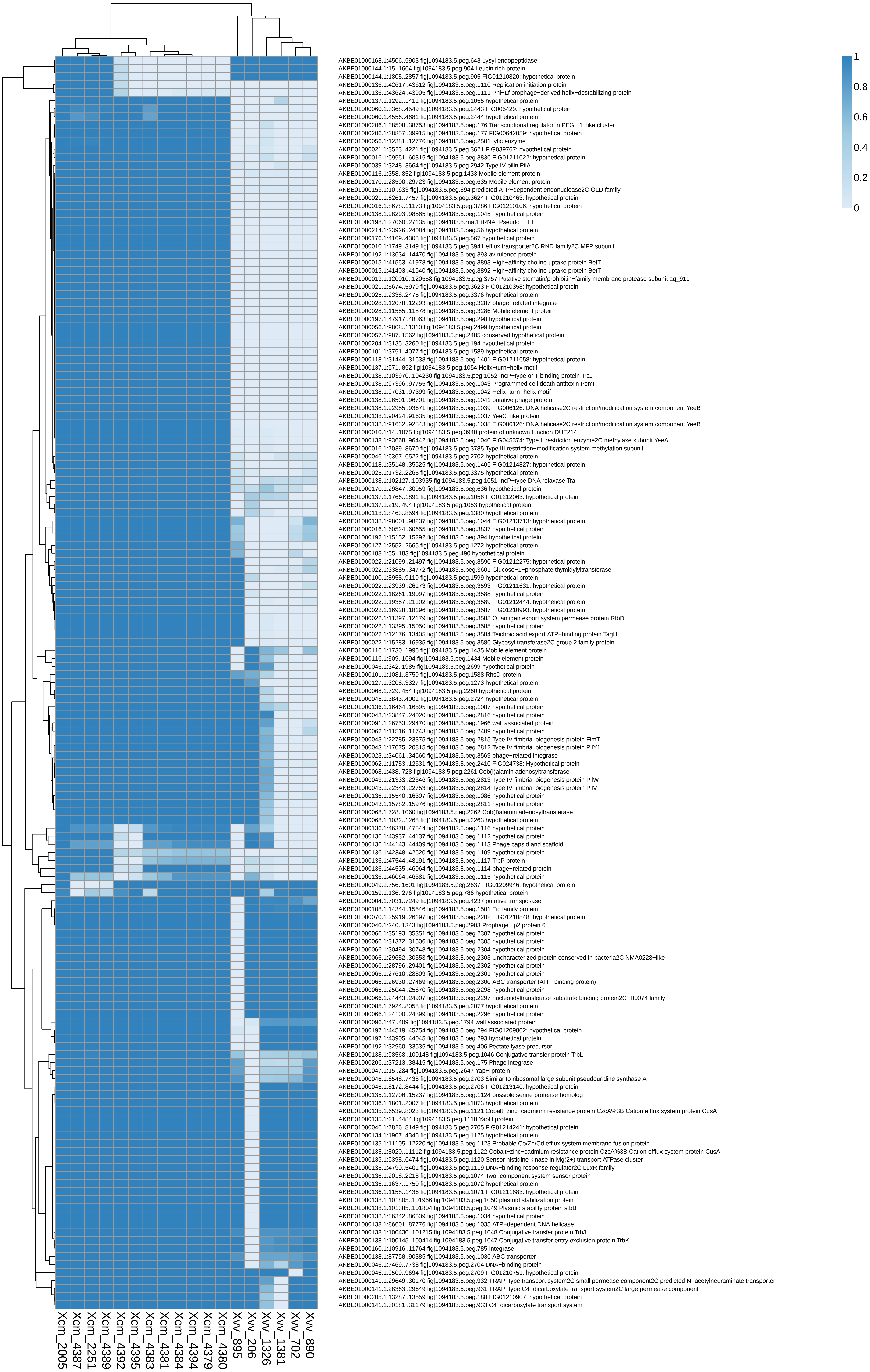

AKBE01000168.1:4506..5903 fig|1094183.5.peg.643 Lysyl endopeptidase
AKBE01000144.1:15..1664 fig|1094183.5.peg.904 Leucin rich protein
AKBE01000144.1:1805..2857 fig|1094183.5.peg.905 FIG01210820: hypothetical protein
AKBE01000136.1:42617..43612 fig|1094183.5.peg.1110 Replication initiation protein
AKBE01000136.1:43624..43905 fig|1094183.5.peg.1111 Phi−Lf prophage−derived helix−destabilizing protein
AKBE01000137.1:1292..1411 fig|1094183.5.peg.1055 hypothetical protein
AKBE01000060.1:3368..4549 fig|1094183.5.peg.2443 FIG005429: hypothetical protein
AKBE01000060.1:4556..4681 fig|1094183.5.peg.2444 hypothetical protein
AKBE01000206.1:38508..38753 fig|1094183.5.peg.176 Transcriptional regulator in PFGI−1−like cluster
AKBE01000206.1:38857..39915 fig|1094183.5.peg.177 FIG00642059: hypothetical protein
AKBE01000056.1:12381..12776 fig|1094183.5.peg.2501 lytic enzyme
AKBE01000021.1:3523..4221 fig|1094183.5.peg.3621 FIG039767: hypothetical protein
AKBE01000016.1:59551..60315 fig|1094183.5.peg.3836 FIG01211022: hypothetical protein
AKBE01000039.1:3248..3664 fig|1094183.5.peg.2942 Type IV pilin PilA
AKBE01000116.1:358..852 fig|1094183.5.peg.1433 Mobile element protein
AKBE01000170.1:28500..29723 fig|1094183.5.peg.635 Mobile element protein
AKBE01000153.1:10..633 fig|1094183.5.peg.894 predicted ATP−dependent endonuclease2C OLD family
AKBE01000021.1:6261..7457 fig|1094183.5.peg.3624 FIG01210463: hypothetical protein
AKBE01000016.1:8678..11173 fig|1094183.5.peg.3786 FIG01210106: hypothetical protein
AKBE01000138.1:98293..98565 fig|1094183.5.peg.1045 hypothetical protein
AKBE01000198.1:27060..27135 fig|1094183.5.rna.1 tRNA−Pseudo−TTT
AKBE01000214.1:23926..24084 fig|1094183.5.peg.56 hypothetical protein
AKBE01000176.1:4169..4303 fig|1094183.5.peg.567 hypothetical protein
AKBE01000010.1:1749..3149 fig|1094183.5.peg.3941 efflux transporter2C RND family2C MFP subunit
AKBE01000192.1:13634..14470 fig|1094183.5.peg.393 avirulence protein
AKBE01000015.1:41553..41978 fig|1094183.5.peg.3893 High−affinity choline uptake protein BetT
AKBE01000015.1:41403..41540 fig|1094183.5.peg.3892 High−affinity choline uptake protein BetT
AKBE01000019.1:120010..120558 fig|1094183.5.peg.3757 Putative stomatin/prohibitin−family membrane protease subunit aq_911
AKBE01000021.1:5674..5979 fig|1094183.5.peg.3623 FIG01210358: hypothetical protein
AKBE01000025.1:2338..2475 fig|1094183.5.peg.3376 hypothetical protein
AKBE01000028.1:12078..12293 fig|1094183.5.peg.3287 phage−related integrase
AKBE01000028.1:11555..11878 fig|1094183.5.peg.3286 Mobile element protein
AKBE01000197.1:47917..48063 fig|1094183.5.peg.298 hypothetical protein
AKBE01000056.1:9808..11310 fig|1094183.5.peg.2499 hypothetical protein
AKBE01000057.1:987..1562 fig|1094183.5.peg.2485 conserved hypothetical protein
AKBE01000204.1:3135..3260 fig|1094183.5.peg.194 hypothetical protein
AKBE01000101.1:3751..4077 fig|1094183.5.peg.1589 hypothetical protein
AKBE01000118.1:31444..31638 fig|1094183.5.peg.1401 FIG01211658: hypothetical protein
AKBE01000137.1:571..852 fig|1094183.5.peg.1054 Helix−turn−helix motif
AKBE01000138.1:103970..104230 fig|1094183.5.peg.1052 IncP−type oriT binding protein TraJ
AKBE01000138.1:97396..97755 fig|1094183.5.peg.1043 Programmed cell death antitoxin PemI
AKBE01000138.1:97031..97399 fig|1094183.5.peg.1042 Helix−turn−helix motif
AKBE01000138.1:96501..96701 fig|1094183.5.peg.1041 putative phage protein
AKBE01000138.1:92955..93671 fig|1094183.5.peg.1039 FIG006126: DNA helicase2C restriction/modification system component YeeB
AKBE01000138.1:90424..91635 fig|1094183.5.peg.1037 YeeC−like protein
AKBE01000138.1:91632..92843 fig|1094183.5.peg.1038 FIG006126: DNA helicase2C restriction/modification system component YeeB
AKBE01000010.1:14..1075 fig|1094183.5.peg.3940 protein of unknown function DUF214
AKBE01000138.1:93668..96442 fig|1094183.5.peg.1040 FIG045374: Type II restriction enzyme2C methylase subunit YeeA
AKBE01000016.1:7039..8670 fig|1094183.5.peg.3785 Type III restriction−modification system methylation subunit
AKBE01000046.1:6367..6522 fig|1094183.5.peg.2702 hypothetical protein
AKBE01000118.1:35148..35525 fig|1094183.5.peg.1405 FIG01214827: hypothetical protein
AKBE01000025.1:1732..2265 fig|1094183.5.peg.3375 hypothetical protein
AKBE01000138.1:102127..103935 fig|1094183.5.peg.1051 IncP−type DNA relaxase TraI
AKBE01000170.1:29847..30059 fig|1094183.5.peg.636 hypothetical protein
AKBE01000137.1:1766..1891 fig|1094183.5.peg.1056 FIG01212063: hypothetical protein
AKBE01000137.1:219..494 fig|1094183.5.peg.1053 hypothetical protein
AKBE01000118.1:8463..8594 fig|1094183.5.peg.1380 hypothetical protein
AKBE01000138.1:98001..98237 fig|1094183.5.peg.1044 FIG01213713: hypothetical protein
AKBE01000016.1:60524..60655 fig|1094183.5.peg.3837 hypothetical protein
AKBE01000192.1:15152..15292 fig|1094183.5.peg.394 hypothetical protein
AKBE01000127.1:2552..2665 fig|1094183.5.peg.1272 hypothetical protein
AKBE01000188.1:55..183 fig|1094183.5.peg.490 hypothetical protein
AKBE01000022.1:21099..21497 fig|1094183.5.peg.3590 FIG01212275: hypothetical protein
AKBE01000022.1:33885..34772 fig|1094183.5.peg.3601 Glucose−1−phosphate thymidylyltransferase
AKBE01000100.1:8958..9119 fig|1094183.5.peg.1599 hypothetical protein
AKBE01000022.1:23939..26173 fig|1094183.5.peg.3593 FIG01211631: hypothetical protein
AKBE01000022.1:18261..19097 fig|1094183.5.peg.3588 hypothetical protein
AKBE01000022.1:19357..21102 fig|1094183.5.peg.3589 FIG01212444: hypothetical protein
AKBE01000022.1:16928..18196 fig|1094183.5.peg.3587 FIG01210993: hypothetical protein
AKBE01000022.1:11397..12179 fig|1094183.5.peg.3583 O−antigen export system permease protein RfbD
AKBE01000022.1:13395..15050 fig|1094183.5.peg.3585 hypothetical protein
AKBE01000022.1:12176..13405 fig|1094183.5.peg.3584 Teichoic acid export ATP−binding protein TagH
AKBE01000022.1:15283..16935 fig|1094183.5.peg.3586 Glycosyl transferase2C group 2 family protein
AKBE01000116.1:1730..1996 fig|1094183.5.peg.1435 Mobile element protein
AKBE01000116.1:909..1694 fig|1094183.5.peg.1434 Mobile element protein
AKBE01000046.1:342..1985 fig|1094183.5.peg.2699 hypothetical protein
AKBE01000101.1:1081..3759 fig|1094183.5.peg.1588 RhsD protein
AKBE01000127.1:3208..3327 fig|1094183.5.peg.1273 hypothetical protein
AKBE01000068.1:329..454 fig|1094183.5.peg.2260 hypothetical protein
AKBE01000045.1:3843..4001 fig|1094183.5.peg.2724 hypothetical protein
AKBE01000136.1:16464..16595 fig|1094183.5.peg.1087 hypothetical protein
AKBE01000043.1:23847..24020 fig|1094183.5.peg.2816 hypothetical protein
AKBE01000091.1:26753..29470 fig|1094183.5.peg.1966 wall associated protein
AKBE01000062.1:11516..11743 fig|1094183.5.peg.2409 hypothetical protein
AKBE01000043.1:22785..23375 fig|1094183.5.peg.2815 Type IV fimbrial biogenesis protein FimT
AKBE01000043.1:17075..20815 fig|1094183.5.peg.2812 Type IV fimbrial biogenesis protein PilY1
AKBE01000023.1:34061..34660 fig|1094183.5.peg.3569 phage−related integrase
AKBE01000062.1:11753..12631 fig|1094183.5.peg.2410 FIG024738: Hypothetical protein
AKBE01000068.1:438..728 fig|1094183.5.peg.2261 Cob(I)alamin adenosyltransferase
AKBE01000043.1:21333..22346 fig|1094183.5.peg.2813 Type IV fimbrial biogenesis protein PilW
AKBE01000043.1:22343..22753 fig|1094183.5.peg.2814 Type IV fimbrial biogenesis protein PilV
AKBE01000136.1:15540..16307 fig|1094183.5.peg.1086 hypothetical protein
AKBE01000043.1:15782..15976 fig|1094183.5.peg.2811 hypothetical protein
AKBE01000068.1:728..1060 fig|1094183.5.peg.2262 Cob(I)alamin adenosyltransferase
AKBE01000068.1:1032..1268 fig|1094183.5.peg.2263 hypothetical protein
AKBE01000136.1:46378..47544 fig|1094183.5.peg.1116 hypothetical protein
AKBE01000136.1:43937..44137 fig|1094183.5.peg.1112 hypothetical protein
AKBE01000136.1:44143..44409 fig|1094183.5.peg.1113 Phage capsid and scaffold
AKBE01000136.1:42348..42620 fig|1094183.5.peg.1109 hypothetical protein
AKBE01000136.1:47544..48191 fig|1094183.5.peg.1117 TrbP protein
AKBE01000136.1:44535..46064 fig|1094183.5.peg.1114 phage−related protein
AKBE01000136.1:46064..46381 fig|1094183.5.peg.1115 hypothetical protein
AKBE01000049.1:756..1601 fig|1094183.5.peg.2637 FIG01209946: hypothetical protein
AKBE01000159.1:136..276 fig|1094183.5.peg.786 hypothetical protein
AKBE01000004.1:7031..7249 fig|1094183.5.peg.4237 putative transposase
AKBE01000108.1:14344..15546 fig|1094183.5.peg.1501 Fic family protein
AKBE01000070.1:25919..26197 fig|1094183.5.peg.2202 FIG01210848: hypothetical protein
AKBE01000040.1:240..1343 fig|1094183.5.peg.2903 Prophage Lp2 protein 6
AKBE01000066.1:35193..35351 fig|1094183.5.peg.2307 hypothetical protein
AKBE01000066.1:31372..31506 fig|1094183.5.peg.2305 hypothetical protein
AKBE01000066.1:30494..30748 fig|1094183.5.peg.2304 hypothetical protein
AKBE01000066.1:29652..30353 fig|1094183.5.peg.2303 Uncharacterized protein conserved in bacteria2C NMA0228−like
AKBE01000066.1:28796..29401 fig|1094183.5.peg.2302 hypothetical protein
AKBE01000066.1:27610..28809 fig|1094183.5.peg.2301 hypothetical protein
AKBE01000066.1:26930..27469 fig|1094183.5.peg.2300 ABC transporter (ATP−binding protein)
AKBE01000066.1:25044..25670 fig|1094183.5.peg.2298 hypothetical protein
AKBE01000066.1:24443..24907 fig|1094183.5.peg.2297 nucleotidyltransferase substrate binding protein2C HI0074 family
AKBE01000085.1:7924..8058 fig|1094183.5.peg.2077 hypothetical protein
AKBE01000066.1:24100..24399 fig|1094183.5.peg.2296 hypothetical protein
AKBE01000096.1:47..409 fig|1094183.5.peg.1794 wall associated protein
AKBE01000197.1:44519..45754 fig|1094183.5.peg.294 FIG01209802: hypothetical protein
AKBE01000197.1:43905..44045 fig|1094183.5.peg.293 hypothetical protein
AKBE01000192.1:32960..33535 fig|1094183.5.peg.406 Pectate lyase precursor
AKBE01000138.1:98568..100148 fig|1094183.5.peg.1046 Conjugative transfer protein TrbL
AKBE01000206.1:37213..38415 fig|1094183.5.peg.175 Phage integrase
AKBE01000047.1:15..284 fig|1094183.5.peg.2647 YapH protein
AKBE01000046.1:6548..7438 fig|1094183.5.peg.2703 Similar to ribosomal large subunit pseudouridine synthase A
AKBE01000046.1:8172..8444 fig|1094183.5.peg.2706 FIG01213140: hypothetical protein
AKBE01000135.1:12706..15237 fig|1094183.5.peg.1124 possible serine protease homolog
AKBE01000136.1:1801..2007 fig|1094183.5.peg.1073 hypothetical protein
AKBE01000135.1:6539..8023 fig|1094183.5.peg.1121 Cobalt−zinc−cadmium resistance protein CzcA%3B Cation efflux system protein CusA
AKBE01000135.1:21..4484 fig|1094183.5.peg.1118 YapH protein
AKBE01000046.1:7826..8149 fig|1094183.5.peg.2705 FIG01214241: hypothetical protein
AKBE01000134.1:1907..4345 fig|1094183.5.peg.1125 hypothetical protein
AKBE01000135.1:11105..12220 fig|1094183.5.peg.1123 Probable Co/Zn/Cd efflux system membrane fusion protein
AKBE01000135.1:8020..11112 fig|1094183.5.peg.1122 Cobalt−zinc−cadmium resistance protein CzcA%3B Cation efflux system protein CusA
AKBE01000135.1:5398..6474 fig|1094183.5.peg.1120 Sensor histidine kinase in Mg(2+) transport ATPase cluster
AKBE01000135.1:4790..5401 fig|1094183.5.peg.1119 DNA−binding response regulator2C LuxR family
AKBE01000136.1:2018..2218 fig|1094183.5.peg.1074 Two−component system sensor protein
AKBE01000136.1:1637..1750 fig|1094183.5.peg.1072 hypothetical protein
AKBE01000136.1:1158..1436 fig|1094183.5.peg.1071 FIG01211683: hypothetical protein
AKBE01000138.1:101805..101966 fig|1094183.5.peg.1050 plasmid stabilization protein
AKBE01000138.1:101385..101804 fig|1094183.5.peg.1049 Plasmid stability protein stbB
AKBE01000138.1:86342..86539 fig|1094183.5.peg.1034 hypothetical protein
AKBE01000138.1:86601..87776 fig|1094183.5.peg.1035 ATP−dependent DNA helicase
AKBE01000138.1:100430..101215 fig|1094183.5.peg.1048 Conjugative transfer protein TrbJ
AKBE01000138.1:100145..100414 fig|1094183.5.peg.1047 Conjugative transfer entry exclusion protein TrbK
AKBE01000160.1:10916..11764 fig|1094183.5.peg.785 Integrase
AKBE01000138.1:87758..90385 fig|1094183.5.peg.1036 ABC transporter
AKBE01000046.1:7469..7738 fig|1094183.5.peg.2704 DNA−binding protein
AKBE01000046.1:9509..9694 fig|1094183.5.peg.2709 FIG01210751: hypothetical protein
AKBE01000141.1:29649..30170 fig|1094183.5.peg.932 TRAP−type transport system2C small permease component2C predicted N−acetylneuraminate transporter
AKBE01000141.1:28363..29649 fig|1094183.5.peg.931 TRAP−type C4−dicarboxylate transport system2C large permease component
AKBE01000205.1:13287..13559 fig|1094183.5.peg.188 FIG01210907: hypothetical protein
AKBE01000141.1:30181..31179 fig|1094183.5.peg.933 C4−dicarboxylate transport system
Xcm_2005
Xcm_4387
Xcm_2251
Xcm_4389
Xcm_4392
Xcm_4395
Xcm_4383
Xcm_4381
Xcm_4384
Xcm_4394
Xcm_4379
Xcm_4380
Xvv_895
Xvv_206
Xvv_1326
Xvv_1381
Xvv_702
Xvv_890
1
0.8
0.6
0.4
0.2
0
