# Supplementary material for: Genome Sequencing of Xanthomonas vasicola Pathovar vasculorum Reveals Variation in Plasmids and Genes Encoding Lipopolysaccharide Synthesis, Type-IV Pilus and Type-III Secretion Effectors
Source: Pathogens. 2014 Mar 18;3(1):211–37. doi: 10.3390/pathogens3010211 (PMC4235730; doi:10.3390/pathogens3010211)
Supplement: Supplementary File 1 — Supplementary Materials (TAR, 5271 KB) [file pathogens-03-00211-s001.tar › supplementary/data/Xcm4381_PIP-boxes.html]

Xcm 4381 predicted PIP boxes


| Position | Hrp box sequence | Hrp box HMMER score | Downstream genes |
| --- | --- | --- | --- |
| ACHT01000457:4577-4639 | ttcgtcttcccagccaggacttcggtgaagcgaagcgctcccctgcaaacttgcttctacaat | 19.3 | - XcampmN\_010100014133 (ACHT01000457:4264-2177) putative type III effector HolPsyAE |
| ACHT01000628:2879-2939 | ttcgtcgtcccagccagaacttcggtgaggccaagcgaatcggagaactagtcaccaaact | 18.4 | - XcampmN\_010100019773 (ACHT01000628:3255-4046) avirulence protein |
| ACHT01000507:1965-1995 | **TTCGC**cggccgagccgttacttcagtaacgt | 18.02 | - XcampmN\_010100016182 (ACHT01000507:2081-3323) membrane fusion protein COG0845 Membrane-fusion protein |
| ACHT01000438:16078-16138 | **TTCGC**cgtcccaaccatgacttcgggcaaaaaacgacattcagaaagctctgcgtcaaact | 17.46 | - XcampmN\_010100013698 (ACHT01000438:16779-18326) outer protein B |
| ACHT01000065:6559-6621 | **TTCGC**catctcgcgcagcgt**TTCGC**atccgaagttttgcacgcagcgcagttccacttacatt | 16.6 | - XcampmN\_010100001944 (ACHT01000065:7200-9062) type III secretion system effector |
| ACHT01000215:14295-14356 | **TTCGC**cggaccacctattgc**TTCGC**tttagcagcagctcgccagatgcctgtgaggtacttt | 16.58 | - XcampmN\_010100006715 (ACHT01000215:13974-13168) HrcQ protein COG1886 Flagellar motor switch/type III secretory pathway protein - XcampmN\_010100006725 (ACHT01000215:14853-14215) HpaC protein |
| ACHT01000216:5293-5333 | **TTCGC**ttgcttgctaagtgc**TTCGC**cggacctgagtaaatt | 16.02 | - XcampmN\_010100006800 (ACHT01000216:5440-5997) Hpa2 COG0741 Soluble lytic murein transglycosylase and related regulatory proteins (some contain LysM/invasin domains) |
| ACHT01000059:3690-3765 | ttcgccttcatgtccatgac**TTCGC**cttctcccgttcatgggaaacg**TTCGC**ctgcaattcaggcatgcctacggt | 15.3 |  |
| ACHT01000215:17889-17951 | **TTCGC**caaaccatgcaggtg**TTCGC**ctgaaagacgggctgactcgcgacggctctaccagaat | 14.99 | - XcampmN\_010100006735 (ACHT01000215:17857-16784) type III secretion system protein HrcU COG1377 Flagellar biosynthesis pathway, component FlhB |
| ACHT01000261:764-799 | **TTCGC**acgcagacgggggac**TTCGC**gctgataagct | 14.98 | - XcampmN\_010100008780 (ACHT01000261:732-1) aminopeptidase COG2234 Predicted aminopeptidases |
| ACHT01000216:5193-5226 | **TTCGC**acgcacaagcgcaat**TTCGC**aaacactct | 14.62 | - XcampmN\_010100006795 (ACHT01000216:5069-4650) hypothetical protein |
| ACHT01000117:1659-1720 | ttcgtcagcgcacacaggagttcaccgcgccgccatgtccttgtgctggaggagggtaacgt | 14.54 | - XcampmN\_010100004132 (ACHT01000117:1952-3298) hypothetical protein COG1957 Inosine-uridine nucleoside N-ribohydrolase |
| ACHT01000662:6505-6544 | **TTCGC**catcgcgcagaccta**TTCGC**gcctggtgatcgatt | 14.5 | - XcampmN\_010100020988 (ACHT01000662:7069-7239) hypothetical protein - XcampmN\_010100020983 (ACHT01000662:6262-7041) hypothetical protein COG3931 Predicted N-formylglutamate amidohydrolase |
| ACHT01000499:12370-12401 | **TTCGC**gcagcagatcgatctc**TTCGC**caagct | 14.39 | - XcampmN\_010100015887 (ACHT01000499:13262-13636) putative DNA-binding protein - XcampmN\_010100015882 (ACHT01000499:12555-13175) transcriptional regulator, TetR family protein COG1309 Transcriptional regulator |
| ACHT01000106:1752-1812 | ttcgtcgtcccaaacatgccttcggtgaggcgatgcgaatcggagtgttccacgccaaact | 14.1 | - XcampmN\_010100003692 (ACHT01000106:1347-4) type III effector HopG1 |
| ACHT01000215:2118-2149 | **TTCGC**caggcgatgcaaccgc**TTCGC**ttcagt | 14.05 | - XcampmN\_010100006650 (ACHT01000215:1723-68) HpaF protein COG4886 Leucine-rich repeat (LRR) protein |
| ACHT01000720:8707-8762 | tccgccagcagatccagttg**TTCGC**tcaagccgcccagttcctgcaaggacagaat | 13.94 |  |
| ACHT01000046:1341-1372 | ttcgtcatctcacgcatggt**TTCGC**gctgact | 13.84 |  |
| ACHT01000484:3337-3367 | **TTCGC**atgcgcctccagcatttcgggcgcct | 13.51 |  |
| ACHT01000215:17965-18025 | **TTCGC**caacgtattccgatg**TTCGC**ttctgaaaagttctggccagcgcagacgcgtagcgt | 13.48 | - XcampmN\_010100006740 (ACHT01000215:18071-18526) HrpB1 - XcampmN\_010100006745 (ACHT01000215:18635-18952) HrpB2 |
| ACHT01000698:1271-1334 | **TTCGC**attattgcctagatc**TTCGC**gcaacgaaaccgagttcttcgattagcgcgatctaatat | 13.45 | - XcampmN\_010100022328 (ACHT01000698:1699-2457) avirulence protein AvrRxv |
| ACHT01000017:5652-5688 | tgcgccggcatcaccaccta**TTCGC**cgctggcacact | 13.43 | - XcampmN\_010100000265 (ACHT01000017:5178-6236) alcohol dehydrogenase COG1064 Zn-dependent alcohol dehydrogenases |
| ACHT01000484:12205-12243 | **TTCGC**cgccgtagccgccggtgcgcccggtaaacaacgt | 13.41 |  |
| ACHT01000500:19785-19815 | ttccccattgttgccgcaga**TTCGC**gacagt | 13.4 | - XcampmN\_010100016027 (ACHT01000500:20036-17337) ATP-dependent RNA helicase COG1643 HrpA-like helicases |
| ACHT01000039:6659-6706 | **TTCGC**ttgcgacgccagcgcttccgggccgcgcgccgccccgcattgt | 13.36 | - XcampmN\_010100000892 (ACHT01000039:6722-8107) putative penicillin amidase (fragment) COG2366 Protein related to penicillin acylase |
| ACHT01000074:10932-10965 | **TTCGC**gatttcggtcgcttt**TTCGC**tcacagact | 13.33 |  |
| ACHT01000175:9348-9394 | **TTCGC**cggcgtggacccaatttcggttggcgagatccaacgcatcat | 13.21 | - XcampmN\_010100005258 (ACHT01000175:8862-9581) ABC transporter ATP-binding protein COG1137 ABC-type (unclassified) transport system, ATPase component - XcampmN\_010100005263 (ACHT01000175:9622-11061) RNA polymerase factor sigma-54 COG1508 DNA-directed RNA polymerase specialized sigma subunit, sigma54 homolog |
| ACHT01000069:6496-6556 | tacgcccgtggcgctaccga**TTCGC**attgggcaccggccagaccgcagcgctcactatagt | 13.15 | - XcampmN\_010100002069 (ACHT01000069:6369-5857) hypothetical protein |
| ACHT01000564:3100-3161 | ttctctttccaagcgaccac**TTCGC**gcagctgcaacgccacgaacgcgcagcgcaatactgt | 13.15 | - XcampmN\_010100018808 (ACHT01000564:3261-7427) hypothetical protein |
| ACHT01000215:11412-11473 | **TTCGC**gcatgaccatacagc**TTCGC**gtactgaccggaatccatgccggcgcacgattagatt | 13.06 | - XcampmN\_010100006690 (ACHT01000215:10517-10275) HrpD6 protein - XcampmN\_010100006695 (ACHT01000215:11466-10528) HrpD5 protein - XcampmN\_010100006700 (ACHT01000215:12107-11463) HpaA protein |
| ACHT01000695:1633-1675 | ttcggatgcggaataatccc**TTCGC**aatatatgtgtccagaat | 12.97 | - XcampmN\_010100022183 (ACHT01000695:757-1) hypothetical protein |
| ACHT01000525:1766-1811 | **TTCGC**cagctgagctatttcgtcgcactggccgacaccggcagctt | 12.95 | - XcampmN\_010100016884 (ACHT01000525:1744-2643) LysR family transcriptional regulator COG0583 Transcriptional regulator - XcampmN\_010100016889 (ACHT01000525:2733-4421) ABC transporter permease and ATP-binding protein COG2274 ABC-type bacteriocin/lantibiotic exporters, contain an N-terminal double-glycine peptidase domain |
| ACHT01000085:1696-1738 | **TTCGC**catcgggaaacacgtcttcggccaccgctgcgtaagtt | 12.69 |  |
| ACHT01000063:1338-1367 | ttcgttttctgcccaaatt**TTCGC**cagtct | 12.67 | - XcampmN\_010100001839 (ACHT01000063:1298-252) rod shape-determining protein MreB COG1077 Actin-like ATPase involved in cell morphogenesis |
| ACHT01000213:2439-2495 | **TTCGC**tcatgcggataggtg**TTCGC**cagccgggaatccgcactgccgctgatagcgt | 12.64 | - XcampmN\_010100006593 (ACHT01000213:2555-3697) hypothetical protein COG2453 Predicted protein-tyrosine phosphatase |
| ACHT01000524:3055-3086 | ttcgtcgccctgcccggcggcttcggcaccat | 12.6 | - XcampmN\_010100016864 (ACHT01000524:2755-3348) hypothetical protein COG1611 Predicted Rossmann fold nucleotide-binding protein - XcampmN\_010100016869 (ACHT01000524:3569-4207) thymidylate synthase COG1739 Uncharacterized conserved protein |
| ACHT01000443:4064-4097 | tgcgcctgcgcgacaagcgc**TTCGC**cgccacgtt | 12.59 | - XcampmN\_010100013928 (ACHT01000443:3262-2576) hypothetical protein COG5587 Uncharacterized conserved protein - sbcB (ACHT01000443:4704-3259) exonuclease I COG2925 Exonuclease I |
| ACHT01000245:19298-19362 | ttcgtcgacgcatccacagcctcgctttccagccccgccaagctacaggtctgcagcggtaacgt | 12.56 |  |
| ACHT01000186:12456-12494 | tgcgccagagcaggcagtacctcgcgaaaccgctaaaat | 12.55 | - XcampmN\_010100005563 (ACHT01000186:12448-11468) 5'-nucleotidase, lipoprotein e(P4) family COG2503 Predicted secreted acid phosphatase |
| ACHT01000014:77-138 | **TTCGC**caggggacgcgtaactgcgccaacgaacaaaagtcccggcttgcaagtcgttatcct | 12.49 |  |
| ACHT01000479:19879-19910 | ttcggcacgccacgcggatc**TTCGC**ccagata | 12.47 |  |
| ACHT01000045:41273-41304 | ttcgtgctgcgcatgcgccat**TTCGC**caatca | 12.46 | - XcampmN\_010100001352 (ACHT01000045:40714-40448) ISxcd1 transposase COG2801 Transposase and inactivated derivatives - XcampmN\_010100001347 (ACHT01000045:40427-39627) ISxcd1 transposase COG2801 Transposase and inactivated derivatives |
| ACHT01000041:7212-7255 | **TTCGC**aaacgcagccattcg**TTCGC**attaccgccgtcgcagcga | 12.43 | - XcampmN\_010100000952 (ACHT01000041:7209-8357) xylose repressor-like protein COG1940 Transcriptional regulator/sugar kinase |
| ACHT01000560:7491-7544 | ttcggcgtcgtaacgcaccgc**TTCGC**cgagccgcgcatcggcgcggtacagagt | 12.41 |  |
| ACHT01000138:3737-3769 | **TTCGC**attgccagcgagggcttccccgtatacg | 12.38 | - XcampmN\_010100004376 (ACHT01000138:3220-4998) gamma-glutamyltranspeptidase COG0405 Gamma-glutamyltransferase |
| ACHT01000538:35047-35075 | ttcgtcggcatggacgac**TTCGC**caaact | 12.38 | - metG (ACHT01000538:33298-35376) methionyl-tRNA synthetase COG0143 Methionyl-tRNA synthetase - XcampmN\_010100017636 (ACHT01000538:35743-36333) hypothetical protein COG0560 Phosphoserine phosphatase |
| ACHT01000530:6209-6268 | **TTCGC**cgaccagcgccagcac**TTCGC**cggcgcgcaattcgaaatccagatgccgcaccgt | 12.37 |  |
| ACHT01000501:15436-15496 | **TTCGC**cattgatgacagaatttcacataccggcaatgtgcttgtttgctcgcaattaccat | 12.35 | - XcampmN\_010100016137 (ACHT01000501:15534-16175) hypothetical protein |
| ACHT01000093:23096-23126 | ttcgtgttcggcaatggtttttcggcaggat | 12.3 | - XcampmN\_010100003202 (ACHT01000093:23335-23937) hypothetical protein |
| ACHT01000171:8590-8654 | ttctgcagcgcagcggccac**TTCGC**gctgcgaggcaaaaccggacggctggaacttgtccagatt | 12.28 |  |
| ACHT01000424:6525-6556 | tgcgccagcgcggcggccac**TTCGC**tcaaggt | 12.21 |  |
| ACHT01000260:12878-12930 | atcgccttggaaatcaaaat**TTCGC**ctgagtagccctaaccctcagccagact | 12.21 | - XcampmN\_010100008755 (ACHT01000260:12854-12639) hypothetical protein |
| ACHT01000245:37876-37911 | **TTCGC**cgccatagacggcatt**TTCGC**tgcgcatttt | 12.16 |  |
| ACHT01000665:3948-4020 | tacgcagccgcgcacagtgc**TTCGC**gtagcgccatcgccgcctcttgcagcgcccgtttcggtcggctacatt | 12.15 | - XcampmN\_010100021183 (ACHT01000665:4051-4524) MarR family transcriptional regulator COG1846 Transcriptional regulators - XcampmN\_010100021188 (ACHT01000665:4570-5397) p-hydroxycinnamoyl CoA hydratase/lyase COG1024 Enoyl-CoA hydratase/carnithine racemase |
| ACHT01000318:3509-3539 | ttcgtcaatatcggcgcggtatcgctagact | 12.06 |  |
| ACHT01000248:1009-1059 | atcgccatcaccaacgcatc**TTCGC**gcccggtgctggaggggtagtagcgt | 11.98 |  |
| ACHT01000161:3508-3538 | **TTCGC**tcgcagttgggtttg**TTCGC**caacct | 11.98 | - XcampmN\_010100004861 (ACHT01000161:3225-4298) 3-isopropylmalate dehydrogenase COG0473 Isocitrate/isopropylmalate dehydrogenase |
| ACHT01000521:2378-2421 | **TTCGC**ctggaaagaagcaacttcacggtgctcaacaagcaactt | 11.95 | - XcampmN\_010100016814 (ACHT01000521:2323-2616) putative secreted protein |
| ACHT01000300:5340-5369 | ttcgtcggccagccgcgcttgcgccagctt | 11.95 |  |
| ACHT01000043:10217-10286 | atcgcctgggtcgctgccgc**TTCGC**ctccacgccgtttctcaagagcgccggcaagccgtggcatagcct | 11.94 | - XcampmN\_010100001107 (ACHT01000043:9991-11760) putative siderophore biosynthesis protein COG4264 Siderophore synthetase component |
| ACHT01000142:2198-2275 | ttcggcaccaccgatgccac**TTCGC**atggtttccgcaaggccggtctggtcaacgggcagatcggcggtttctacaat | 11.93 | - XcampmN\_010100004586 (ACHT01000142:1385-2675) sugar transporter COG0477 Permeases of the major facilitator superfamily |
| ACHT01000355:8611-8641 | **TTCGC**tgcagcattcaggct**TTCGC**cagcgc | 11.91 | - glk (ACHT01000355:7772-8709) glucokinase COG0837 Glucokinase |
| ACHT01000108:476-506 | **TTCGC**cgcgcagcatgttattcacataagct | 11.88 |  |
| ACHT01000601:3170-3201 | ttcgtcggcgccgcgcggcag**TTCGC**tgaact | 11.85 |  |
| ACHT01000242:15941-15970 | ttcgttggcgatgcgattgttcggcaacat | 11.84 | - XcampmN\_010100007620 (ACHT01000242:16230-16535) F0F1 ATP synthase subunit C COG0636 F0F1-type ATP synthase, subunit c/Archaeal/vacuolar-type H -ATPase, subunit K - XcampmN\_010100007615 (ACHT01000242:15357-16157) F0F1 ATP synthase subunit A COG0356 F0F1-type ATP synthase, subunit a - XcampmN\_010100007625 (ACHT01000242:16650-17120) F0F1 ATP synthase subunit B COG0711 F0F1-type ATP synthase, subunit b |
| ACHT01000202:8321-8442 | **TTCGC**atgcggccgcatgca**TTCGC**gccgggatctgcatgcgcgctagtgcctgcgcgatgctgccaggcccgcgcggcgcgacgcgctcactggatcgcgcatgcgtctcaaggccacagt | 11.79 | - XcampmN\_010100006263 (ACHT01000202:8235-7384) 2-keto-3-deoxygluconate permease |
| ACHT01000083:6952-6982 | tacgccggcgcacacggtct**TTCGC**tgccct | 11.73 | - XcampmN\_010100002597 (ACHT01000083:6584-6105) hypothetical protein |
| ACHT01000671:738-769 | **TTCGC**ggtggcgtagctgtggcttcggtacat | 11.71 | - XcampmN\_010100021503 (ACHT01000671:752-1) TonB-dependent receptor COG1629 Outer membrane receptor proteins, mostly Fe transport |
| ACHT01000215:15677-15733 | **TTCGC**aaaagcgcaccggat**TTCGC**atcacccttgtcgatgcggctgtcgccacagt | 11.68 | - XcampmN\_010100006725 (ACHT01000215:14853-14215) HpaC protein - XcampmN\_010100006730 (ACHT01000215:16775-14853) HrcV protein COG4789 Type III secretory pathway, component EscV |
| ACHT01000296:13068-13115 | tgcgcttgcgcggacatcgc**TTCGC**agggtcgcgaccgcgcgcaacct | 11.67 | - XcampmN\_010100009656 (ACHT01000296:12567-11896) hypothetical protein COG0110 Acetyltransferase (isoleucine patch superfamily) - XcampmN\_010100009661 (ACHT01000296:13142-12564) NDP-hexose isomerase |
| ACHT01000598:5639-5677 | **TTCGC**ttttcgggcaatggtttcgtgtagtcgtcacgat | 11.67 | - XcampmN\_010100019438 (ACHT01000598:5884-6630) alpha/beta fold family hydrolase COG0596 Predicted hydrolases or acyltransferases (alpha/beta hydrolase superfamily) |
| ACHT01000099:2925-2966 | **TTCGC**ccaggctcaaagcta**TTCGC**atcagcagcgctatacg | 11.64 | - XcampmN\_010100003347 (ACHT01000099:2865-3245) hypothetical protein - XcampmN\_010100003352 (ACHT01000099:3450-4754) hypothetical protein |
| ACHT01000651:808-842 | **TTCGC**tcccaacgtcatcct**TTCGC**agcgttcagt | 11.64 | - XcampmN\_010100020718 (ACHT01000651:608-1) chlorogenate esterase |
| ACHT01000713:13933-13967 | **TTCGC**gggttcggcagttt**TTCGC**tgcattaccgt | 11.63 | - XcampmN\_010100022643 (ACHT01000713:13721-13416) hypothetical protein - XcampmN\_010100022638 (ACHT01000713:13409-12231) tetratricopeptide repeat protein COG2956 Predicted N-acetylglucosaminyl transferase - ihfB (ACHT01000713:14100-13789) integration host factor subunit beta COG0776 Bacterial nucleoid DNA-binding protein |
| ACHT01000143:1188-1238 | **TTCGC**actagaggcgggttg**TTCGC**gcggtggcggccgcttgtgcgcagat | 11.61 |  |
| ACHT01000644:8320-8350 | **TTCGC**cagccgcaccagcttctcgtccgagt | 11.61 |  |
| ACHT01000085:14979-15010 | **TTCGC**cggcgctgccgccgatgcgtttacctt | 11.59 | - hslU (ACHT01000085:14508-13165) ATP-dependent protease ATP-binding subunit HslU COG1220 ATP-dependent protease HslVU (ClpYQ), ATPase subunit - XcampmN\_010100002747 (ACHT01000085:15184-14633) ATP-dependent protease peptidase subunit COG5405 ATP-dependent protease HslVU (ClpYQ), peptidase subunit |
| ACHT01000089:32616-32689 | tgcgcagccgcatccgtgac**TTCGC**ggccgtgacagcgcaacccctggtgtgcgttgccgcgatctcgtatttt | 11.56 | - XcampmN\_010100002952 (ACHT01000089:32583-31204) D-alanine/D-serine/glycine permease COG1113 Gamma-aminobutyrate permease and related permeases |
| ACHT01000199:8294-8325 | **TTCGC**tggatgcgccaggt**TTCGC**cgcatgct | 11.5 |  |
| ACHT01000252:18538-18589 | ttcttcagcagcaccagcga**TTCGC**gcgcagtgcgtcgcgccagcgcatcat | 11.5 |  |
| ACHT01000719:1814-1860 | ttcgtgcatcagcgccaacac**TTCGC**tgcgcagatcctgggcaatct | 11.47 |  |
| ACHT01000112:26574-26607 | ttctttcgagagcacgtggc**TTCGC**acgtaccct | 11.45 |  |
| ACHT01000091:5157-5202 | **TTCGC**aagcggcgacatcgctgcgccttgtcttttcggtgcaatgt | 11.45 | - XcampmN\_010100003072 (ACHT01000091:5585-8278) TonB-dependent receptor COG1629 Outer membrane receptor proteins, mostly Fe transport |
| ACHT01000252:14532-14562 | tgcaccagcctaccgaggacttcgtcaaact | 11.44 | - XcampmN\_010100008410 (ACHT01000252:15109-14006) prophage Lp2 protein 6 COG4748 Uncharacterized conserved protein |
| ACHT01000142:1940-1970 | ttcgtgatcggcgcgggtttttctgcagcgt | 11.44 | - XcampmN\_010100004586 (ACHT01000142:1385-2675) sugar transporter COG0477 Permeases of the major facilitator superfamily |
| ACHT01000678:11871-11920 | atcgtcagcgacgccggcactccgctggtcagcgacccgggcttcaaact | 11.44 | - XcampmN\_010100021748 (ACHT01000678:11352-10627) hypothetical protein - XcampmN\_010100021753 (ACHT01000678:12163-11336) hypothetical protein COG0313 Predicted methyltransferases |
| ACHT01000093:12185-12214 | **TTCGC**cagccgcgctggaaatccggtagct | 11.43 |  |
| ACHT01000190:2537-2618 | ttcgtaacgcagggcagagg**TTCGC**aattactgaattttggctatcggcaagtgaaccaatcgcgttcgaatcggtcacact | 11.42 | - XcampmN\_010100005708 (ACHT01000190:2530-1511) methanol dehydrogenase regulatory protein COG0714 MoxR-like ATPases |
| ACHT01000242:27033-27062 | **TTCGC**tgcgcacgcaagacatcgccaagtt | 11.38 |  |
| ACHT01000447:1956-2007 | **TTCGC**cggcgcgcgaggc**TTCGC**cggtatcgcggttatgcgtctcccaacgt | 11.37 |  |
| ACHT01000409:431-461 | **TTCGC**tagattgcgccgcgctgcgctagaat | 11.36 | - XcampmN\_010100012390 (ACHT01000409:1-458) hypothetical protein |
| ACHT01000564:14834-14871 | atcgccggcgcgctgtgcc**TTCGC**gggcaggacacact | 11.36 | - XcampmN\_010100018833 (ACHT01000564:14039-15157) hypothetical protein COG3146 Uncharacterized protein conserved in bacteria - aat (ACHT01000564:15411-16160) leucyl/phenylalanyl-tRNA--protein transferase COG2360 Leu/Phe-tRNA-protein transferase |
| ACHT01000095:560-620 | ttcgttcttgcggcgtccgg**TTCGC**tcgctttgcacgcatgcgggcagtgcttcgtagagt | 11.35 | - XcampmN\_010100003247 (ACHT01000095:314-733) IS1404 transposase COG2801 Transposase and inactivated derivatives |
| ACHT01000676:3411-3442 | ttcggcttcggcacgcagctt**TTCGC**cgcact | 11.34 |  |
| ACHT01000175:15706-15745 | ttggccagcgcacgcaggcg**TTCGC**ggtagtcgcgcagat | 11.32 |  |
| ACHT01000542:5632-5674 | atcgcatccgcacccagatgttcggcctgatcgccatgaccct | 11.32 | - XcampmN\_010100018026 (ACHT01000542:5681-4095) periplasmic serine protease MucD COG0265 Trypsin-like serine proteases, typically periplasmic, contain C-terminal PDZ domain |
| ACHT01000480:9047-9105 | atcgccagcgaataggcctc**TTCGC**ccgtggagcagcccagcacccagatgcgcaggct | 11.29 |  |
| ACHT01000388:5994-6082 | ttcgtatcagtcaccacgca**TTCGC**gcggcatgccaacagcgcggcattcggtggtttactaaagcagcgatgtcacgcacatcaaaat | 11.23 | - XcampmN\_010100011870 (ACHT01000388:6620-7867) hypothetical protein |
| ACHT01000575:5961-5998 | **TTCGC**tgagcaaatccagcgcttcggtcaacgcagtct | 11.22 |  |
| ACHT01000424:7686-7748 | **TTCGC**cgttgttgaacggcg**TTCGC**ccgcgcctgctgctctgggccagcagttgcgctacagt | 11.22 | - XcampmN\_010100013264 (ACHT01000424:7864-9339) anthranilate synthase component I COG0147 Anthranilate/para-aminobenzoate synthases component I |
| ACHT01000198:1224-1294 | ttggccagcacccgcggcgc**TTCGC**cgcgctgtgccagcgagaagatcatccgcgaggcgccatacagatt | 11.2 |  |
| ACHT01000525:10092-10122 | tgcatcattcaaggcacagc**TTCGC**tagcgt | 11.18 |  |
| ACHT01000196:20984-21053 | ttcgtccgcactgcgactcc**TTCGC**agcgccagccattaggacgaatgccatgaacgccgcgcccaccgt | 11.17 | - XcampmN\_010100006073 (ACHT01000196:21003-19126) thiamine biosynthesis protein ThiC COG0422 Thiamine biosynthesis protein ThiC |
| ACHT01000436:4852-4888 | ttcttcctgcagtgcagcac**TTCGC**accgcgctacat | 11.17 | - XcampmN\_010100013633 (ACHT01000436:4771-2588) XopX effector protein |
| ACHT01000093:24519-24580 | **TTCGC**catcggtcacgcttattcgggcaatgccgcgcgtgcgtgattgggcaacggaacaat | 11.16 | - XcampmN\_010100003207 (ACHT01000093:24837-25385) hypothetical protein |
| ACHT01000041:8439-8478 | tgcgcaccgcaggcagcgt**TTCGC**atacaggtggcatatt | 11.15 | - XcampmN\_010100000962 (ACHT01000041:8628-10694) avirulence protein COG0584 Glycerophosphoryl diester phosphodiesterase |
| ACHT01000200:1539-1590 | **TTCGC**ctgggtgggccggaattcggcttgagccaaagccagatcggcatgat | 11.12 | - XcampmN\_010100006163 (ACHT01000200:770-2050) MFS transporter COG0477 Permeases of the major facilitator superfamily |
| ACHT01000027:13446-13477 | ttggccagcagctcaagctcttcggccaccat | 11.12 |  |
| ACHT01000405:6301-6335 | **TTCGC**ccggcagatccaaccc**TTCGC**cgaaagaat | 11.07 |  |
| ACHT01000093:22895-22924 | **TTCGC**ccacgcttgcacagcttcgttcatt | 11.03 |  |
| ACHT01000059:4134-4164 | atcgtctgtgcgcaatcgacttcgtcacagt | 11.03 | - XcampmN\_010100001697 (ACHT01000059:4800-5017) hypothetical protein |
| ACHT01000212:2968-2998 | **TTCGC**gaacacgggcacgacgtcgccagtgt | 11.01 | - XcampmN\_010100006548 (ACHT01000212:2679-1774) peptide-aspartate beta-dioxygenase COG3555 Aspartyl/asparaginyl beta-hydroxylase and related dioxygenases - XcampmN\_010100006558 (ACHT01000212:3812-2928) biotin synthesis protein COG0500 SAM-dependent methyltransferases |
| ACHT01000626:9592-9638 | accgccagcgcacccagcgt**TTCGC**acagcaggtcctggatcaggtt | 11.01 |  |
| ACHT01000699:1159-1190 | **TTCGC**catcgttggccaccacttccttgtact | 11 |  |
| ACHT01000191:12210-12240 | ttcgtttcaggccccgtcagtacgccagcgt | 11 | - XcampmN\_010100005778 (ACHT01000191:12164-9993) guanosine-3',5'-bis(diphosphate) 3'-pyrophosphohydrolase COG0317 Guanosine polyphosphate pyrophosphohydrolases/synthetases |
| ACHT01000099:5039-5083 | atcgtcatcgacggcggcaattcgtactacaagga**TTCGC**aacgt | 10.98 | - XcampmN\_010100003357 (ACHT01000099:4775-5716) 6-phosphogluconate dehydrogenase-like protein COG1023 Predicted 6-phosphogluconate dehydrogenase |
| ACHT01000530:9766-9824 | atcgcctgcagatcggctgc**TTCGC**gcttggcgttggccactgcggccggtgccagttt | 10.97 |  |
| ACHT01000064:113-150 | **TTCGC**tgtcgatcagcgcttcggggatcgccgcacaat | 10.91 |  |
| ACHT01000409:29752-29786 | atcgcatgccgctgcaggct**TTCGC**cgtacaccat | 10.9 | - XcampmN\_010100012560 (ACHT01000409:30477-30647) hypothetical protein |
| ACHT01000138:19038-19090 | **TTCGC**cggcgaaacccagatcttcatcctgcctggctaccgcatccgcagcgt | 10.9 | - queA (ACHT01000138:18210-19280) S-adenosylmethionine:tRNA ribosyltransferase-isomerase COG0809 S-adenosylmethionine:tRNA-ribosyltransferase-isomerase (queuine synthetase) - tgt (ACHT01000138:19355-20500) queuine tRNA-ribosyltransferase COG0343 Queuine/archaeosine tRNA-ribosyltransferase |
| ACHT01000662:3883-3916 | **TTCGC**atggtaggcggttag**TTCGC**tttttaagt | 10.87 | - XcampmN\_010100020968 (ACHT01000662:4175-4750) hypothetical protein COG0778 Nitroreductase - XcampmN\_010100020973 (ACHT01000662:4747-5733) exodeoxyribonuclease IX COG0258 5'-3' exonuclease (including N-terminal domain of PolI) |
| ACHT01000222:562-595 | **TTCGC**catcttctcgaccgcttcggcgctaccgg | 10.87 | - XcampmN\_010100006870 (ACHT01000222:546-962) hypothetical protein - XcampmN\_010100006875 (ACHT01000222:1254-1436) hypothetical protein |
| ACHT01000485:249-307 | ttcggtcgaggcatcacttt**TTCGC**tgctcgccagggcatggattcgaaatcgtatttt | 10.86 | - XcampmN\_010100015167 (ACHT01000485:699-1052) hypothetical protein |
| ACHT01000242:16689-16723 | **TTCGC**cggcctgatctggatcgtcgcgaccaaaat | 10.84 | - XcampmN\_010100007630 (ACHT01000242:17124-17651) F0F1 ATP synthase subunit delta COG0712 F0F1-type ATP synthase, delta subunit (mitochondrial oligomycin sensitivity protein) - XcampmN\_010100007625 (ACHT01000242:16650-17120) F0F1 ATP synthase subunit B COG0711 F0F1-type ATP synthase, subunit b |
| ACHT01000712:9666-9717 | **TTCGC**gcttgaccgggaatttcggcacggcggtgtaggcgggcgcgcacaat | 10.84 |  |
| ACHT01000500:25266-25309 | atcgcgctcagcggcagcac**TTCGC**cccacggcgcgtccacggt | 10.84 |  |
| ACHT01000490:10173-10207 | **TTCGC**tgctgcgagaagggt**TTCGC**gtatgacgct | 10.81 | - XcampmN\_010100015352 (ACHT01000490:10500-11777) transglycosylase COG2951 Membrane-bound lytic murein transglycosylase B |
| ACHT01000436:1989-2030 | **TTCGC**cggcattggccacgat**TTCGC**gcagcggggcttccat | 10.8 |  |
| ACHT01000690:7348-7404 | ttggcctgcagcgcggtgtcttcggtctgcgcggccagggcctgcgcccagtacatt | 10.79 |  |
| ACHT01000461:6880-6915 | ttcgcc**TTCGC**ggctaatgcc**TTCGC**cgtacaggat | 10.79 |  |
| ACHT01000334:11558-11589 | **TTCGC**cattgccacaatgagtccgctatgaat | 10.79 | - XcampmN\_010100010634 (ACHT01000334:11584-12000) hypothetical protein COG4244 Predicted membrane protein - XcampmN\_010100010639 (ACHT01000334:11997-12734) L-sorbosone dehydrogenase COG2133 Glucose/sorbosone dehydrogenases |
| ACHT01000417:3857-3900 | ttcgtcggcggcggcgtgacc**TTCGC**ggccaagccgcgcagctt | 10.78 | - rplW (ACHT01000417:4231-4530) 50S ribosomal protein L23 COG0089 Ribosomal protein L23 - rplD (ACHT01000417:3629-4234) 50S ribosomal protein L4 COG0088 Ribosomal protein L4 - rplB (ACHT01000417:4541-5368) 50S ribosomal protein L2 COG0090 Ribosomal protein L2 |
| ACHT01000491:5903-5961 | **TTCGC**cagcggcgtcacaactgcgttgatcggccccagcggcgccggcaaatccaccgt | 10.78 | - XcampmN\_010100015392 (ACHT01000491:5834-6571) ABC transporter ATP-binding protein COG1125 ABC-type proline/glycine betaine transport systems, ATPase components - XcampmN\_010100015397 (ACHT01000491:6568-8040) ABC transporter permease and substrate-binding protein COG1732 Periplasmic glycine betaine/choline-binding (lipo)protein of an ABC-type transport system (osmoprotectant binding protein) |
| ACHT01000212:6400-6466 | atcgccatcacggtcacttc**TTCGC**cgaacagcaacggctggatcggtgtgtcagaagccatcaatt | 10.77 |  |
| ACHT01000093:13931-13968 | atcgcttcggcggccagcacttcgggcgcgggcaggct | 10.76 |  |
| ACHT01000713:36924-36955 | ttcggcacgcggatcgtcttc**TTCGC**catcct | 10.76 | - XcampmN\_010100022738 (ACHT01000713:36187-35888) YciI-like protein COG2350 Uncharacterized protein conserved in bacteria |
| ACHT01000317:2391-2448 | ttcttctgcggatccttggt**TTCGC**cggcggaggtcgagaccgcgtcgaagccaatgt | 10.74 | - XcampmN\_010100010297 (ACHT01000317:1478-891) hypothetical protein COG0494 NTP pyrophosphohydrolases including oxidative damage repair enzymes |
| ACHT01000673:4320-4372 | **TTCGC**gcaggtactccagata**TTCGC**gcacatgccgctcgcgtgcggcaacgt | 10.74 |  |
| ACHT01000016:4331-4364 | ttcgtttgctgcggtggcgcttcgtccctaaaaa | 10.72 | - XcampmN\_010100000225 (ACHT01000016:4267-3416) putative secreted protein |
| ACHT01000186:1433-1466 | gtcgccaccgacacctgcgc**TTCGC**gcgcaacgt | 10.72 | - XcampmN\_010100005533 (ACHT01000186:979-8) putative secreted protein |
| ACHT01000431:3095-3124 | ctcgcccgacaagccggccttcgtcaccgt | 10.72 | - XcampmN\_010100013573 (ACHT01000431:2861-2553) hypothetical protein - XcampmN\_010100013563 (ACHT01000431:2475-1108) acetyl-CoA carboxylase biotin carboxylase subunit COG0439 Biotin carboxylase - XcampmN\_010100013578 (ACHT01000431:3423-2938) acetyl-CoA carboxylase biotin carboxyl carrier protein subunit COG0511 Biotin carboxyl carrier protein |
| ACHT01000659:973-1003 | ttcgtccccggcgccgcgcgctcggtacaga | 10.7 | - rbn (ACHT01000659:1249-1) ribonuclease BN/unknown domain fusion protein COG1295 Predicted membrane protein |
| ACHT01000409:17532-17562 | atcgcgacccgaagaacttcttccccaactt | 10.69 | - XcampmN\_010100012490 (ACHT01000409:16671-15346) ABC transporter permease COG0577 ABC-type antimicrobial peptide transport system, permease component - XcampmN\_010100012495 (ACHT01000409:17839-16679) ABC transporter permease COG0577 ABC-type antimicrobial peptide transport system, permease component |
| ACHT01000112:3409-3455 | ttctccagccagcgcgcagcttcggcgaagcactcctggcgtataca | 10.69 | - XcampmN\_010100003867 (ACHT01000112:2583-2386) hypothetical protein |
| ACHT01000253:7024-7065 | ctcgcagcgcagcaagcctttcggccgccggctcggtacagt | 10.65 | - XcampmN\_010100008460 (ACHT01000253:6774-5437) xylose isomerase COG2115 Xylose isomerase |
| ACHT01000015:5795-5825 | ttgcccggcgagcccaacaa**TTCGC**cagtat | 10.62 | - XcampmN\_010100000210 (ACHT01000015:4361-6328) outer protein F2 |
| ACHT01000713:16972-17007 | tccgccgccgcggcaaggtcttcgtgatctgcaagt | 10.6 | - XcampmN\_010100022668 (ACHT01000713:17281-17622) hypothetical protein - rpmJ (ACHT01000713:16914-17039) 50S ribosomal protein L36 COG0257 Ribosomal protein L36 - XcampmN\_010100022673 (ACHT01000713:17858-18892) hypothetical protein COG2957 Peptidylarginine deiminase and related enzymes |
| ACHT01000500:4402-4473 | ttcggcctgcgcgacgtcgg**TTCGC**cccccgccttcggcaccgcaaggggatcgctacgatggacccacagt | 10.59 | - XcampmN\_010100015942 (ACHT01000500:4384-3164) hypothetical protein |
| ACHT01000663:58-90 | **TTCGC**tttgcgcgcaccaaattcgttttacgat | 10.59 | - XcampmN\_010100021063 (ACHT01000663:1-100) hypothetical protein |
| ACHT01000697:1015-1086 | ttcgagacgcgatcaagatc**TTCGC**agaccatgcgaggcaacgcgcatcctcgtccgcgctttgcacacact | 10.57 |  |
| ACHT01000495:20231-20261 | atcgctcccgctgccgacac**TTCGC**catcga | 10.56 | - XcampmN\_010100015582 (ACHT01000495:20162-19386) uroporphyrinogen-III synthase COG1587 Uroporphyrinogen-III synthase |
| ACHT01000428:7910-8004 | ttcgtcggcggcggcgatcac**TTCGC**catcgcgcatcgaaccaccgggctgaatcactgccttgatgccggccgctgccgccgcgtcgataccat | 10.56 |  |
| ACHT01000662:17760-17812 | **TTCGC**cgtgcagttggtc**TTCGC**attcgatgcgtgagtgcatggtgacaacat | 10.55 |  |
| ACHT01000643:2197-2236 | tccgccccttcagcaattct**TTCGC**tgcgttctgtatcct | 10.54 |  |
| ACHT01000245:19388-19419 | **TTCGC**atcgtaaatcccgggc**TTCGC**gaatat | 10.53 |  |
| ACHT01000644:23803-23884 | **TTCGC**tcggtgcgccatctctgcgcaccggtagcaaaccgccatctcgtcgaagaaattaagtcatcgaaagtaagtaaatt | 10.53 | - XcampmN\_010100020278 (ACHT01000644:24106-25140) hypothetical protein COG0584 Glycerophosphoryl diester phosphodiesterase |
| ACHT01000691:1515-1559 | ttcgtccagcacgcagat**TTCGC**aggcgtgcagcgacaccacctt | 10.52 | - XcampmN\_010100022048 (ACHT01000691:860-519) thioredoxin COG0526 Thiol-disulfide isomerase and thioredoxins |
| ACHT01000644:15490-15522 | **TTCGC**tgagcagcagcgc**TTCGC**ggtccatgct | 10.52 | - XcampmN\_010100020238 (ACHT01000644:14897-14307) putative secreted protein COG0526 Thiol-disulfide isomerase and thioredoxins |
| ACHT01000257:18106-18134 | ttctccggtgtgctcggta**TTCGC**cagat | 10.51 | - XcampmN\_010100008680 (ACHT01000257:17620-18690) RND efflux membrane fusion protein COG0845 Membrane-fusion protein - XcampmN\_010100008685 (ACHT01000257:18687-21812) acriflavin resistance protein COG0841 Cation/multidrug efflux pump |
| ACHT01000550:13750-13798 | **TTCGC**cggacagccccac**TTCGC**cgaacgcgatggtcttttcggacaat | 10.49 |  |
| ACHT01000325:10184-10217 | ttcgtctgtctggcgtgtcg**TTCGC**ctacaacct | 10.49 | - XcampmN\_010100010497 (ACHT01000325:9091-10374) metabolite:H symporter family protein COG0477 Permeases of the major facilitator superfamily |
| ACHT01000329:2314-2343 | **TTCGC**tgccagacccggtgttcgacacggt | 10.48 | - XcampmN\_010100010544 (ACHT01000329:2288-4063) hypothetical protein COG2203 FOG: GAF domain |
| ACHT01000662:7778-7812 | gtcgccgagcagctggctcttcgtgttgttaactt | 10.46 |  |
| ACHT01000742:6278-6316 | **TTCGC**cgcccagcaagaccttggcgcccttttccacgct | 10.46 |  |
| ACHT01000435:747-778 | **TTCGC**cagccgtggccaccacttccacacctt | 10.46 |  |
| ACHT01000487:12681-12714 | **TTCGC**gggcgcagctggcgatgcgggtttagata | 10.46 |  |
| ACHT01000541:12908-12978 | ttcgtcggcaaacgcgccactgcgctgcgcggcctgcgcgcgttccaccgaggcaatggcgaatgcatcct | 10.44 | - XcampmN\_010100017876 (ACHT01000541:12229-9392) serine protease COG1404 Subtilisin-like serine proteases |
| ACHT01000577:9028-9061 | ttcgtcgtccaggcgtgtcgtcgctacggagatt | 10.43 | - XcampmN\_010100019113 (ACHT01000577:8362-7907) hypothetical protein |
| ACHT01000191:23770-23826 | **TTCGC**gcgggccatcccaggc**TTCGC**gctcggcgtcgcgctcgcgacagaacaaaat | 10.4 |  |
| ACHT01000567:3501-3550 | **TTCGC**ccagtcgcacgat**TTCGC**cgcattcttcggcaatgggaataaatt | 10.39 |  |
| ACHT01000411:5364-5399 | **TTCGC**cgcggttacgcggcca**TTCGC**cagccagtgt | 10.38 |  |
| ACHT01000527:6127-6158 | ttcgacttgatcgccgcgtattcggcgatatt | 10.37 |  |
| ACHT01000196:20393-20429 | atcgcgcgcccgcgcgcgatttcgtcgcgcacaaact | 10.37 |  |
| ACHT01000045:19282-19322 | gtcgcatccacatcgaagac**TTCGC**gcatcgccagcatatt | 10.37 | - XcampmN\_010100001242 (ACHT01000045:18774-18652) hypothetical protein |
| ACHT01000245:13284-13320 | ttcgtcgtagccgacgccactgcgctgcctttccaat | 10.37 | - XcampmN\_010100007895 (ACHT01000245:13086-14741) WsaE COG0500 SAM-dependent methyltransferases |
| ACHT01000045:48206-48248 | tccgccagggcgcgcagcgcttcggcctggtggtggacacctt | 10.37 | - XcampmN\_010100001382 (ACHT01000045:47971-45818) chemotaxis protein COG0840 Methyl-accepting chemotaxis protein - XcampmN\_010100001387 (ACHT01000045:49225-48040) chemotaxis histidine protein kinase COG0643 Chemotaxis protein histidine kinase and related kinases |
| ACHT01000461:2122-2157 | **TTCGC**cttgaagcagcacaatcgcgctatgcagact | 10.36 |  |
| ACHT01000484:6656-6704 | **TTCGC**gctggcggcaatcatctcgcccaccgacccggtggcggtatcgt | 10.35 | - XcampmN\_010100015122 (ACHT01000484:6311-7948) Na :H antiporter COG0025 NhaP-type Na /H and K /H antiporters |
| ACHT01000017:5809-5864 | ttcgtctttggaaatcaccac**TTCGC**tggcgcccaggcgcagtgcatcagcacgct | 10.35 | - XcampmN\_010100000255 (ACHT01000017:4866-3793) cellulase COG2730 Endoglucanase |
| ACHT01000648:36565-36598 | ttcgtccatgtcggaggttaatcgcaggcataat | 10.34 |  |
| ACHT01000399:2536-2571 | **TTCGC**atcgtgaccaccgac**TTCGC**ctccgacaagt | 10.32 | - XcampmN\_010100012050 (ACHT01000399:3034-3438) hypothetical protein - XcampmN\_010100012045 (ACHT01000399:1788-2969) hypothetical protein - XcampmN\_010100012055 (ACHT01000399:3441-4115) DNA-3-methyladenine glycosylase COG0122 3-methyladenine DNA glycosylase/8-oxoguanine DNA glycosylase |
| ACHT01000644:2852-2883 | tacgcggccccgcccaggtgttcgtacagaat | 10.32 |  |
| ACHT01000485:576-618 | tgcgcccgagtgcgggatta**TTCGC**acattggtctcttatatt | 10.32 | - XcampmN\_010100015157 (ACHT01000485:181-8) hypothetical protein - XcampmN\_010100015162 (ACHT01000485:656-513) hypothetical protein |
| ACHT01000538:33990-34023 | **TTCGC**gcgatgcgccgtatttcggtttccagatt | 10.32 | - metG (ACHT01000538:33298-35376) methionyl-tRNA synthetase COG0143 Methionyl-tRNA synthetase |
| ACHT01000208:324-375 | **TTCGC**caatccaaacgcagtttcgttccgacgctggacttcatcaccacggt | 10.32 | - XcampmN\_010100006448 (ACHT01000208:1-679) glutaconate CoA transferase subunit B COG2057 Acyl CoA:acetate/3-ketoacid CoA transferase, beta subunit - XcampmN\_010100006453 (ACHT01000208:676-1884) beta-ketoadipyl CoA thiolase COG0183 Acetyl-CoA acetyltransferase |
| ACHT01000491:6788-6818 | tgcgccagcgtgccggtgtattcggcatacg | 10.31 |  |
| ACHT01000747:11548-11577 | ttcacctgcgcgcgtgctg**TTCGC**cagctt | 10.29 | - XcampmN\_010100023612 (ACHT01000747:11612-10332) metabolite transport protein COG0477 Permeases of the major facilitator superfamily |
| ACHT01000508:3939-3968 | **TTCGC**tgctggcgcagcggttgcgccagat | 10.29 | - XcampmN\_010100016192 (ACHT01000508:3466-6594) acriflavin resistance protein COG0841 Cation/multidrug efflux pump |
| ACHT01000667:2197-2253 | **TTCGC**gcacccgcgcaggatc**TTCGC**gcccggccagttccagcggcagcgcgatgtt | 10.27 | - XcampmN\_010100021368 (ACHT01000667:1841-1188) acyl-CoA thioesterase I COG2755 Lysophospholipase L1 and related esterases |
| ACHT01000713:38041-38071 | atcgccgccgaggccggcactgcgccatcca | 10.27 |  |
| ACHT01000426:3421-3476 | **TTCGC**cttcgacaccaacggttgcataggtgaattcagcaacacggacattacatt | 10.26 |  |
| ACHT01000242:4330-4372 | **TTCGC**cccggccgagcgcg**TTCGC**ggtgctgcagatggacatt | 10.24 | - XcampmN\_010100007555 (ACHT01000242:4448-5047) hypothetical protein |
| ACHT01000367:3187-3265 | ttggccagccggcgcccggc**TTCGC**gcatcgccacgatttcggcatgcgcgctgggatcgtggctggcgatattaaagt | 10.24 | - XcampmN\_010100011393 (ACHT01000367:4050-5384) manganese transport protein MntH COG1914 Mn2 and Fe2 transporters of the NRAMP family |
| ACHT01000676:1639-1667 | **TTCGC**cggccgggtagac**TTCGC**tcaaca | 10.24 |  |
| ACHT01000069:4122-4175 | atcgtcattggacgaagctgttcggttctggacaggagcactcggctttacgct | 10.23 | - XcampmN\_010100002049 (ACHT01000069:3982-4533) hypothetical protein COG0346 Lactoylglutathione lyase and related lyases |
| ACHT01000726:10614-10645 | atcgcttgcgcgaccagcgttttcggcaacct | 10.23 | - XcampmN\_010100023210 (ACHT01000726:11141-11383) hypothetical protein COG1671 Uncharacterized protein conserved in bacteria - XcampmN\_010100023215 (ACHT01000726:11454-12002) hypothetical protein COG4681 Uncharacterized protein conserved in bacteria |
| ACHT01000683:16295-16325 | tgcgccttgcccagcgcggcttcggcatctt | 10.22 |  |
| ACHT01000527:1877-1907 | **TTCGC**cggcgggcgcctggtgtcgccaaaag | 10.22 | - XcampmN\_010100017059 (ACHT01000527:2357-2506) hypothetical protein - XcampmN\_010100017054 (ACHT01000527:1052-2086) LysR family transcriptional regulator COG0583 Transcriptional regulator |
| ACHT01000639:1156-1214 | **TTCGC**cagatcggcaacgccatcgccgagcagaccagcgccagcgacagcgagcacaat | 10.22 | - XcampmN\_010100019958 (ACHT01000639:1707-148) putative ATP-binding protein COG4913 Uncharacterized protein conserved in bacteria |
| ACHT01000033:4500-4575 | **TTCGC**cactccaggcagaaatcgcctgatccggatcattgccgtagcgcttacgcagatccggctgcagatacagt | 10.21 |  |
| ACHT01000171:7834-7869 | **TTCGC**ccggcggcgaagtg**TTCGC**ctctgagcagat | 10.2 | - XcampmN\_010100005143 (ACHT01000171:8862-7110) protease IV COG0616 Periplasmic serine proteases (ClpP class) |
| ACHT01000091:1198-1236 | atcgccgacacccgcatggt**TTCGC**cggggcggtacaga | 10.19 |  |
| ACHT01000275:378-415 | tgcgcccacggcccgatggt**TTCGC**cactgatcaccat | 10.18 |  |
| ACHT01000588:5708-5765 | **TTCGC**atgaaaatcaaacgcttcgttgccccggacatgcgcaccgctttccgcatggt | 10.18 | - XcampmN\_010100019283 (ACHT01000588:5713-7172) flagellar biosynthesis regulator FlhF COG1419 Flagellar GTP-binding protein |
| ACHT01000242:40947-40999 | **TTCGC**ctgcgcatccgctggtttccatgcccatccctgccaagacgccagcct | 10.18 |  |
| ACHT01000111:9707-9741 | ttcgtcgccatccccgccgtg**TTCGC**gttcaactt | 10.17 | - XcampmN\_010100003797 (ACHT01000111:9566-9144) biopolymer transport ExbD1 protein COG0848 Biopolymer transport protein - XcampmN\_010100003792 (ACHT01000111:9140-8727) biopolymer transport ExbD protein COG0848 Biopolymer transport protein - XcampmN\_010100003802 (ACHT01000111:10374-9613) biopolymer transport ExbB protein COG0811 Biopolymer transport proteins |
| ACHT01000742:7085-7137 | **TTCGC**gttgcttggtcagctc**TTCGC**cgacgcgccgcagcaacgcgccacgct | 10.17 |  |
| ACHT01000125:233-263 | gtcgccgcgctggccgaatt**TTCGC**ccgatt | 10.17 | - XcampmN\_010100004279 (ACHT01000125:1064-1) DNA polymerase III subunits gamma and tau COG2812 DNA polymerase III, gamma/tau subunits |
| ACHT01000424:2228-2258 | tctgccagccgctgcagcgcttcggcatatt | 10.15 |  |
| ACHT01000501:6068-6111 | **TTCGC**gcgcgaattcgtcaactcgccgcgccgcgaattcaatat | 10.15 | - XcampmN\_010100016092 (ACHT01000501:5134-3674) amidase COG0154 Asp-tRNAAsn/Glu-tRNAGln amidotransferase A subunit and related amidases - XcampmN\_010100016097 (ACHT01000501:6723-5563) nucleoside hydrolase COG1957 Inosine-uridine nucleoside N-ribohydrolase |
| ACHT01000498:6762-6792 | atcgcctgctcattggcggcttcgtcatgat | 10.14 |  |
| ACHT01000428:5448-5477 | ttcggcctcggccaaggtcatcgccacgat | 10.14 |  |
| ACHT01000543:2116-2156 | ttcggcgtctactcgaacta**TTCGC**tggtggattccaacct | 10.14 | - XcampmN\_010100018096 (ACHT01000543:1-2529) TonB-dependent receptor COG1629 Outer membrane receptor proteins, mostly Fe transport - XcampmN\_010100018101 (ACHT01000543:2720-3931) arabinogalactan endo-1,4-beta-galactosidase COG3867 Arabinogalactan endo-1,4-beta-galactosidase |
| ACHT01000726:10189-10219 | **TTCGC**ccgagcgtctgcagttccgctattgg | 10.12 | - XcampmN\_010100023200 (ACHT01000726:10491-9793) glutathione S-transferase COG0625 Glutathione S-transferase |
| ACHT01000587:3099-3130 | ttcgtcggcgacgaacacgggttccgcaaact | 10.12 |  |
| ACHT01000478:9203-9233 | ttctcagccgtgcctgccag**TTCGC**caatga | 10.11 | - XcampmN\_010100014737 (ACHT01000478:9230-9952) ABC transporter ATP-binding protein COG1131 ABC-type multidrug transport system, ATPase component - XcampmN\_010100014742 (ACHT01000478:9963-10493) putative ABC transporter permease COG1277 ABC-type transport system involved in multi-copper enzyme maturation, permease component |
| ACHT01000563:522-558 | ttccccagccctgcacgacc**TTCGC**ttccaccaaatt | 10.11 | - XcampmN\_010100018788 (ACHT01000563:655-1096) acetyl-CoA acetyltransferase COG0183 Acetyl-CoA acetyltransferase |
| ACHT01000683:10507-10537 | **TTCGC**cccaccaccgcgcgctttgcgatcat | 10.11 | - ribH (ACHT01000683:10559-10095) 6,7-dimethyl-8-ribityllumazine synthase COG0054 Riboflavin synthase beta-chain - nusB (ACHT01000683:10098-9628) transcription antitermination protein NusB COG0781 Transcription termination factor |
| ACHT01000193:8360-8388 | atcgcaccgcgccagttg**TTCGC**cagcct | 10.1 | - XcampmN\_010100005943 (ACHT01000193:8250-6667) flavin monoamine oxidase-related protein COG1231 Monoamine oxidase |
| ACHT01000063:510-541 | ttcgtgcaacgcttccagcacttcgttggagt | 10.1 |  |
| ACHT01000683:6029-6090 | ttcgtgtgggtgaacaccatttccctcgctgcctccagccacgatttggctctttgcaaaat | 10.09 | - XcampmN\_010100021823 (ACHT01000683:5977-4715) hypothetical protein |
| ACHT01000420:20543-20602 | **TTCGC**cgtgggcgatcaa**TTCGC**ggcattccaccgcgaagatgcccatctgccctacctt | 10.07 | - XcampmN\_010100013099 (ACHT01000420:21520-22170) putative SCO1/SenC family protein COG1999 Uncharacterized protein SCO1/SenC/PrrC, involved in biogenesis of respiratory and photosynthetic systems |
| ACHT01000191:7506-7553 | tttgccgcgtgcccagcaa**TTCGC**cgggaccacgcagttccagatcct | 10.07 |  |
| ACHT01000049:10304-10342 | **TTCGC**gctcggcgcgcacggcttcggtgccggccatctt | 10.05 |  |
| ACHT01000442:12783-12816 | ttggcctgcatcggtggcaa**TTCGC**gtgcacggt | 10.03 |  |
| ACHT01000329:2626-2676 | atcgccgctgcatccagtccctcgcgcgggctggtgccgaccacgcacagt | 10.03 | - XcampmN\_010100010539 (ACHT01000329:1752-766) mannan endo-1,4-beta-mannosidase COG2730 Endoglucanase |
| ACHT01000446:1077-1107 | tgcgtccgcgcaccaacctgttcggtgcggt | 10.03 | - asnC (ACHT01000446:1480-86) asparaginyl-tRNA synthetase COG0017 Aspartyl/asparaginyl-tRNA synthetases |
| ACHT01000166:2534-2564 | ttggccttgcgcgccttggc**TTCGC**catagg | 10.02 |  |
| ACHT01000353:1576-1618 | ttccctggcaccgcgacagc**TTCGC**cagccagcgcgacagcgt | 10.02 | - XcampmN\_010100011072 (ACHT01000353:853-1) hypothetical protein COG0526 Thiol-disulfide isomerase and thioredoxins - XcampmN\_010100011077 (ACHT01000353:1341-877) low molecular weight phosphotyrosine protein phosphatase COG0394 Protein-tyrosine-phosphatase - XcampmN\_010100011082 (ACHT01000353:2117-1338) 3-deoxy-manno-octulosonate cytidylyltransferase COG1212 CMP-2-keto-3-deoxyoctulosonic acid synthetase |
| ACHT01000245:9513-9578 | ttcgtccacgatgcccaccag**TTCGC**cgtcgtcgatcaccggcaactgcgagacgtcgtacaactt | 10.01 |  |
| ACHT01000159:1108-1138 | tgcgctatgccacgcacatg**TTCGC**caacga | 10.01 | - XcampmN\_010100004786 (ACHT01000159:1658-732) lipid A biosynthesis lauroyl acyltransferase COG1560 Lauroyl/myristoyl acyltransferase |
