# Supplementary material for: Genome Sequencing of Xanthomonas vasicola Pathovar vasculorum Reveals Variation in Plasmids and Genes Encoding Lipopolysaccharide Synthesis, Type-IV Pilus and Type-III Secretion Effectors
Source: Pathogens. 2014 Mar 18;3(1):211–37. doi: 10.3390/pathogens3010211 (PMC4235730; doi:10.3390/pathogens3010211)
Supplement: Supplementary File 1 — Supplementary Materials (TAR, 5271 KB) [file pathogens-03-00211-s001.tar › supplementary/data/Xvv702_PIP-boxes.html]

Xvv 702 predicted PIP boxes


| Position | Hrp box sequence | Hrp box HMMER score | Downstream genes |
| --- | --- | --- | --- |
| ACHS01000232:7596-7658 | ttcgtcttcccagccaggacttcggtgaagcgaagcgctcccctgcaaacttgcttctacaat | 19.3 | - XcampvN\_010100013710 (ACHS01000232:7971-10058) putative type III effector HolPsyAE |
| ACHS01000032:3830-3860 | **TTCGC**cggccgagccgttacttcagtaacgt | 18.02 | - XcampvN\_010100001649 (ACHS01000032:3946-5181) membrane fusion protein COG0845 Membrane-fusion protein |
| ACHS01000378:10624-10684 | **TTCGC**cgtcccaaccatgacttcgggcataaaacgacattcagaaagctctgcgtcaaact | 17.46 | - XcampvN\_010100020537 (ACHS01000378:9986-8439) outer protein B |
| ACHS01000217:15233-15294 | **TTCGC**cggaccacctattgc**TTCGC**ttcagcagcagctcgccagatgcctgtgaggtacttt | 16.58 | - XcampvN\_010100011959 (ACHS01000217:14912-14106) HrcQ protein COG1886 Flagellar motor switch/type III secretory pathway protein - XcampvN\_010100011969 (ACHS01000217:15791-15153) HpaC protein |
| ACHS01000217:28105-28145 | **TTCGC**ttgcttgctaagtgc**TTCGC**cggacctgagtaaatt | 16.02 | - XcampvN\_010100012044 (ACHS01000217:28336-28809) putative transglycosylase HpaH COG0741 Soluble lytic murein transglycosylase and related regulatory proteins (some contain LysM/invasin domains) |
| ACHS01000203:53868-53907 | **TTCGC**catcgcacagaccta**TTCGC**gcctagtgatcgatt | 15.88 | - XcampvN\_010100010624 (ACHS01000203:53637-54422) hypothetical protein COG3931 Predicted N-formylglutamate amidohydrolase |
| ACHS01000351:3670-3745 | ttcgccttcatgtccatgac**TTCGC**cttctcccgttcatgggaaacg**TTCGC**ctgcaattcaggcatgcctacggt | 15.3 |  |
| ACHS01000217:18827-18889 | **TTCGC**caaaccatgcaggtg**TTCGC**ctgaaagacgggctgactcgcgacggctctaccagaat | 14.99 | - XcampvN\_010100011979 (ACHS01000217:18795-17722) type III secretion system protein HrcU COG1377 Flagellar biosynthesis pathway, component FlhB |
| ACHS01000337:16437-16472 | **TTCGC**acgcagacgggggac**TTCGC**gctgataagct | 14.98 | - XcampvN\_010100019140 (ACHS01000337:16405-14987) aminopeptidase COG2234 Predicted aminopeptidases |
| ACHS01000035:200-230 | **TTCGC**ccgcgcggacaaggttgcggtagagt | 14.94 |  |
| ACHS01000217:28005-28038 | **TTCGC**acgcacaagcgcaat**TTCGC**aaacactct | 14.62 | - XcampvN\_010100012039 (ACHS01000217:27881-27462) hypothetical protein |
| ACHS01000059:4194-4255 | ttcgtcagcgcacacaggagttcaccgctccgccatgtccttgtgctggaggagggtaacgt | 14.54 | - XcampvN\_010100002948 (ACHS01000059:3962-2616) hypothetical protein COG1957 Inosine-uridine nucleoside N-ribohydrolase |
| ACHS01000011:26930-26992 | **TTCGC**catctcgcgcagcgt**TTCGC**atccgaagttttgcacgcagcgcagttccacttacgtt | 14.48 | - XcampvN\_010100000415 (ACHS01000011:26351-20349) transducer protein car |
| ACHS01000326:9527-9558 | **TTCGC**gcagcagatcgatctc**TTCGC**caagct | 14.39 | - XcampvN\_010100018235 (ACHS01000326:8666-8292) putative DNA-binding protein - XcampvN\_010100018240 (ACHS01000326:9373-8753) transcriptional regulator, TetR family protein COG1309 Transcriptional regulator |
| ACHS01000470:2568-2628 | ttcgtcgtcccaaacatgccttcggtgaggcgatgcgaatcggagtgttccacgccaaact | 14.1 | - XcampvN\_010100024415 (ACHS01000470:2187-820) type III effector HopG1 |
| ACHS01000217:3147-3178 | **TTCGC**caggcgatgcaaccgc**TTCGC**ttcagt | 14.05 | - XcampvN\_010100011894 (ACHS01000217:2698-1103) HpaF protein COG4886 Leucine-rich repeat (LRR) protein |
| ACHS01000279:1650-1709 | **TTCGC**tcatgcggacaggtg**TTCGC**cagccgggaatccgcactgccgcgaatagcatatt | 14 | - XcampvN\_010100015143 (ACHS01000279:1593-445) hypothetical protein |
| ACHS01000422:11789-11844 | tccgccagcagatccagttg**TTCGC**tcaagccgcccaattcctgcaaggacagaat | 13.94 |  |
| ACHS01000293:53889-53920 | ttcgtcatctcacacatggt**TTCGC**gctgact | 13.84 | - XcampvN\_010100016583 (ACHS01000293:53696-54007) hypothetical protein |
| ACHS01000184:20534-20564 | **TTCGC**atgcgcctccagcatttcgggcgcct | 13.51 |  |
| ACHS01000217:18903-18963 | **TTCGC**caacgtattccgatg**TTCGC**ttctgaaaagttctggccagcgcagacgcgtagcgt | 13.48 | - XcampvN\_010100011984 (ACHS01000217:19009-19464) HrpB1 - XcampvN\_010100011989 (ACHS01000217:19543-19890) HrpB2 protein - XcampvN\_010100011994 (ACHS01000217:19892-20656) HrcJ protein COG4669 Type III secretory pathway, lipoprotein EscJ |
| ACHS01000069:11830-11866 | tgcgccggcatcaccaccta**TTCGC**cgctggcacact | 13.43 | - XcampvN\_010100003923 (ACHS01000069:12340-11282) alcohol dehydrogenase COG1064 Zn-dependent alcohol dehydrogenases |
| ACHS01000199:4348-4386 | **TTCGC**cgccgtagccgccggtgcgcccggtaaacaacgt | 13.41 |  |
| ACHS01000069:1388-1435 | **TTCGC**ttgcgacgccagcgcttccgggccgcgcgccgccccgcattgt | 13.36 | - XcampvN\_010100003883 (ACHS01000069:1564-1) putative penicillin amidase (fragment) COG2366 Protein related to penicillin acylase |
| ACHS01000341:330-363 | **TTCGC**gatttcggtcgcttt**TTCGC**tcacagact | 13.33 |  |
| ACHS01000228:95-155 | ttcgttcttgcggcctccgg**TTCGC**tcgctttgcacgcatgcgggcagtgctccttagagt | 13.22 | - XcampvN\_010100013075 (ACHS01000228:399-79) IS1404 transposase COG2801 Transposase and inactivated derivatives |
| ACHS01000011:1341-1401 | tacgcccgtggcgctaccga**TTCGC**attgggcaccggccagaccgcagcgctcactatagt | 13.15 | - XcampvN\_010100000285 (ACHS01000011:1528-1713) hypothetical protein - XcampvN\_010100000290 (ACHS01000011:1762-2040) hypothetical protein |
| ACHS01000451:3588-3649 | ttctctttccaagcgaccac**TTCGC**gcagctgcaacgccacgaacgcgcagcgcaatactgt | 13.15 | - XcampvN\_010100023815 (ACHS01000451:3749-7915) hypothetical protein |
| ACHS01000217:12350-12411 | **TTCGC**gcatgaccatacagc**TTCGC**gtactgaccggaatccatgccggcgcacgattagatt | 13.06 | - XcampvN\_010100011934 (ACHS01000217:11455-11213) HrpD6 protein - XcampvN\_010100011939 (ACHS01000217:12404-11466) HrpD5 protein - XcampvN\_010100011944 (ACHS01000217:13213-12401) HpaA protein |
| ACHS01000149:3955-4000 | **TTCGC**cagctgagctatttcgtcgcactggccgacaccggcagctt | 12.95 | - XcampvN\_010100008110 (ACHS01000149:3033-1345) ABC transporter permease and ATP-binding protein COG2274 ABC-type bacteriocin/lantibiotic exporters, contain an N-terminal double-glycine peptidase domain - XcampvN\_010100008115 (ACHS01000149:4022-3123) LysR family transcriptional regulator COG0583 Transcriptional regulator |
| ACHS01000459:4249-4280 | **TTCGC**cggcgaagccgaccacttcggcatcga | 12.82 |  |
| ACHS01000231:9623-9665 | **TTCGC**catcgggaaacacgtcttcggccaccgctgcgtaagtt | 12.69 |  |
| ACHS01000230:2263-2292 | ttcgttttctgcccaaatt**TTCGC**cagtct | 12.67 | - XcampvN\_010100013155 (ACHS01000230:2223-1177) rod shape-determining protein MreB COG1077 Actin-like ATPase involved in cell morphogenesis |
| ACHS01000149:7151-7182 | ttcgtcgccctgcccggcggcttcggcaccat | 12.6 | - XcampvN\_010100008125 (ACHS01000149:6668-6030) thymidylate synthase COG1739 Uncharacterized conserved protein - XcampvN\_010100008135 (ACHS01000149:7482-6889) hypothetical protein COG1611 Predicted Rossmann fold nucleotide-binding protein |
| ACHS01000157:839-872 | tgcgcctgcgcgacaagcgc**TTCGC**cgccacgtt | 12.59 | - XcampvN\_010100008547 (ACHS01000157:101-1) hypothetical protein - sbcB (ACHS01000157:1479-34) exonuclease I COG2925 Exonuclease I |
| ACHS01000054:5159-5197 | **TTCGC**tatcttcggcgcctattcgggtgggggatacggt | 12.57 | - XcampvN\_010100002441 (ACHS01000054:4675-5859) phage-related integrase COG0582 Integrase |
| ACHS01000478:32108-32146 | tgcgccagagcaggcagtacctcgcgaaaccgctaaaat | 12.55 | - XcampvN\_010100025160 (ACHS01000478:32208-31120) 5'-nucleotidase, lipoprotein e(P4) family COG2503 Predicted secreted acid phosphatase |
| ACHS01000191:24383-24413 | ttccccttaggagggacaacgtcgctacact | 12.55 | - XcampvN\_010100010262 (ACHS01000191:24307-24456) hypothetical protein - XcampvN\_010100010267 (ACHS01000191:24617-25611) putative integrase/recombinase COG0582 Integrase |
| ACHS01000219:22038-22099 | **TTCGC**caggggacgcgtaactgcgccaacgaacaaaagtcccggcttgcaagtcgttatcct | 12.49 | - XcampvN\_010100012281 (ACHS01000219:21901-21575) hypothetical protein |
| ACHS01000114:15876-15907 | ttcggcacgccacgcggatc**TTCGC**ccagata | 12.47 | - XcampvN\_010100005257 (ACHS01000114:16302-16427) hypothetical protein |
| ACHS01000293:44476-44507 | ttcgtgctgcgcatgcgccat**TTCGC**caatca | 12.46 |  |
| ACHS01000068:10570-10613 | **TTCGC**aaacgcagccattcg**TTCGC**attaccgccgtcgcagcga | 12.43 | - XcampvN\_010100003818 (ACHS01000068:10616-9468) xylose repressor-like protein COG1940 Transcriptional regulator/sugar kinase |
| ACHS01000450:22230-22283 | ttcggcgtcgtaacgcaccgc**TTCGC**cgagccgcgcatcggcgcggtacagagt | 12.41 | - XcampvN\_010100023695 (ACHS01000450:23000-23257) hypothetical protein |
| ACHS01000400:2322-2354 | **TTCGC**attgccagcgagggcttccccgtatacg | 12.38 | - XcampvN\_010100021467 (ACHS01000400:2832-1093) gamma-glutamyltranspeptidase COG0405 Gamma-glutamyltransferase |
| ACHS01000145:7591-7650 | **TTCGC**cgaccagcgccagcac**TTCGC**cggcgcgcaattcgaaatccagatgccgcaccgt | 12.37 |  |
| ACHS01000067:1276-1317 | **TTCGC**tcgcgatccgattactgcgctggcatatcgataaatt | 12.35 | - XcampvN\_010100003643 (ACHS01000067:1110-1877) putative secreted protein |
| ACHS01000276:5908-5968 | **TTCGC**cattgatgacagaatttcacataccggcaatgtgcttgtttgctcgcaattaccat | 12.35 |  |
| ACHS01000247:2447-2477 | ttcgtgttcggcaatggtttttcggcaggat | 12.3 | - XcampvN\_010100014107 (ACHS01000247:2506-3288) hypothetical protein |
| ACHS01000303:11373-11437 | ttctgcagcgcagcggccac**TTCGC**gctgcgaggcaaaaccggacggctggaacttgtccagatt | 12.28 |  |
| ACHS01000219:44024-44055 | tgcgccagcgcggcggccac**TTCGC**tcaaggt | 12.21 |  |
| ACHS01000337:12034-12086 | atcgccttggaaatcaaaat**TTCGC**ctgagtcgccctaaccctcagccagact | 12.21 |  |
| ACHS01000412:2159-2202 | **TTCGC**ccagccgccgcgcttt**TTCGC**ggccggcagcggcaccgt | 12.16 |  |
| ACHS01000119:43367-43402 | **TTCGC**cgccatagacggcatt**TTCGC**tgcgcatttt | 12.16 |  |
| ACHS01000203:81060-81132 | tacgcagccgcgcacagtgc**TTCGC**gtagcgccatcgccgcctcttgcagcgcccgtttcggtcggctacatt | 12.15 | - XcampvN\_010100010794 (ACHS01000203:81163-81636) MarR family transcriptional regulator COG1846 Transcriptional regulators - XcampvN\_010100010799 (ACHS01000203:81682-82509) p-hydroxycinnamoyl CoA hydratase/lyase COG1024 Enoyl-CoA hydratase/carnithine racemase |
| ACHS01000315:13300-13347 | **TTCGC**tacgataaagatgac**TTCGC**cccaaaggcttcgtgcttagcgt | 12.12 |  |
| ACHS01000191:3512-3542 | ttcgtcaatatcggcgcggtatcgctagact | 12.06 |  |
| ACHS01000060:20362-20392 | **TTCGC**tcgcagttgggtttg**TTCGC**caacct | 11.98 | - XcampvN\_010100003043 (ACHS01000060:20079-21152) 3-isopropylmalate dehydrogenase COG0473 Isocitrate/isopropylmalate dehydrogenase |
| ACHS01000119:25389-25439 | atcgccatcaccaacgcatc**TTCGC**gcccggtgctggaggggtagtagcgt | 11.98 |  |
| ACHS01000217:31188-31230 | ttcggatgcggaatgatccc**TTCGC**aatatatgtgtccagaat | 11.97 | - XcampvN\_010100012049 (ACHS01000217:30312-29272) hypothetical protein |
| ACHS01000030:30253-30282 | ttcgtcggccagccgcgcttgcgccagctt | 11.95 |  |
| ACHS01000151:2732-2775 | **TTCGC**ctggaaagaagcaacttcacggtgctcaacaagcaactt | 11.95 | - XcampvN\_010100008185 (ACHS01000151:2830-2537) hypothetical protein |
| ACHS01000009:1703-1744 | **TTCGC**tgtgccgttgatgat**TTCGC**tggtgggtgggtaccct | 11.94 |  |
| ACHS01000238:3708-3747 | **TTCGC**cagcgaagccacgcagttcggacaggcggctcaat | 11.94 |  |
| ACHS01000182:2964-3033 | atcgcctgggtcgctgccgc**TTCGC**ctccacgccgtttctcaagagcgccggcaagccgtggcatagcct | 11.94 | - XcampvN\_010100009622 (ACHS01000182:3259-1490) putative siderophore biosynthesis protein COG4264 Siderophore synthetase component |
| ACHS01000117:27200-27277 | ttcggcaccaccgatgccac**TTCGC**atggtttccgcaaggccggtctggtcaacgggcagatcggcggtttctacaat | 11.93 | - XcampvN\_010100005582 (ACHS01000117:28090-26771) sugar transporter COG0477 Permeases of the major facilitator superfamily |
| ACHS01000442:46-76 | **TTCGC**tgcagcattcaggct**TTCGC**cagcgc | 11.91 | - glk (ACHS01000442:915-1) glucokinase COG0837 Glucokinase |
| ACHS01000109:6431-6545 | **TTCGC**ttttcgggcaatgacttcgtgttttcaccgcaatgccgggtgtcaaagcaaataccagctgactggattgtggcattgcaacaaaaatctgaagtccccgcgtctagagt | 11.9 | - XcampvN\_010100004977 (ACHS01000109:6399-5584) beta-ketoadipate enol-lactone hydrolase COG0596 Predicted hydrolases or acyltransferases (alpha/beta hydrolase superfamily) |
| ACHS01000089:1785-1824 | ttcgttctggtaactgcttcttcgtgcgatgtgcgtaaat | 11.88 |  |
| ACHS01000079:675-705 | **TTCGC**cgcgcagcatgttattcacataagct | 11.88 |  |
| ACHS01000108:4590-4621 | ttcgtcggcgccgcgcggcag**TTCGC**tgaact | 11.85 | - XcampvN\_010100004917 (ACHS01000108:3946-3773) hypothetical protein |
| ACHS01000383:3423-3452 | ttcgttggcgatgcgattgttcggcaacat | 11.84 | - XcampvN\_010100020922 (ACHS01000383:2743-2273) F0F1 ATP synthase subunit B COG0711 F0F1-type ATP synthase, subunit b - XcampvN\_010100020927 (ACHS01000383:3163-2858) F0F1 ATP synthase subunit C COG0636 F0F1-type ATP synthase, subunit c/Archaeal/vacuolar-type H -ATPase, subunit K - XcampvN\_010100020932 (ACHS01000383:4036-3236) F0F1 ATP synthase subunit A COG0356 F0F1-type ATP synthase, subunit a |
| ACHS01000273:1372-1400 | **TTCGC**cgcgcatgaagcgctgcgccaaat | 11.79 | - XcampvN\_010100014938 (ACHS01000273:1789-2073) resolvase domain-containing protein COG1961 Site-specific recombinases, DNA invertase Pin homologs |
| ACHS01000281:14489-14610 | **TTCGC**atgcggccgcatgca**TTCGC**gctgggatctgcatgcgcgctagtgcctgcgcgatgctgccaggcccgcgcggcacgacgcgctcactggatcgcgcatgcgtctcaaggccacagt | 11.79 | - XcampvN\_010100015513 (ACHS01000281:14630-15547) 2-keto-3-deoxygluconate permease |
| ACHS01000255:16266-16296 | tacgccggcgcacacggtct**TTCGC**tgcctt | 11.73 | - XcampvN\_010100014653 (ACHS01000255:17216-17316) hypothetical protein - XcampvN\_010100014648 (ACHS01000255:16665-17144) hypothetical protein |
| ACHS01000207:2938-2969 | **TTCGC**ggtggcgtagctgtggcttcggtacat | 11.71 | - XcampvN\_010100011129 (ACHS01000207:2907-136) TonB-dependent receptor COG1629 Outer membrane receptor proteins, mostly Fe transport |
| ACHS01000217:16615-16671 | **TTCGC**aaaagcgcaccggat**TTCGC**atcacccttgtcgatgcggctgtcgccacagt | 11.68 | - XcampvN\_010100011969 (ACHS01000217:15791-15153) HpaC protein - XcampvN\_010100011974 (ACHS01000217:17677-15791) HrcV protein COG4789 Type III secretory pathway, component EscV |
| ACHS01000030:11641-11688 | tgcgcttgcgcggacatcgc**TTCGC**agggtcgcgaccgcgcgcaacct | 11.67 | - XcampvN\_010100001489 (ACHS01000030:11715-11137) NDP-hexose isomerase - XcampvN\_010100001484 (ACHS01000030:11140-10469) hypothetical protein COG0110 Acetyltransferase (isoleucine patch superfamily) |
| ACHS01000296:44574-44608 | **TTCGC**tcccaacgtcatcct**TTCGC**agcgttcagt | 11.64 | - XcampvN\_010100017023 (ACHS01000296:44712-44371) hypothetical protein - XcampvN\_010100017018 (ACHS01000296:44374-42701) feruloyl esterase |
| ACHS01000225:38821-38862 | **TTCGC**ccaggctcaaagcta**TTCGC**atcagcagcgctatacg | 11.64 | - XcampvN\_010100012975 (ACHS01000225:38916-38542) hypothetical protein - XcampvN\_010100012970 (ACHS01000225:38371-37028) hypothetical protein |
| ACHS01000428:11257-11291 | **TTCGC**gggttcggcagttt**TTCGC**tgcattaccgt | 11.63 | - ihfB (ACHS01000428:11124-11435) integration host factor subunit beta COG0776 Bacterial nucleoid DNA-binding protein - XcampvN\_010100022626 (ACHS01000428:11503-11808) hypothetical protein - XcampvN\_010100022631 (ACHS01000428:11815-12993) tetratricopeptide repeat protein COG2956 Predicted N-acetylglucosaminyl transferase |
| ACHS01000471:30407-30437 | **TTCGC**cagccgcaccagcttctcgtccgagt | 11.61 | - XcampvN\_010100024600 (ACHS01000471:29614-29507) hypothetical protein |
| ACHS01000117:25388-25438 | **TTCGC**actagaggcgggttg**TTCGC**gcggtggcggccgcttgtgcgcagat | 11.61 |  |
| ACHS01000217:7033-7095 | ttcgtgtgcggcgcggccgg**TTCGC**agtgc**TTCGC**taaagcgcgtatctcggcgcaagaaaat | 11.59 | - XcampvN\_010100011914 (ACHS01000217:7155-7601) hypothetical protein - XcampvN\_010100011919 (ACHS01000217:7856-9616) outer protein F1 |
| ACHS01000231:23042-23073 | **TTCGC**cggcgctgccgccgatgcgtttacctt | 11.59 | - hslU (ACHS01000231:22571-21228) ATP-dependent protease ATP-binding subunit HslU COG1220 ATP-dependent protease HslVU (ClpYQ), ATPase subunit - XcampvN\_010100013305 (ACHS01000231:23247-22696) ATP-dependent protease peptidase subunit COG5405 ATP-dependent protease HslVU (ClpYQ), peptidase subunit |
| ACHS01000231:72345-72418 | tgcgcagccgcatccgtgac**TTCGC**ggccgtgacagcgcaacccctggtgtgcgttgccgcgatctcgtatttt | 11.56 |  |
| ACHS01000477:22073-22124 | ttcttcagcagcaccagcga**TTCGC**gcgcagtgcgtcgcgccagcgcatcat | 11.5 |  |
| ACHS01000288:451-482 | **TTCGC**tggatgcgccaggt**TTCGC**cgcatgct | 11.5 |  |
| ACHS01000423:10810-10856 | ttcgtgcatcagcgccaacac**TTCGC**tgcgcagatcctgggcaatct | 11.47 |  |
| ACHS01000069:73604-73637 | ttctttcgagagcacgtggc**TTCGC**acgtaccct | 11.45 |  |
| ACHS01000117:27505-27535 | ttcgtgatcggcgcgggtttttctgcagcgt | 11.44 | - XcampvN\_010100005582 (ACHS01000117:28090-26771) sugar transporter COG0477 Permeases of the major facilitator superfamily |
| ACHS01000208:27473-27522 | atcgtcagcgacgccggcactccgctggtcagcgacccgggcttcaaact | 11.44 | - XcampvN\_010100011389 (ACHS01000208:26954-26229) hypothetical protein - XcampvN\_010100011394 (ACHS01000208:27765-26938) hypothetical protein COG0313 Predicted methyltransferases |
| ACHS01000477:26100-26130 | tgcaccagcctaccgaggacttcgtcaaact | 11.44 | - XcampvN\_010100024925 (ACHS01000477:25523-26656) prophage Lp2 protein 6 COG4748 Uncharacterized conserved protein |
| ACHS01000381:24568-24597 | **TTCGC**tgcgcacgcaagacatcgccaagtt | 11.38 |  |
| ACHS01000159:5056-5107 | **TTCGC**cggcgcgcgaggc**TTCGC**cggtatcgcggttatgcatctcccaacgt | 11.37 |  |
| ACHS01000378:2757-2788 | **TTCGC**caaccctagcgcgcacttcggcaatgt | 11.37 | - XcampvN\_010100020492 (ACHS01000378:2204-1965) hypothetical protein - XcampvN\_010100020497 (ACHS01000378:3052-2303) hypothetical protein COG2353 Uncharacterized conserved protein |
| ACHS01000451:15322-15359 | atcgccggcgcgctgtgcc**TTCGC**gggcaggacacact | 11.36 | - XcampvN\_010100023850 (ACHS01000451:15689-15883) hypothetical protein - XcampvN\_010100023845 (ACHS01000451:14482-15645) hypothetical protein COG3146 Uncharacterized protein conserved in bacteria - aat (ACHS01000451:15914-16642) leucyl/phenylalanyl-tRNA--protein transferase COG2360 Leu/Phe-tRNA-protein transferase |
| ACHS01000208:14012-14043 | ttcggcttcggcacgcagctt**TTCGC**cgcact | 11.34 |  |
| ACHS01000133:360-402 | atcgcatccgcacccagatgttcggcctgatcgccatgaccct | 11.32 | - XcampvN\_010100006818 (ACHS01000133:353-1939) periplasmic serine protease MucD COG0265 Trypsin-like serine proteases, typically periplasmic, contain C-terminal PDZ domain |
| ACHS01000290:7533-7572 | ttggccagcgcacgcaggcg**TTCGC**ggtagtcgcgcagat | 11.32 |  |
| ACHS01000114:25711-25769 | atcgccagcgaataggcctc**TTCGC**ccgtggagcagcccagcacccagatgcgcaggct | 11.29 |  |
| ACHS01000231:43340-43371 | **TTCGC**cagcgccgtggccggcttcgtcaccgt | 11.26 | - XcampvN\_010100013415 (ACHS01000231:43698-41761) glucosyltransferase MdoH COG2943 Membrane glycosyltransferase |
| ACHS01000100:2985-3055 | **TTCGC**cgggtaaggcacgtttgcgcggcgggcagcggttgcgcgcgaccgatatagcggtaccggcagatt | 11.23 | - XcampvN\_010100004785 (ACHS01000100:2239-3663) 3-phosphoshikimate 1-carboxyvinyltransferase COG0128 5-enolpyruvylshikimate-3-phosphate synthase |
| ACHS01000365:2078-2166 | ttcgtatcagtcaccacgca**TTCGC**gcggcatgccaacagcgcggcattcggtggtttactaaagcagcgatgtcacgcacatcaaaat | 11.23 | - XcampvN\_010100020297 (ACHS01000365:2704-3658) hypothetical protein |
| ACHS01000219:45150-45212 | **TTCGC**cgttgttgaacggcg**TTCGC**ctgcgcctgctgccctgggccagcagttgcgctacagt | 11.22 | - XcampvN\_010100012426 (ACHS01000219:45328-46803) anthranilate synthase component I COG0147 Anthranilate/para-aminobenzoate synthases component I |
| ACHS01000290:237-307 | ttggccagcacccgcggcgc**TTCGC**cgcgctgtgccagcgagaagatcatccgcgaggcgccatacagatt | 11.2 |  |
| ACHS01000148:6431-6461 | tgcatcattcaaggcacagc**TTCGC**tagcgt | 11.18 |  |
| ACHS01000061:12594-12663 | ttcgtccgcactgcgactcc**TTCGC**agcgccagccattaggacgaatgccatgaacgccgcgcccaccgt | 11.17 | - XcampvN\_010100003103 (ACHS01000061:12644-14521) thiamine biosynthesis protein ThiC COG0422 Thiamine biosynthesis protein ThiC |
| ACHS01000304:2511-2542 | **TTCGC**accggcgcgcagcaattcggactgatt | 11.16 |  |
| ACHS01000247:3869-3930 | **TTCGC**catcggtcacgcttattcgggcaatgccgcgcgtgcgtgattgggcaacggaacaat | 11.16 | - XcampvN\_010100014112 (ACHS01000247:3957-4736) hypothetical protein |
| ACHS01000068:9347-9386 | tgcgcaccgcaggcagcgt**TTCGC**atacaggtggcatatt | 11.15 | - XcampvN\_010100003813 (ACHS01000068:9449-7131) avirulence protein COG0584 Glycerophosphoryl diester phosphodiesterase |
| ACHS01000311:5079-5113 | **TTCGC**agttcaacacagtttttccgggcttcgaat | 11.15 | - XcampvN\_010100017775 (ACHS01000311:4687-4499) hypothetical protein - XcampvN\_010100017770 (ACHS01000311:4496-2916) P-type conjugative transfer protein TrbL COG3846 Type IV secretory pathway, TrbL components - XcampvN\_010100017780 (ACHS01000311:5316-4690) P-type conjugative transfer protein TrbJ COG5314 Conjugal transfer/entry exclusion protein |
| ACHS01000281:32037-32088 | **TTCGC**ctgggtgggccggaattcggcttgagccaaagccagatcggcatgat | 11.12 | - XcampvN\_010100015613 (ACHS01000281:31577-31473) hypothetical protein - XcampvN\_010100015618 (ACHS01000281:32857-31577) MFS transporter COG0477 Permeases of the major facilitator superfamily |
| ACHS01000056:16734-16765 | ttggccagcagctcaagctcttcggccaccat | 11.12 |  |
| ACHS01000485:20772-20806 | **TTCGC**ccggcagatccaaccc**TTCGC**cgaaagaat | 11.07 |  |
| ACHS01000351:4114-4144 | atcgtctgtgcgcaatcgacttcgtcacagt | 11.03 | - XcampvN\_010100019775 (ACHS01000351:4695-5594) N-acetylmuramoyl-L-alanine amidase COG0860 N-acetylmuramoyl-L-alanine amidase |
| ACHS01000322:731-773 | atcgccttggcatcgatcat**TTCGC**cgacgctgtcgcgaccat | 11.03 |  |
| ACHS01000247:2246-2275 | **TTCGC**ccacgcttgcacagcttcgttcatt | 11.03 | - XcampvN\_010100014092 (ACHS01000247:2109-535) putative serine/threonine protein kinase COG0515 Serine/threonine protein kinase |
| ACHS01000282:10956-11002 | accgccagcgcacccagcgt**TTCGC**acagcaggtcctggatcaggtt | 11.01 |  |
| ACHS01000279:8248-8278 | **TTCGC**gaacacgggcacgacgtcgccagtgt | 11.01 | - XcampvN\_010100015178 (ACHS01000279:7434-8318) biotin synthesis protein COG0500 SAM-dependent methyltransferases - XcampvN\_010100015188 (ACHS01000279:8567-9472) peptide-aspartate beta-dioxygenase COG3555 Aspartyl/asparaginyl beta-hydroxylase and related dioxygenases |
| ACHS01000219:6542-6573 | **TTCGC**cgtcgttggccaccacttccttgtact | 11 |  |
| ACHS01000064:15485-15515 | ttcgtttcaggccccgtcagtacgccagcgt | 11 | - XcampvN\_010100003403 (ACHS01000064:15561-17732) guanosine-3',5'-bis(diphosphate) 3'-pyrophosphohydrolase COG0317 Guanosine polyphosphate pyrophosphohydrolases/synthetases |
| ACHS01000325:33621-33651 | ttcggcctgcgcgacgtcgg**TTCGC**tacccg | 10.99 | - XcampvN\_010100018175 (ACHS01000325:34235-35026) hypothetical protein |
| ACHS01000458:5591-5620 | ttcgtcgtccaggcgcgtcgtcgctaccga | 10.98 | - XcampvN\_010100024120 (ACHS01000458:4921-4466) hypothetical protein |
| ACHS01000225:36699-36743 | atcgtcatcgacggcggcaattcgtactacaagga**TTCGC**aacgt | 10.98 | - XcampvN\_010100012965 (ACHS01000225:37007-36066) 6-phosphogluconate dehydrogenase-like protein COG1023 Predicted 6-phosphogluconate dehydrogenase |
| ACHS01000145:4035-4093 | atcgcctgcagatcggctgc**TTCGC**gcttggcgttggccactgcggccggtgccagttt | 10.97 |  |
| ACHS01000230:4894-4931 | **TTCGC**tgtcgatcagcgcttcggggatcgccgcacaat | 10.91 |  |
| ACHS01000484:40028-40062 | atcgcatgccgctgcaggct**TTCGC**cgtacaccat | 10.9 |  |
| ACHS01000399:6522-6574 | **TTCGC**cggcgaaacccagatcttcatcctgcctggctaccgcatccgcagcgt | 10.9 | - tgt (ACHS01000399:6257-5112) queuine tRNA-ribosyltransferase COG0343 Queuine/archaeosine tRNA-ribosyltransferase - queA (ACHS01000399:7402-6332) S-adenosylmethionine:tRNA ribosyltransferase-isomerase COG0809 S-adenosylmethionine:tRNA-ribosyltransferase-isomerase (queuine synthetase) |
| ACHS01000219:34923-34957 | **TTCGC**tcactgcgcgcgaga**TTCGC**aatgtagctt | 10.9 | - XcampvN\_010100012366 (ACHS01000219:35034-35519) hypothetical protein - XcampvN\_010100012371 (ACHS01000219:35516-37489) outer protein F2 |
| ACHS01000170:42863-42896 | **TTCGC**catcttctcgaccgcttcggcgctaccgg | 10.87 | - XcampvN\_010100009497 (ACHS01000170:42205-42023) hypothetical protein - XcampvN\_010100009502 (ACHS01000170:42912-42496) hypothetical protein |
| ACHS01000203:51247-51280 | **TTCGC**atggtaggcggttag**TTCGC**ttgttaagt | 10.87 | - XcampvN\_010100010609 (ACHS01000203:51538-52113) hypothetical protein COG0778 Nitroreductase - XcampvN\_010100010614 (ACHS01000203:52110-53096) exodeoxyribonuclease IX COG0258 5'-3' exonuclease (including N-terminal domain of PolI) |
| ACHS01000184:18222-18280 | ttcggtcgaggcatcacttt**TTCGC**tgctcgccagggcatggattcgaaatcgtatttt | 10.86 |  |
| ACHS01000325:12595-12638 | atcgcgctcagcggcagcac**TTCGC**cccacggcgcgtccacggt | 10.84 |  |
| ACHS01000292:685-736 | **TTCGC**gcttgaccgggaatttcggcacggcggtgtaggcgggcgcgcacaat | 10.84 |  |
| ACHS01000383:2670-2704 | **TTCGC**cggcctgatctggatcgtcgcgaccaaaat | 10.84 | - XcampvN\_010100020922 (ACHS01000383:2743-2273) F0F1 ATP synthase subunit B COG0711 F0F1-type ATP synthase, subunit b - XcampvN\_010100020917 (ACHS01000383:2269-1742) F0F1 ATP synthase subunit delta COG0712 F0F1-type ATP synthase, delta subunit (mitochondrial oligomycin sensitivity protein) |
| ACHS01000064:20443-20499 | ttcggattcgttgcggcatc**TTCGC**aaggatcggccggtcacgcgaacgcctaggat | 10.83 |  |
| ACHS01000331:2779-2813 | **TTCGC**tgctgcgagaagggt**TTCGC**gtatgacgct | 10.81 | - XcampvN\_010100018765 (ACHS01000331:2486-1209) transglycosylase COG2951 Membrane-bound lytic murein transglycosylase B |
| ACHS01000221:16122-16163 | **TTCGC**cggcattggccacgat**TTCGC**gcagcggggcttccat | 10.8 |  |
| ACHS01000403:6954-7010 | ttggcctgcagcgcggtgtcttcggtctgcgcggccagggcctgcgcccagtacatt | 10.79 |  |
| ACHS01000448:9889-9920 | **TTCGC**cattgccacaatgagtccgctatgaat | 10.79 | - XcampvN\_010100023510 (ACHS01000448:9825-9478) hypothetical protein COG4244 Predicted membrane protein - XcampvN\_010100023515 (ACHS01000448:9994-9902) hypothetical protein - XcampvN\_010100023505 (ACHS01000448:9481-8150) L-sorbosone dehydrogenase COG2133 Glucose/sorbosone dehydrogenases |
| ACHS01000126:26192-26227 | ttcgcc**TTCGC**ggctaatgcc**TTCGC**cgtacaggat | 10.79 |  |
| ACHS01000330:4734-4792 | **TTCGC**cagcggcgtcacaactgcgttgatcggccccagcggcgccggcaaatccaccgt | 10.78 | - XcampvN\_010100018725 (ACHS01000330:4861-4124) ABC transporter ATP-binding protein COG1125 ABC-type proline/glycine betaine transport systems, ATPase components - XcampvN\_010100018720 (ACHS01000330:4127-2655) ABC transporter permease and substrate-binding protein COG1732 Periplasmic glycine betaine/choline-binding (lipo)protein of an ABC-type transport system (osmoprotectant binding protein) |
| ACHS01000339:3886-3929 | ttcgtcggcggcggcgtgacc**TTCGC**ggccaagccgcgcagctt | 10.78 | - rplW (ACHS01000339:4260-4559) 50S ribosomal protein L23 COG0089 Ribosomal protein L23 - rplD (ACHS01000339:3658-4263) 50S ribosomal protein L4 COG0088 Ribosomal protein L4 - rplB (ACHS01000339:4570-5397) 50S ribosomal protein L2 COG0090 Ribosomal protein L2 |
| ACHS01000279:4780-4846 | atcgccatcacggtcacttc**TTCGC**cgaacagcaacggctggatcggtgtgtcagaagccatcaatt | 10.77 |  |
| ACHS01000426:3294-3325 | ttcggcacgcggatcgtcttc**TTCGC**catcct | 10.76 | - XcampvN\_010100022506 (ACHS01000426:4063-4362) YciI-like protein COG2350 Uncharacterized protein conserved in bacteria |
| ACHS01000471:14871-14952 | **TTCGC**tcggtgcgccacctctgcgcaccggtagcaaaccgccatctcgtcgaagaaattaagtcatcgaaagtaagtaaatt | 10.75 | - XcampvN\_010100024515 (ACHS01000471:14592-13615) hypothetical protein COG0584 Glycerophosphoryl diester phosphodiesterase |
| ACHS01000190:19762-19819 | ttcttctgcggatccttggt**TTCGC**cggcggaggtcgagaccgcgtcgaagccaatgt | 10.74 | - XcampvN\_010100010117 (ACHS01000190:18849-18262) hypothetical protein COG0494 NTP pyrophosphohydrolases including oxidative damage repair enzymes |
| ACHS01000207:14964-15016 | **TTCGC**gcaggtactccagata**TTCGC**gcacatgccgctcgcgtgcggcaacgt | 10.74 | - XcampvN\_010100011189 (ACHS01000207:15722-15838) hypothetical protein |
| ACHS01000221:7297-7326 | ctcgcccgacaagccggccttcgtcaccgt | 10.72 | - XcampvN\_010100012730 (ACHS01000221:7625-7140) acetyl-CoA carboxylase biotin carboxyl carrier protein subunit COG0511 Biotin carboxyl carrier protein - XcampvN\_010100012715 (ACHS01000221:6684-5317) acetyl-CoA carboxylase biotin carboxylase subunit COG0439 Biotin carboxylase - XcampvN\_010100012725 (ACHS01000221:7147-6674) hypothetical protein |
| ACHS01000251:1970-2000 | ctcgccggccgatccggatctgcgcctgaat | 10.71 | - XcampvN\_010100014303 (ACHS01000251:1455-2783) two-component system sensor protein COG0642 Signal transduction histidine kinase - XcampvN\_010100014313 (ACHS01000251:2900-5002) polyphosphate kinase COG0855 Polyphosphate kinase |
| ACHS01000203:33996-34026 | ttcgtccccggcgccgcgcgctcggtacaga | 10.7 | - rbn (ACHS01000203:34272-32995) ribonuclease BN/unknown domain fusion protein COG1295 Predicted membrane protein |
| ACHS01000484:52268-52298 | atcgcgacccgaagaacttcttccccaactt | 10.69 | - XcampvN\_010100025770 (ACHS01000484:51991-53151) ABC transporter permease COG0577 ABC-type antimicrobial peptide transport system, permease component - XcampvN\_010100025775 (ACHS01000484:53132-54484) ABC transporter permease COG0577 ABC-type antimicrobial peptide transport system, permease component |
| ACHS01000069:50439-50485 | ttctccagccagcgcgcagcttcggcgaagcactcctggcgtataca | 10.69 | - XcampvN\_010100004118 (ACHS01000069:49615-49418) hypothetical protein |
| ACHS01000066:4353-4412 | **TTCGC**tttgcgcgcagcaacttcgttttgcgattgagaccagaggcaacagcaataaagg | 10.66 |  |
| ACHS01000477:10727-10768 | ctcgcagcgcagcaagcctttcggccgccggctcggtacagt | 10.65 | - XcampvN\_010100024885 (ACHS01000477:11017-12354) xylose isomerase COG2115 Xylose isomerase |
| ACHS01000219:36956-36986 | ttacccggcgagcccaacaa**TTCGC**cagtat | 10.62 | - XcampvN\_010100012371 (ACHS01000219:35516-37489) outer protein F2 |
| ACHS01000428:8217-8252 | tccgccgccgcggcaaggtcttcgtgatctgcaagt | 10.6 | - XcampvN\_010100022596 (ACHS01000428:7427-7287) hypothetical protein - XcampvN\_010100022601 (ACHS01000428:7943-7602) hypothetical protein - rpmJ (ACHS01000428:8310-8185) 50S ribosomal protein L36 COG0257 Ribosomal protein L36 - XcampvN\_010100022591 (ACHS01000428:7366-6332) hypothetical protein COG2957 Peptidylarginine deiminase and related enzymes |
| ACHS01000218:15807-15878 | ttcgagacgcgatcaagatc**TTCGC**agaccatgcgaggcaacgcgcatcctcgtccgcgctttgcacacact | 10.57 |  |
| ACHS01000117:14945-14982 | **TTCGC**cgcgctgccgaacgta**TTCGC**atcacgcagcct | 10.54 | - XcampvN\_010100005517 (ACHS01000117:15046-14144) LysR family transcriptional regulator COG0583 Transcriptional regulator |
| ACHS01000472:4802-4841 | tccgccccttcagcaattct**TTCGC**tgcgttctgtatcct | 10.54 |  |
| ACHS01000066:3794-3828 | atcgcc**TTCGC**atcgaacggttcgggttgcaccgt | 10.53 |  |
| ACHS01000306:980-1024 | ttcgtccagcacgcagat**TTCGC**aggcgtgcagcgacaccacctt | 10.52 | - XcampvN\_010100017680 (ACHS01000306:1679-2020) thioredoxin COG0526 Thiol-disulfide isomerase and thioredoxins |
| ACHS01000333:32653-32681 | ttctccggtgtgctcggta**TTCGC**cagat | 10.51 | - XcampvN\_010100019045 (ACHS01000333:32167-33237) RND efflux membrane fusion protein COG0845 Membrane-fusion protein - XcampvN\_010100019050 (ACHS01000333:33234-36359) RND superfamily protein COG0841 Cation/multidrug efflux pump |
| ACHS01000353:2448-2481 | ttcgtctgtctggcgtgtcg**TTCGC**ctacaacct | 10.49 | - XcampvN\_010100019902 (ACHS01000353:3574-2291) metabolite:H symporter family protein COG0477 Permeases of the major facilitator superfamily |
| ACHS01000131:22492-22540 | **TTCGC**cggacagccccac**TTCGC**cgaacgcgatggtcttttcggacaat | 10.49 |  |
| ACHS01000351:22977-23006 | **TTCGC**tgccagacccggtgttcgacacggt | 10.48 | - XcampvN\_010100019860 (ACHS01000351:23101-21257) hypothetical protein COG2203 FOG: GAF domain |
| ACHS01000409:7785-7823 | **TTCGC**cgcccagcaagaccttggcgcccttttccacgct | 10.46 |  |
| ACHS01000221:13755-13786 | **TTCGC**cagccgtggccaccacttccacacctt | 10.46 | - groES (ACHS01000221:14351-14638) co-chaperonin GroES COG0234 Co-chaperonin GroES (HSP10) |
| ACHS01000138:3302-3332 | **TTCGC**catcgaccttggc**TTCGC**cgtagaag | 10.46 |  |
| ACHS01000133:28274-28344 | ttcgtcggcaaacgcgccactgcgctgcgcggcctgcgcgcgttccaccgaggcaatggcgaatgcatcct | 10.44 | - XcampvN\_010100006978 (ACHS01000133:28906-29078) hypothetical protein |
| ACHS01000131:42552-42594 | ttggccaggtgctccagtgc**TTCGC**ggccgttgcgtgcaatct | 10.41 |  |
| ACHS01000064:3910-3966 | **TTCGC**gcgggccatcccaggc**TTCGC**gctcggcgtcgcgctcgcgacagaacaaaat | 10.4 |  |
| ACHS01000451:25664-25713 | **TTCGC**ccagtcgcacgat**TTCGC**cgcattcttcggcaatgggaataaatt | 10.39 |  |
| ACHS01000484:29071-29106 | **TTCGC**cgcggttacgcggcca**TTCGC**cagccagtgt | 10.38 |  |
| ACHS01000293:27917-27957 | gtcgcatccacatcgaagac**TTCGC**gcatcgccagcatatt | 10.37 | - XcampvN\_010100016433 (ACHS01000293:27409-27161) hypothetical protein |
| ACHS01000061:13218-13254 | atcgcgcgcccgcgcgcgatttcgtcgcgcacaaact | 10.37 |  |
| ACHS01000293:51407-51449 | tccgccagggcgcgcagcgcttcggcctggtggtggacacctt | 10.37 | - XcampvN\_010100016573 (ACHS01000293:53250-51241) chemotaxis histidine protein kinase COG0643 Chemotaxis protein histidine kinase and related kinases - XcampvN\_010100016568 (ACHS01000293:51142-49019) methyl-accepting chemotaxis protein COG0840 Methyl-accepting chemotaxis protein |
| ACHS01000146:4693-4724 | ttcgacttgatcgccgcgtattcggcgatatt | 10.37 |  |
| ACHS01000126:21434-21469 | **TTCGC**cttgaagcagcacaatcgcgctatgcagact | 10.36 |  |
| ACHS01000069:11654-11709 | ttcgtctttggaaatcaccac**TTCGC**tggcgcccaggcgcagtgcatcagcacgct | 10.35 | - XcampvN\_010100003933 (ACHS01000069:12652-13725) cellulase COG2730 Endoglucanase |
| ACHS01000315:14107-14137 | ttccggcgaacatgcagcaa**TTCGC**tagcct | 10.35 |  |
| ACHS01000381:14212-14246 | ttccccaggccttccatctt**TTCGC**ctttcagctt | 10.35 |  |
| ACHS01000199:9890-9938 | **TTCGC**gctggcggcaatcatctcgcccaccgacccggtggcggtatcgt | 10.35 | - XcampvN\_010100010324 (ACHS01000199:10283-8646) Na :H antiporter COG0025 NhaP-type Na /H and K /H antiporters |
| ACHS01000296:15993-16026 | ttcgtccatgtcggaggttaatcgcaggcataat | 10.34 |  |
| ACHS01000136:14342-14375 | **TTCGC**gcgatgcgccgtatttcggtttccagatt | 10.32 | - metG (ACHS01000136:15067-12989) methionyl-tRNA synthetase COG0143 Methionyl-tRNA synthetase |
| ACHS01000184:17910-17952 | tgcgcccgagtgcgggatta**TTCGC**acattggtctcttatatt | 10.32 | - XcampvN\_010100009842 (ACHS01000184:18348-18521) hypothetical protein - XcampvN\_010100009837 (ACHS01000184:17617-18015) hypothetical protein |
| ACHS01000280:1635-1686 | **TTCGC**caatccaaacgcagtttcgttccgacgctggacttcatcaccacggt | 10.32 | - XcampvN\_010100015293 (ACHS01000280:1334-126) beta-ketoadipyl CoA thiolase COG0183 Acetyl-CoA acetyltransferase - XcampvN\_010100015298 (ACHS01000280:2119-1331) glutaconate CoA transferase subunit B COG2057 Acyl CoA:acetate/3-ketoacid CoA transferase, beta subunit |
| ACHS01000360:3216-3251 | **TTCGC**atcgtgaccaccgac**TTCGC**ctccgacaagt | 10.32 | - XcampvN\_010100020082 (ACHS01000360:2753-2349) hypothetical protein - XcampvN\_010100020077 (ACHS01000360:2346-1672) DNA-3-methyladenine glycosylase COG0122 3-methyladenine DNA glycosylase/8-oxoguanine DNA glycosylase - XcampvN\_010100020087 (ACHS01000360:3999-2818) hypothetical protein |
| ACHS01000471:35874-35905 | tacgcggccccgcccaggtgttcgtacagaat | 10.32 |  |
| ACHS01000330:3877-3907 | tgcgccagcgtgccggtgtattcggcatacg | 10.31 |  |
| ACHS01000408:19357-19386 | ttcacctgcgcgcgtgctg**TTCGC**cagctt | 10.29 | - XcampvN\_010100021677 (ACHS01000408:19322-20602) metabolite transport protein COG0477 Permeases of the major facilitator superfamily |
| ACHS01000033:3951-3980 | **TTCGC**tgctggcgcagcggttgcgccagat | 10.29 | - XcampvN\_010100001659 (ACHS01000033:3478-6606) acriflavin resistance protein COG0841 Cation/multidrug efflux pump |
| ACHS01000165:51161-51192 | atcgcccgcgcatgaagctcatcgcccacgtt | 10.28 | - XcampvN\_010100009097 (ACHS01000165:51124-51879) hypothetical protein |
| ACHS01000426:2178-2208 | atcgccgccgaggccggcactgcgccatcca | 10.27 |  |
| ACHS01000204:26667-26723 | **TTCGC**gcacccgcgcaggatc**TTCGC**gcccggccagttccagcggcagcgcgatgtt | 10.27 | - XcampvN\_010100010989 (ACHS01000204:26311-25658) acyl-CoA thioesterase I COG2755 Lysophospholipase L1 and related esterases |
| ACHS01000208:12240-12268 | **TTCGC**cggccgggtagac**TTCGC**tcaaca | 10.24 |  |
| ACHS01000218:9194-9227 | **TTCGC**aaacagcgaaggatg**TTCGC**gacgtagct | 10.23 |  |
| ACHS01000416:10837-10868 | atcgcttgcgcgaccagcgttttcggcaacct | 10.23 | - XcampvN\_010100022039 (ACHS01000416:10341-10099) hypothetical protein COG1671 Uncharacterized protein conserved in bacteria - XcampvN\_010100022034 (ACHS01000416:10028-9480) hypothetical protein COG4681 Uncharacterized protein conserved in bacteria |
| ACHS01000011:3721-3774 | atcgtcattggacgaagctgttcggttctggacaggagcactcggctttacgct | 10.23 | - XcampvN\_010100000310 (ACHS01000011:3914-3363) hypothetical protein COG0346 Lactoylglutathione lyase and related lyases |
| ACHS01000146:9232-9262 | **TTCGC**cggcgggcgcctggtgtcgccaaaag | 10.22 | - XcampvN\_010100007905 (ACHS01000146:10087-9053) LysR family transcriptional regulator COG0583 Transcriptional regulator |
| ACHS01000208:54198-54228 | tgcgccttgcccagcgcggcttcggcatctt | 10.22 |  |
| ACHS01000020:19916-19991 | **TTCGC**cactccaggcagaaatcgcctgatccggatcattgccgtagcgcttacgcagatccggctgcagatacagt | 10.21 |  |
| ACHS01000326:17311-17341 | **TTCGC**cggcgccgccaacacatcgtggcatt | 10.21 | - XcampvN\_010100018275 (ACHS01000326:18892-16232) hypothetical protein COG2114 Adenylate cyclase, family 3 (some proteins contain HAMP domain) |
| ACHS01000243:14864-14902 | atcgccgacacccgcatggt**TTCGC**cggggcggtacaga | 10.19 |  |
| ACHS01000296:39449-39495 | atcgctgtcggctgcagggtttcggccaggtgcaggacctccatcgt | 10.19 |  |
| ACHS01000467:912-969 | **TTCGC**atgaaaatcaaacgcttcgttgccccggacatgcgcaccgctttccgcatggt | 10.18 | - XcampvN\_010100024370 (ACHS01000467:917-2602) flagellar biosynthesis regulator FlhF COG1419 Flagellar GTP-binding protein |
| ACHS01000381:10632-10684 | **TTCGC**ctgcgcatccgctggtttccatgcccatccctgccaagacgccagcct | 10.18 |  |
| ACHS01000211:10339-10376 | tgcgcccacggcccgatggt**TTCGC**cactgatcaccat | 10.18 |  |
| ACHS01000223:2649-2699 | ttcttcctgcagtgcagcac**TTCGC**accgcgctacaccggccgggcacgtt | 10.17 | - XcampvN\_010100012785 (ACHS01000223:2416-2885) outer protein X |
| ACHS01000069:34250-34284 | ttcgtcgccatccccgccgtg**TTCGC**gttcaactt | 10.17 | - XcampvN\_010100004038 (ACHS01000069:33683-33270) biopolymer transport ExbD protein COG0848 Biopolymer transport protein - XcampvN\_010100004048 (ACHS01000069:34917-34156) biopolymer transport ExbB protein COG0811 Biopolymer transport proteins - XcampvN\_010100004043 (ACHS01000069:34109-33687) biopolymer transport ExbD1 protein COG0848 Biopolymer transport protein |
| ACHS01000091:1953-1983 | gtcgccgcgctggccgaatt**TTCGC**ccgatt | 10.17 | - XcampvN\_010100004638 (ACHS01000091:1152-2247) DNA polymerase III subunits gamma and tau COG2812 DNA polymerase III, gamma/tau subunits |
| ACHS01000409:6964-7016 | **TTCGC**gttgcttggtcagctc**TTCGC**cgacgcgccgcagcaacgcgccacgct | 10.17 |  |
| ACHS01000042:8985-9028 | **TTCGC**gcgcgaattcgtcaactcgccgcgccgcgaattcaatat | 10.15 | - XcampvN\_010100001979 (ACHS01000042:9962-10357) amidase |
| ACHS01000219:39725-39755 | tctgccagccgctgcagcgcttcggcatatt | 10.15 |  |
| ACHS01000326:22277-22307 | atcgcctgctcattggcggcttcgtcatgat | 10.14 |  |
| ACHS01000220:13842-13871 | ttcggcctcggccaaggtcatcgccacgat | 10.14 |  |
| ACHS01000131:74760-74800 | ttcggcgtctactcgaacta**TTCGC**tggtggattccaacct | 10.14 | - XcampvN\_010100006736 (ACHS01000131:74196-72985) arabinogalactan endo-1,4-beta-galactosidase COG3867 Arabinogalactan endo-1,4-beta-galactosidase - XcampvN\_010100006741 (ACHS01000131:77080-74387) TonB-dependent receptor COG1629 Outer membrane receptor proteins, mostly Fe transport |
| ACHS01000465:3550-3581 | ttcgtcggcgacgaacacgggttccgcaaact | 10.12 | - XcampvN\_010100024340 (ACHS01000465:3214-6135) Putative signal protein with GGDEF domain COG3292 Predicted periplasmic ligand-binding sensor domain |
| ACHS01000416:11263-11293 | **TTCGC**ccgagcgtctgcagttccgctattgg | 10.12 | - XcampvN\_010100022054 (ACHS01000416:11686-11790) hypothetical protein - XcampvN\_010100022049 (ACHS01000416:10991-11689) glutathione S-transferase COG0625 Glutathione S-transferase |
| ACHS01000113:9473-9503 | ttctcagccgtgcctgccag**TTCGC**caatga | 10.11 | - XcampvN\_010100005152 (ACHS01000113:10233-11669) hypothetical protein - XcampvN\_010100005147 (ACHS01000113:9533-10222) ABC transporter ATP-binding protein COG1131 ABC-type multidrug transport system, ATPase component |
| ACHS01000208:48408-48438 | **TTCGC**cccaccaccgcgcgctttgcgatcat | 10.11 | - nusB (ACHS01000208:47999-47529) transcription antitermination protein NusB COG0781 Transcription termination factor - ribH (ACHS01000208:48460-47996) 6,7-dimethyl-8-ribityllumazine synthase COG0054 Riboflavin synthase beta-chain |
| ACHS01000230:1435-1466 | ttcgtgcaacgcttccagcacttcgttggagt | 10.1 |  |
| ACHS01000061:44597-44625 | atcgcaccgcgccagttg**TTCGC**cagcct | 10.1 | - XcampvN\_010100003223 (ACHS01000061:42359-44641) TonB-dependent receptor COG1629 Outer membrane receptor proteins, mostly Fe transport - XcampvN\_010100003228 (ACHS01000061:44735-46318) flavin monoamine oxidase-related protein COG1231 Monoamine oxidase |
| ACHS01000208:43925-43986 | ttcgtgtgggtgaacaccatttccctcgctgcctccagccacgatttggctctttgcaaaat | 10.09 | - XcampvN\_010100011464 (ACHS01000208:43870-42611) hypothetical protein |
| ACHS01000164:1996-2055 | **TTCGC**cgtgggcgatcaa**TTCGC**ggcattccaccgcgaagatgcccatctgccctacctt | 10.07 | - XcampvN\_010100008862 (ACHS01000164:2973-3623) putative SCO1/SenC family protein COG1999 Uncharacterized protein SCO1/SenC/PrrC, involved in biogenesis of respiratory and photosynthetic systems |
| ACHS01000144:843-888 | ttcggatgacgagaaagtatttctcaccaaggtttactggtattgt | 10.06 | - XcampvN\_010100007668 (ACHS01000144:464-2608) colicin V processing peptidase cysteine peptidase MEROPS family C39 COG2274 ABC-type bacteriocin/lantibiotic exporters, contain an N-terminal double-glycine peptidase domain |
| ACHS01000183:10300-10338 | **TTCGC**gctcggcgcgcacggcttcggtgccggccatctt | 10.05 |  |
| ACHS01000351:22644-22694 | atcgccgctgcatccagtccctcgcgcgggctggtgccgaccacgcacagt | 10.03 | - XcampvN\_010100019865 (ACHS01000351:23580-24554) putative endo-1,4-beta-mannosidase COG2730 Endoglucanase |
| ACHS01000159:1063-1093 | tgcgtccgcgcaccaacctgttcggtgcggt | 10.03 | - asnC (ACHS01000159:1466-72) asparaginyl-tRNA synthetase COG0017 Aspartyl/asparaginyl-tRNA synthetases |
| ACHS01000302:14403-14433 | ttggccttgcgcgccttggc**TTCGC**catagg | 10.02 |  |
| ACHS01000442:20255-20297 | ttccctggcaccgcgacagc**TTCGC**cagccagcgcgacagcgt | 10.02 | - XcampvN\_010100023182 (ACHS01000442:20532-20996) low molecular weight phosphotyrosine protein phosphatase COG0394 Protein-tyrosine-phosphatase - XcampvN\_010100023187 (ACHS01000442:21020-22438) hypothetical protein COG0526 Thiol-disulfide isomerase and thioredoxins - XcampvN\_010100023177 (ACHS01000442:19756-20535) 3-deoxy-manno-octulosonate cytidylyltransferase COG1212 CMP-2-keto-3-deoxyoctulosonic acid synthetase |
| ACHS01000060:7134-7164 | tgcgctatgccacgcacatg**TTCGC**caacga | 10.01 | - XcampvN\_010100002988 (ACHS01000060:7678-6758) lipid A biosynthesis lauroyl acyltransferase COG1560 Lauroyl/myristoyl acyltransferase |
