# Supplementary figures and images for: Genome Sequencing of Xanthomonas vasicola Pathovar vasculorum Reveals Variation in Plasmids and Genes Encoding Lipopolysaccharide Synthesis, Type-IV Pilus and Type-III Secretion Effectors
Source: Pathogens. 2014 Mar 18;3(1):211–37. doi: 10.3390/pathogens3010211 (PMC4235730; doi:10.3390/pathogens3010211)

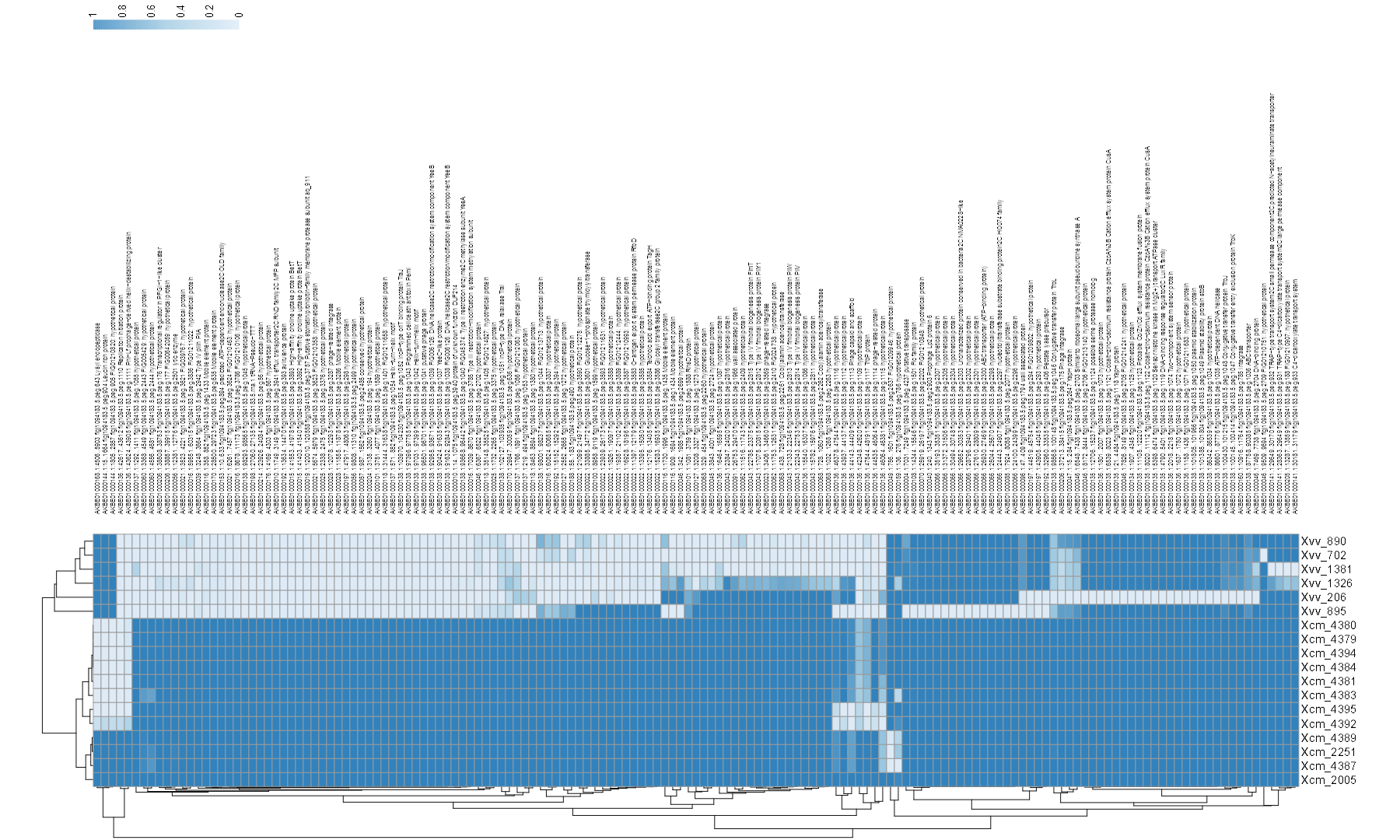

Supplement: Supplementary File 1 — Supplementary Materials (TAR, 5271 KB) [file pathogens-03-00211-s001.tar › supplementary/images/gene-content-versus-Xcm2005.png]

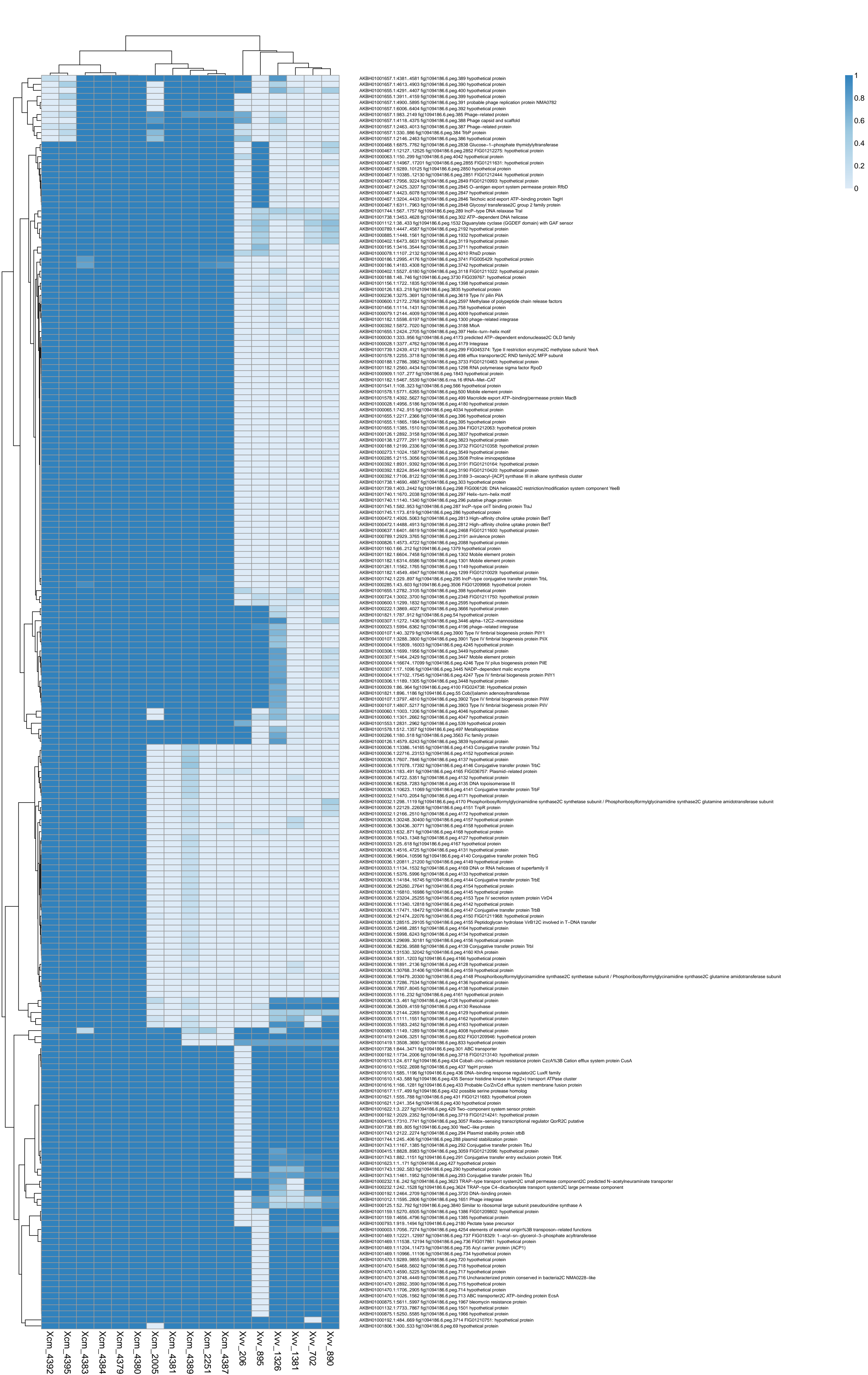

Supplement: Supplementary File 1 — Supplementary Materials (TAR, 5271 KB) [file pathogens-03-00211-s001.tar › supplementary/images/gene-content-versus-Xcm4384.pdf]

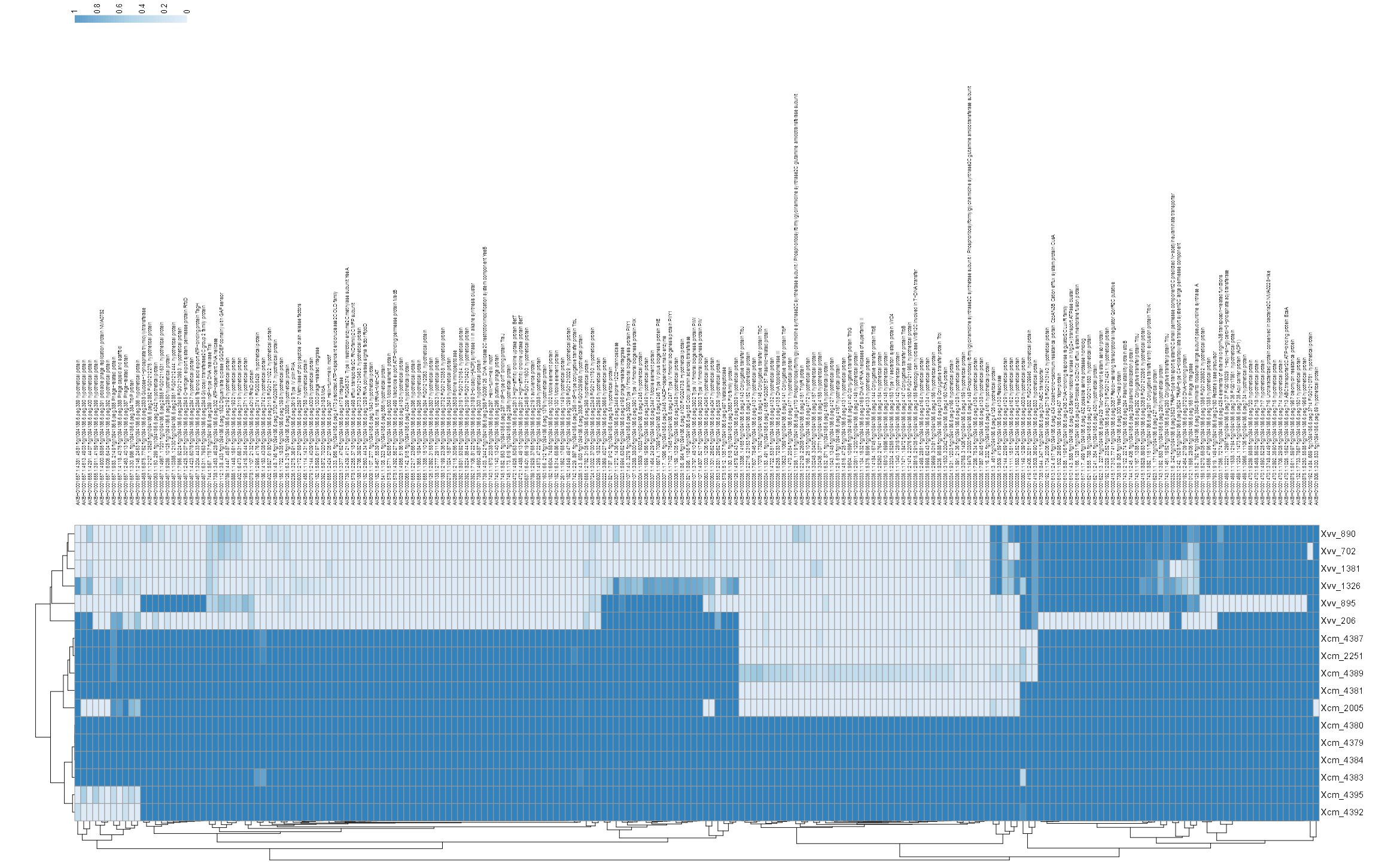

Supplement: Supplementary File 1 — Supplementary Materials (TAR, 5271 KB) [file pathogens-03-00211-s001.tar › supplementary/images/gene-content-versus-Xcm4384.png]

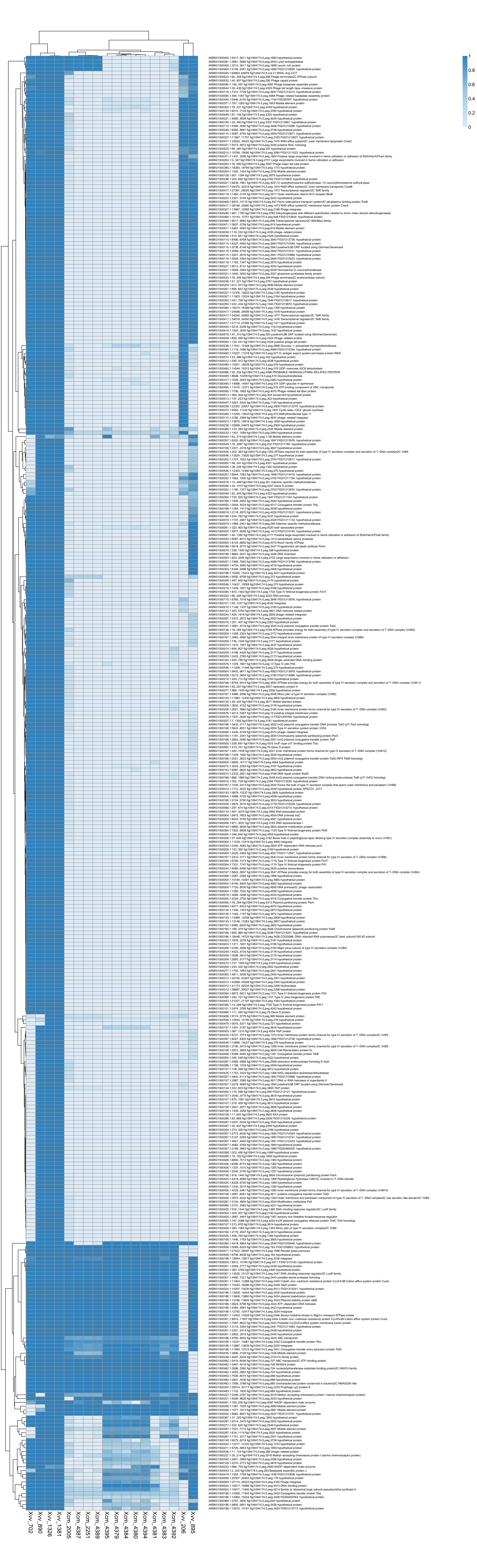

Supplement: Supplementary File 1 — Supplementary Materials (TAR, 5271 KB) [file pathogens-03-00211-s001.tar › supplementary/images/gene-content-versus-Xvv1326.pdf]

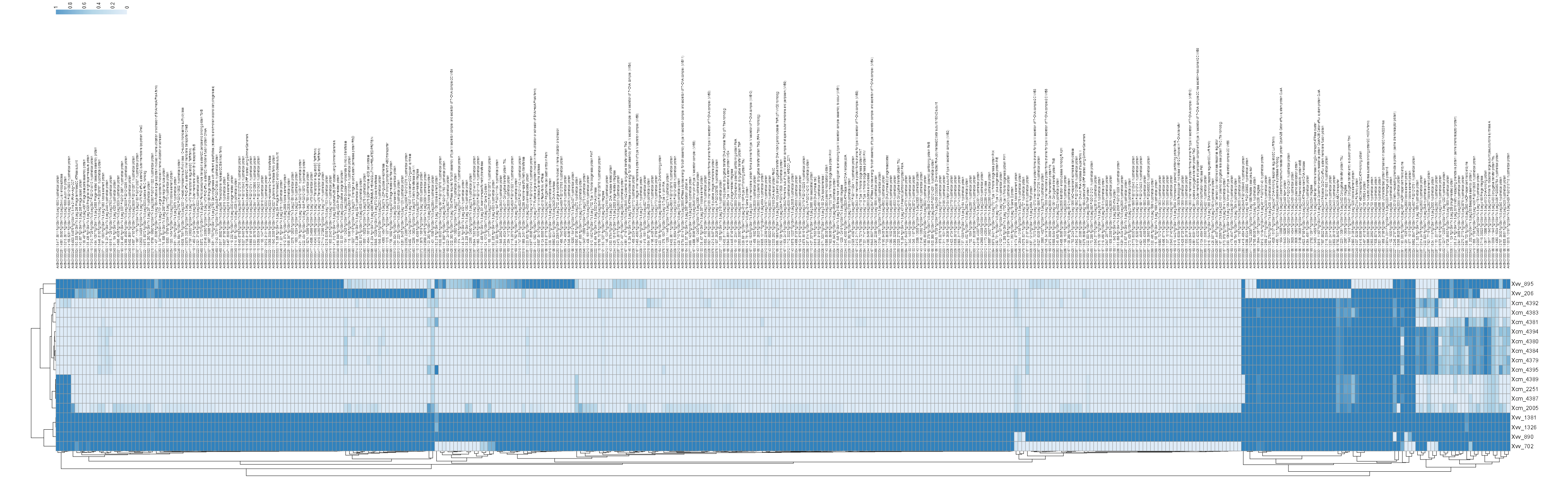

Supplement: Supplementary File 1 — Supplementary Materials (TAR, 5271 KB) [file pathogens-03-00211-s001.tar › supplementary/images/gene-content-versus-Xvv1326.png]

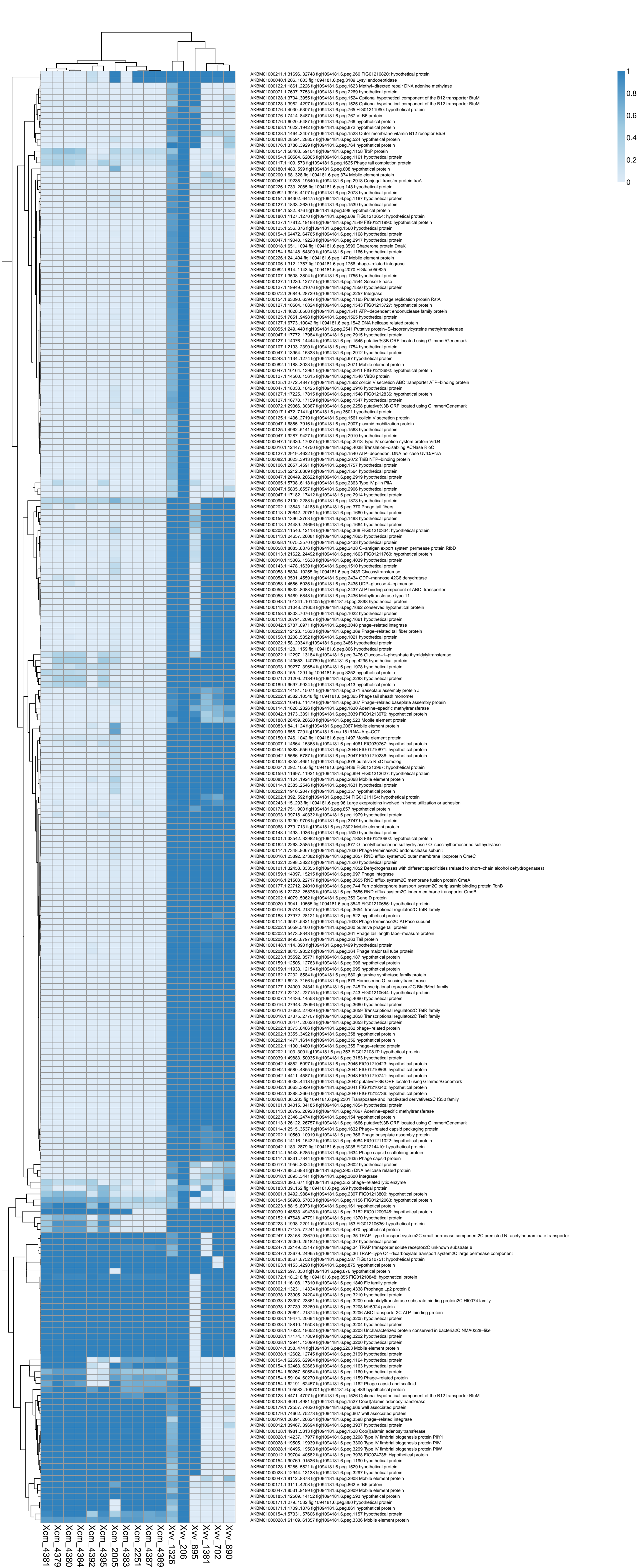

Supplement: Supplementary File 1 — Supplementary Materials (TAR, 5271 KB) [file pathogens-03-00211-s001.tar › supplementary/images/gene-content-versus-Xvv206.pdf]

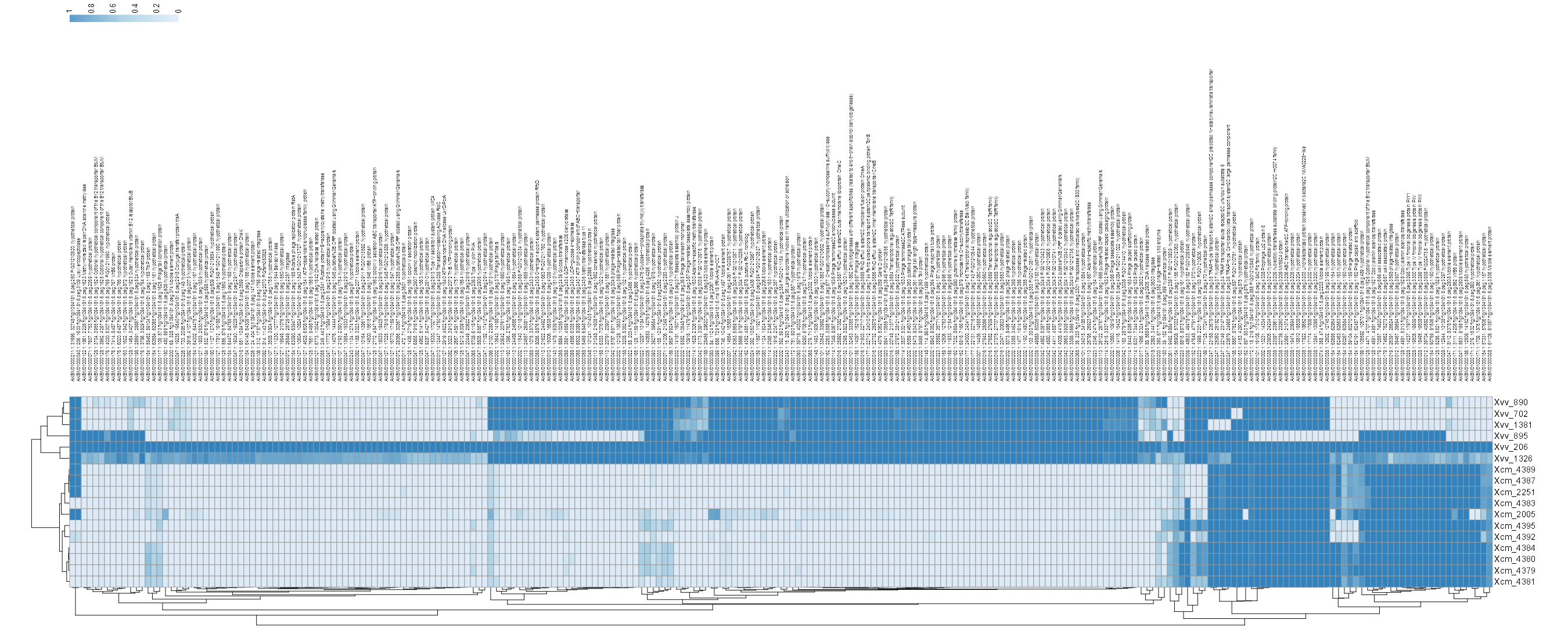

Supplement: Supplementary File 1 — Supplementary Materials (TAR, 5271 KB) [file pathogens-03-00211-s001.tar › supplementary/images/gene-content-versus-Xvv206.png]

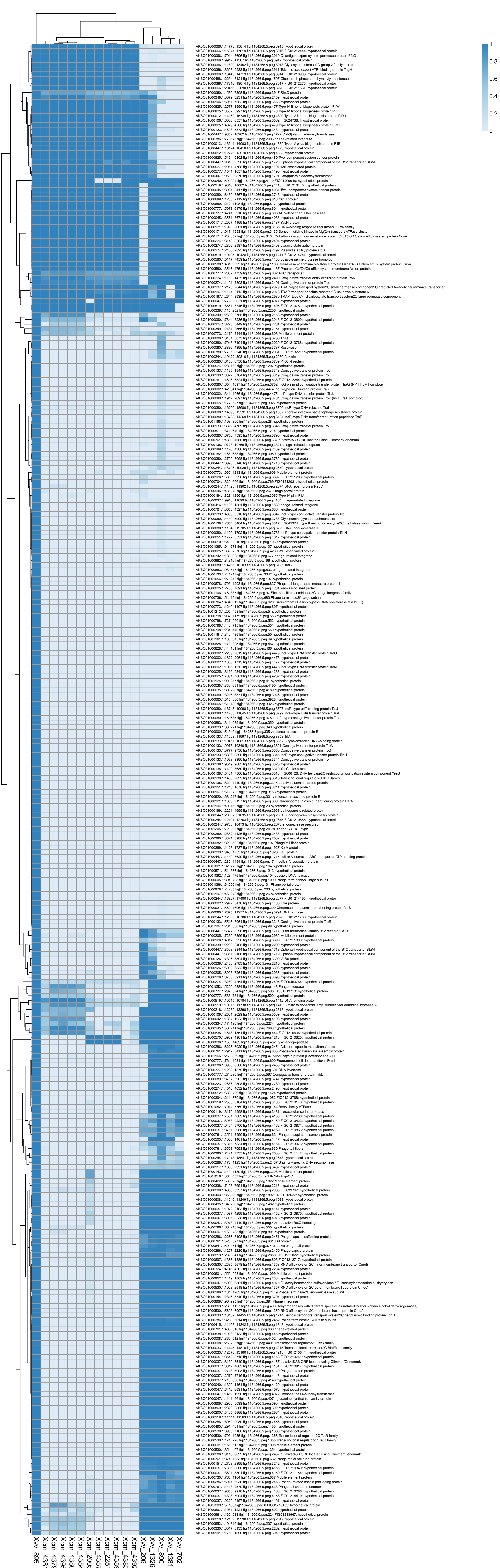

Supplement: Supplementary File 1 — Supplementary Materials (TAR, 5271 KB) [file pathogens-03-00211-s001.tar › supplementary/images/gene-content-versus-Xvv895.pdf]

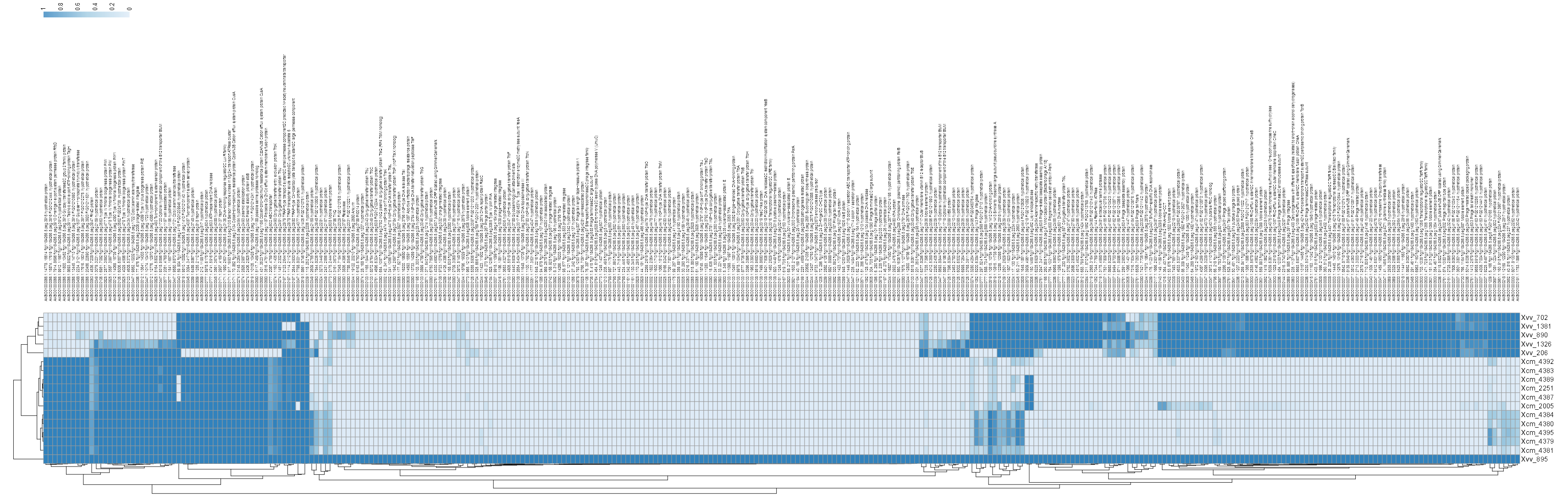

Supplement: Supplementary File 1 — Supplementary Materials (TAR, 5271 KB) [file pathogens-03-00211-s001.tar › supplementary/images/gene-content-versus-Xvv895.png]

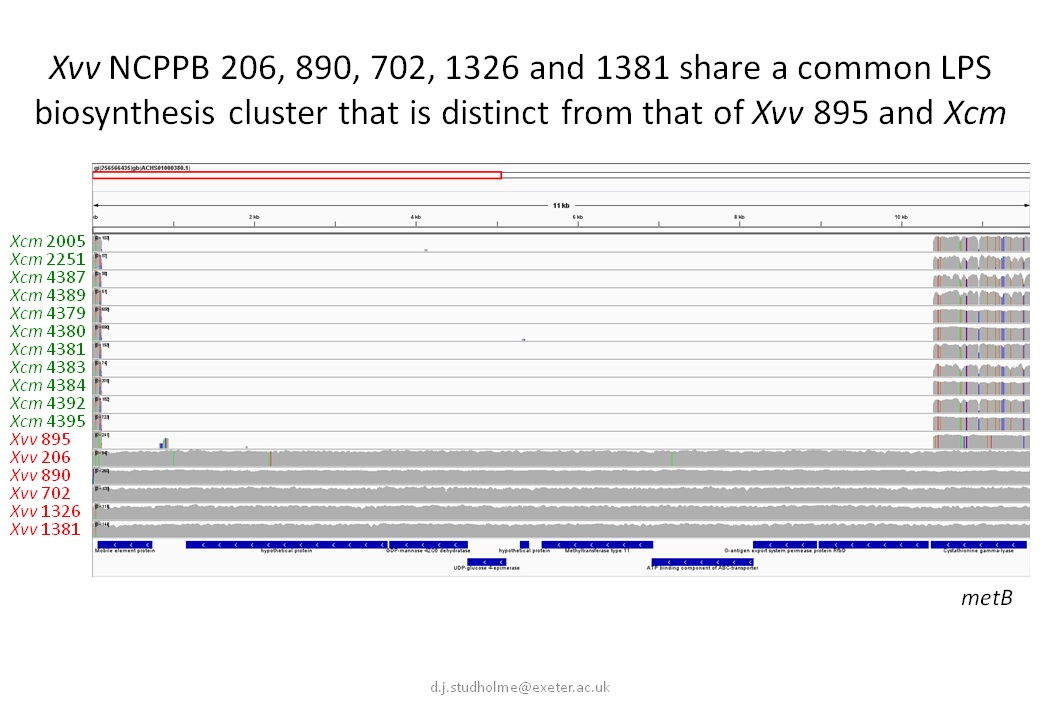

Supplement: Supplementary File 1 — Supplementary Materials (TAR, 5271 KB) [file pathogens-03-00211-s001.tar › supplementary/images/S10.png]

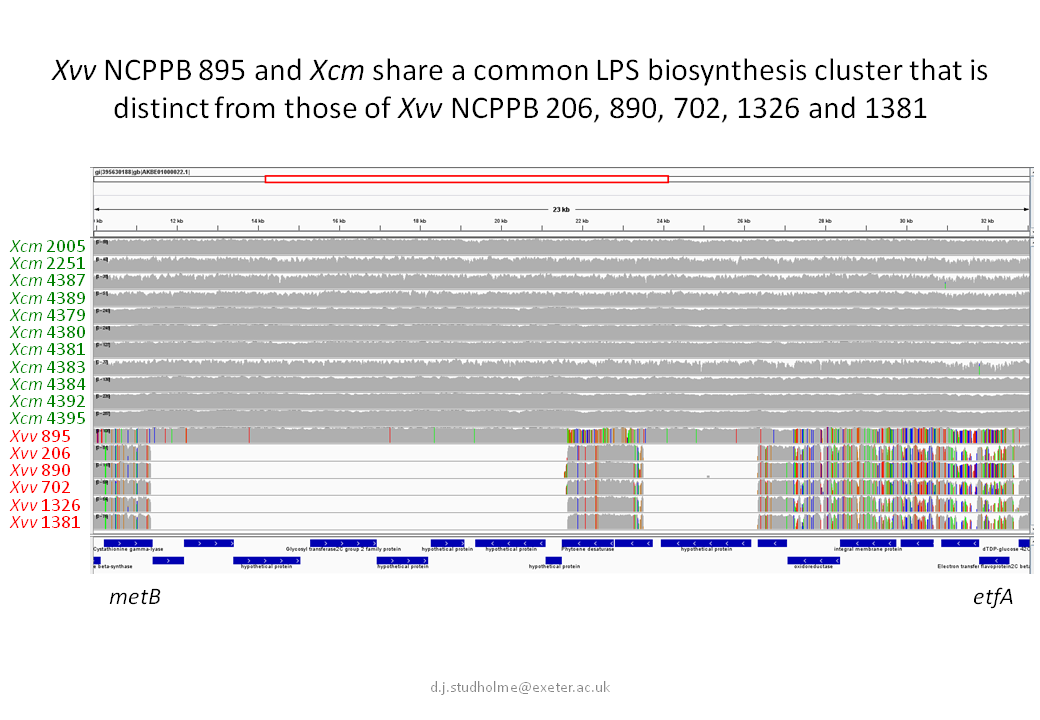

Supplement: Supplementary File 1 — Supplementary Materials (TAR, 5271 KB) [file pathogens-03-00211-s001.tar › supplementary/images/S11.png]

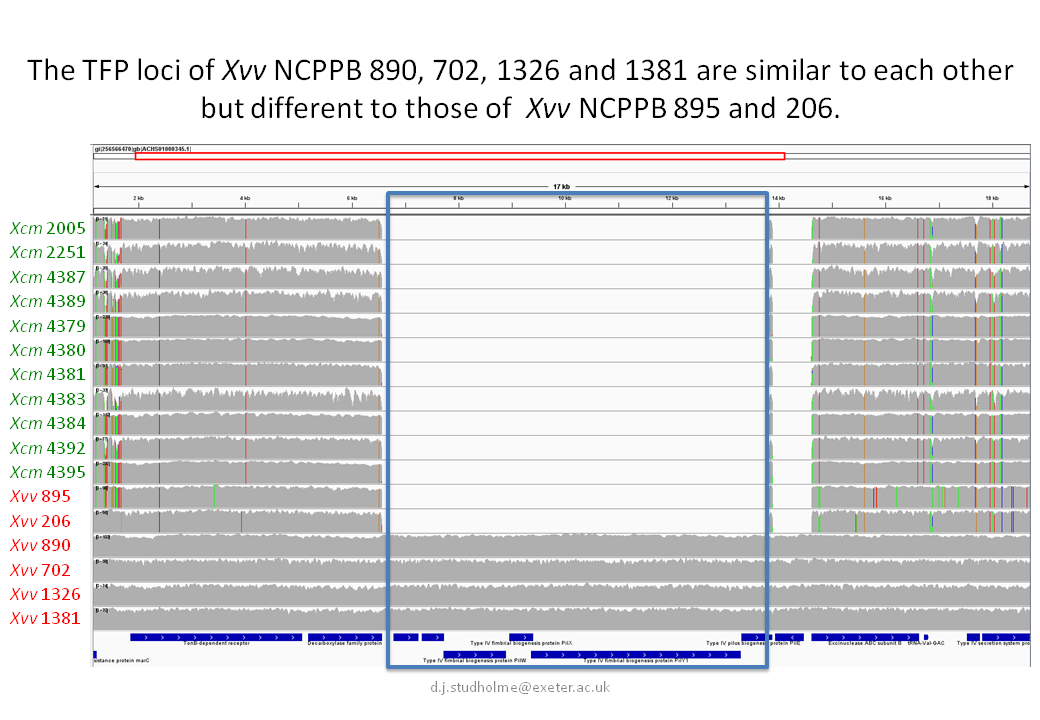

Supplement: Supplementary File 1 — Supplementary Materials (TAR, 5271 KB) [file pathogens-03-00211-s001.tar › supplementary/images/S12.png]

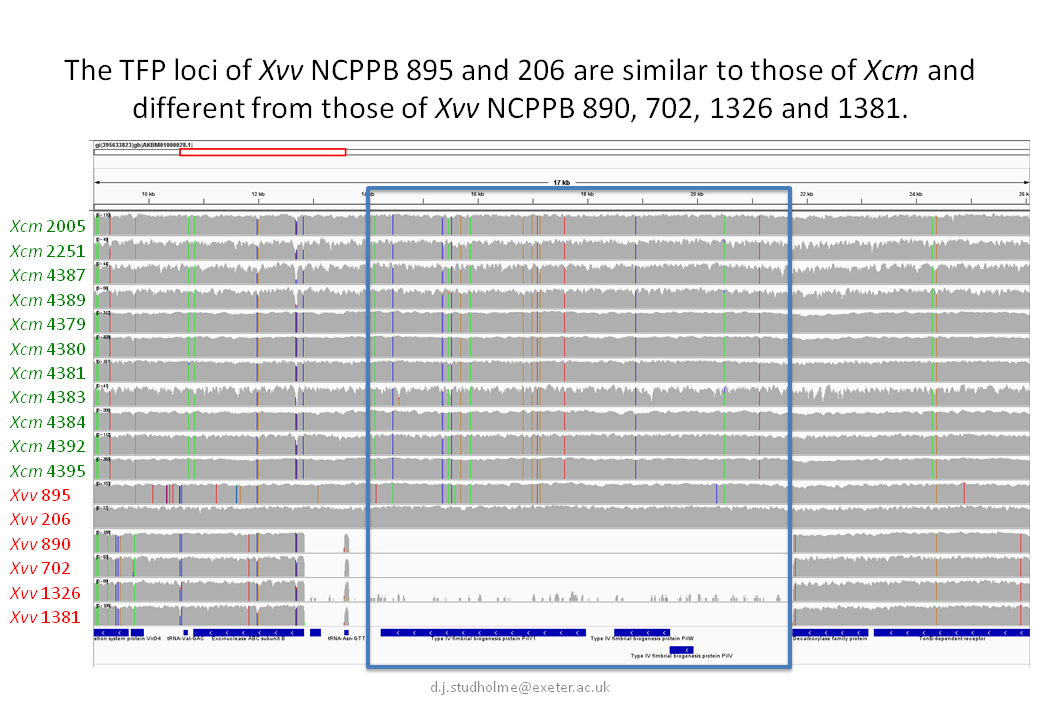

Supplement: Supplementary File 1 — Supplementary Materials (TAR, 5271 KB) [file pathogens-03-00211-s001.tar › supplementary/images/S13.png]

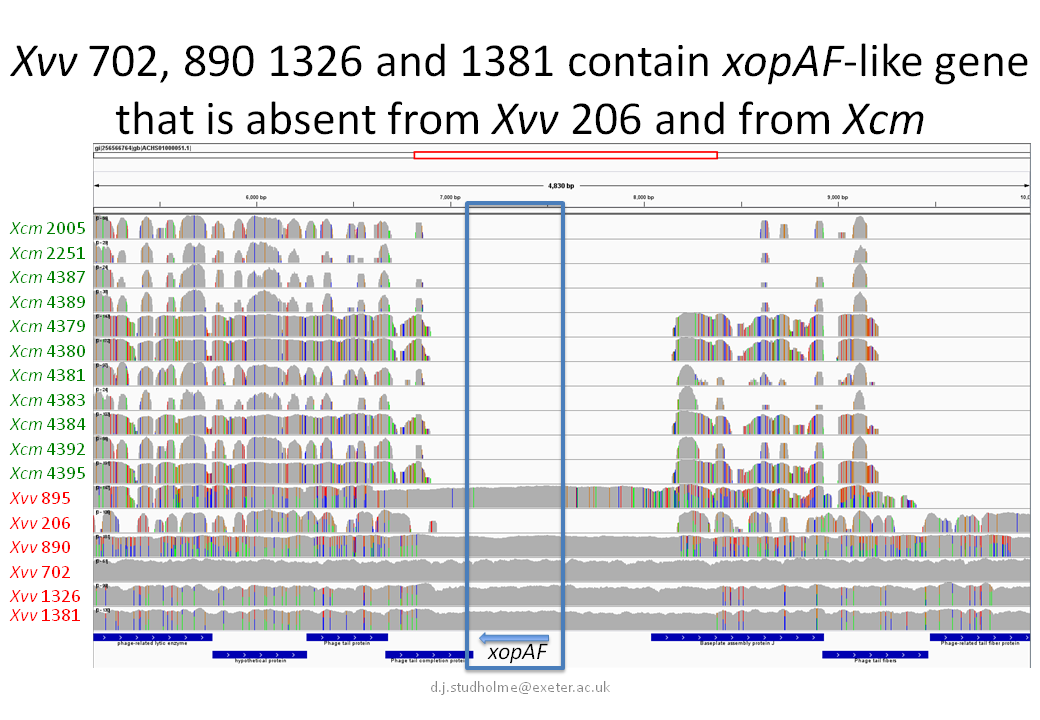

Supplement: Supplementary File 1 — Supplementary Materials (TAR, 5271 KB) [file pathogens-03-00211-s001.tar › supplementary/images/S14.png]

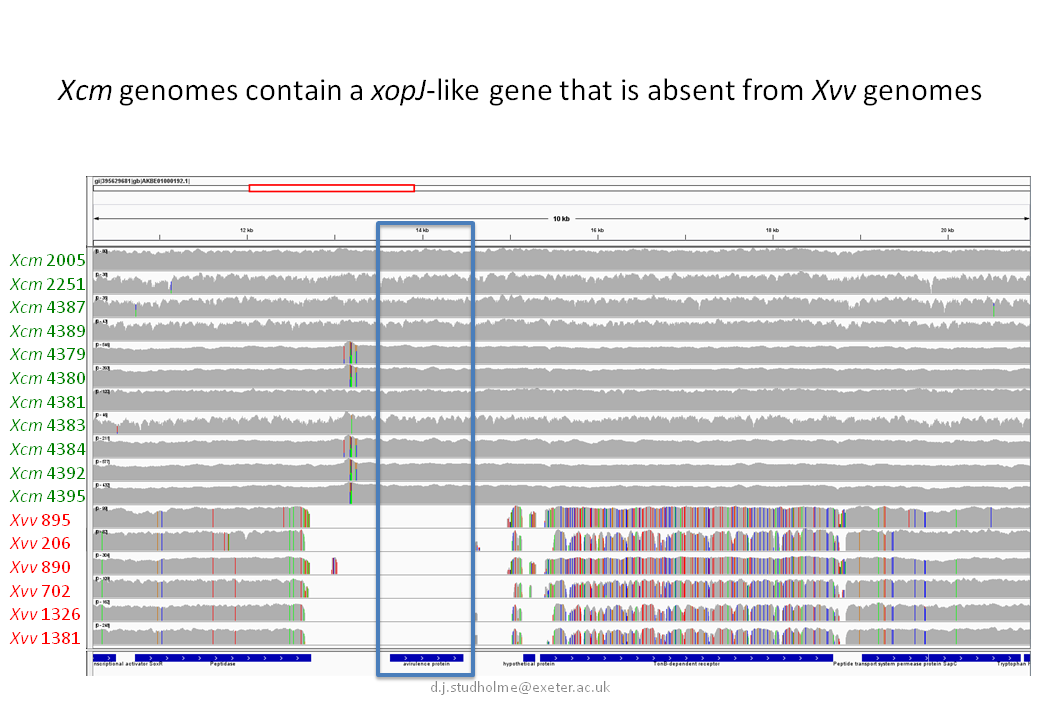

Supplement: Supplementary File 1 — Supplementary Materials (TAR, 5271 KB) [file pathogens-03-00211-s001.tar › supplementary/images/S15.png]

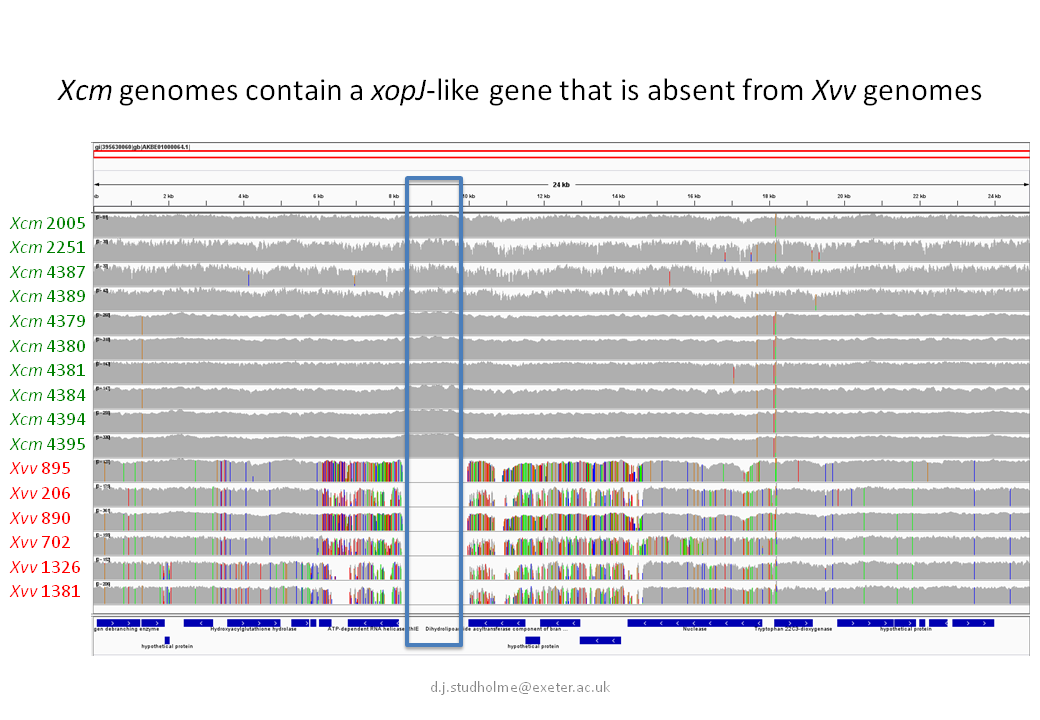

Supplement: Supplementary File 1 — Supplementary Materials (TAR, 5271 KB) [file pathogens-03-00211-s001.tar › supplementary/images/S16.png]

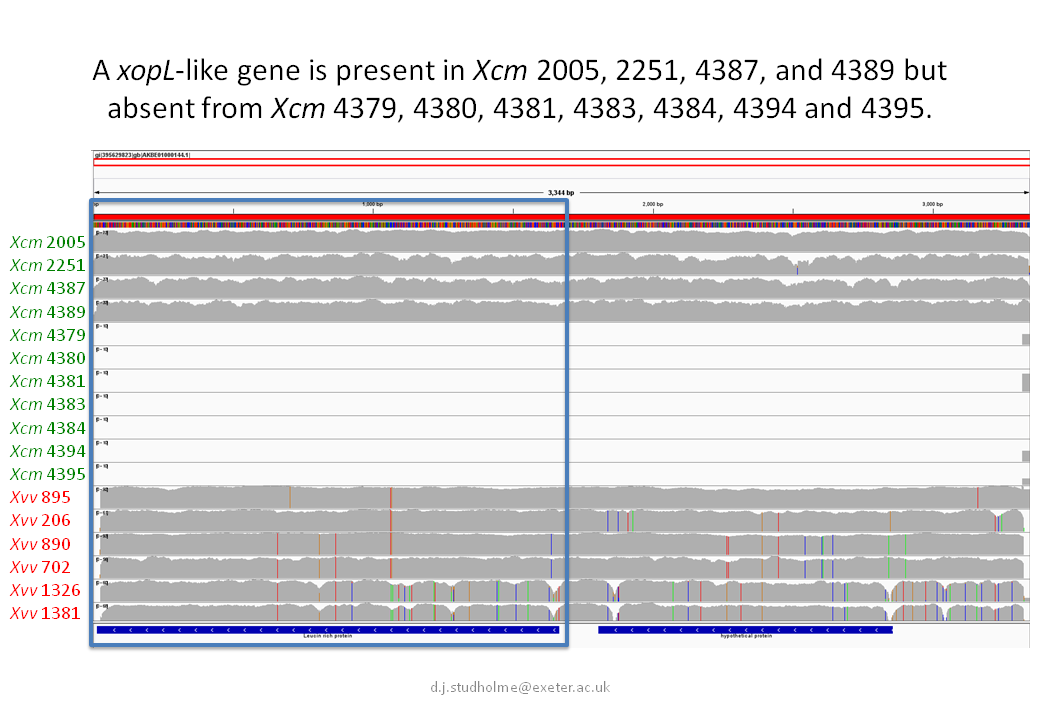

Supplement: Supplementary File 1 — Supplementary Materials (TAR, 5271 KB) [file pathogens-03-00211-s001.tar › supplementary/images/S17.png]

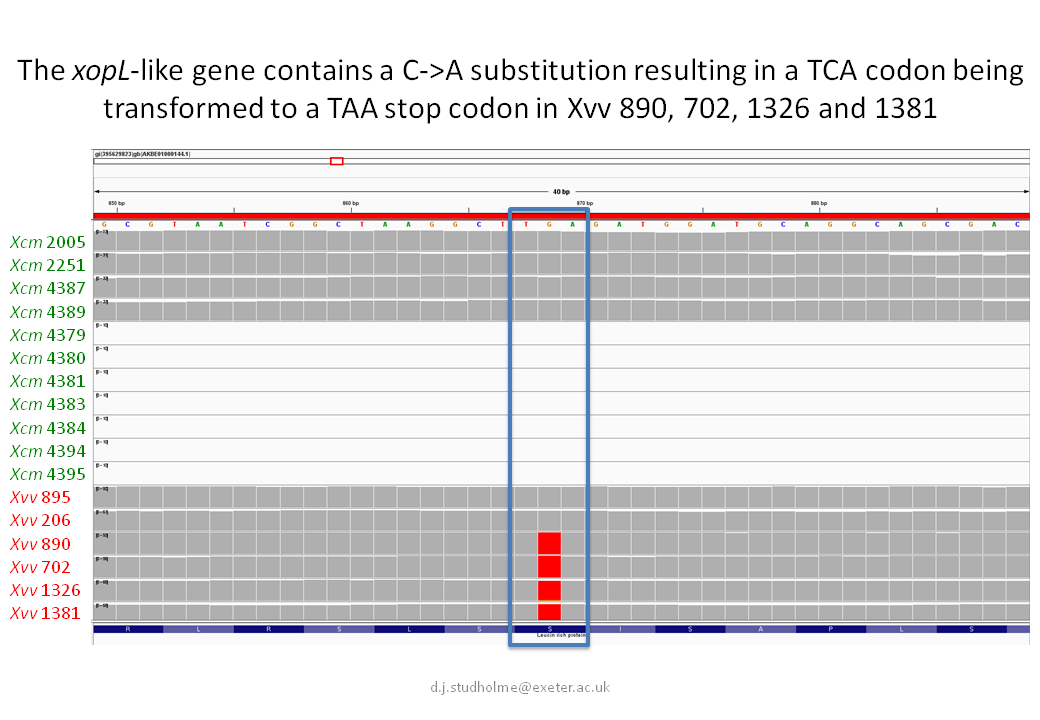

Supplement: Supplementary File 1 — Supplementary Materials (TAR, 5271 KB) [file pathogens-03-00211-s001.tar › supplementary/images/S18.png]

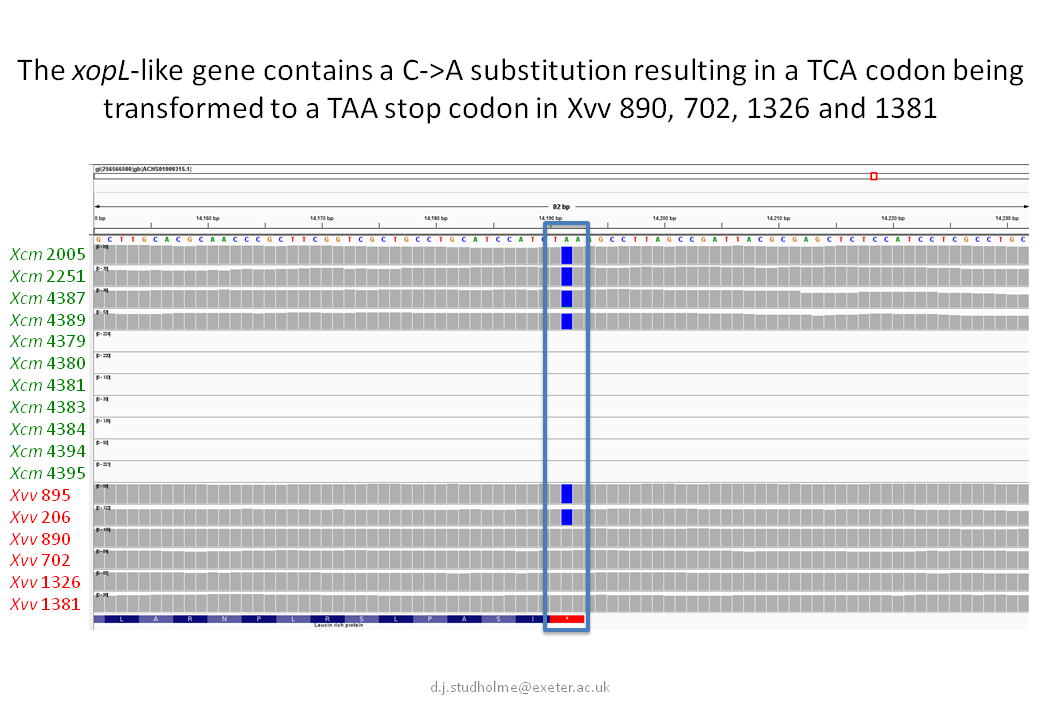

Supplement: Supplementary File 1 — Supplementary Materials (TAR, 5271 KB) [file pathogens-03-00211-s001.tar › supplementary/images/S19.png]

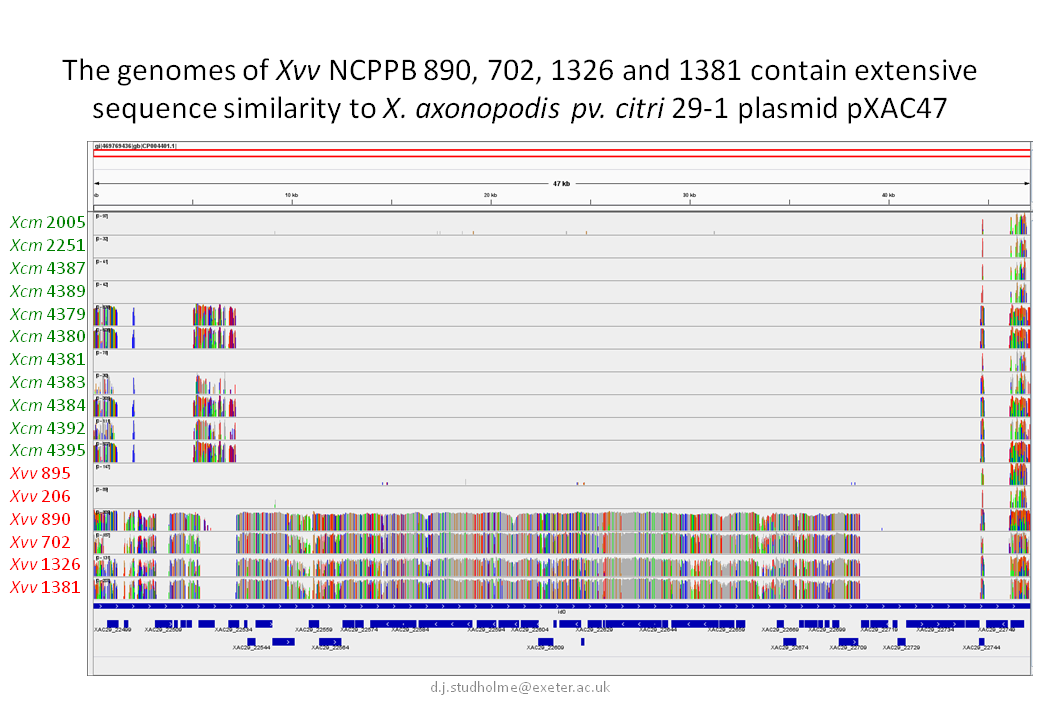

Supplement: Supplementary File 1 — Supplementary Materials (TAR, 5271 KB) [file pathogens-03-00211-s001.tar › supplementary/images/S6.png]

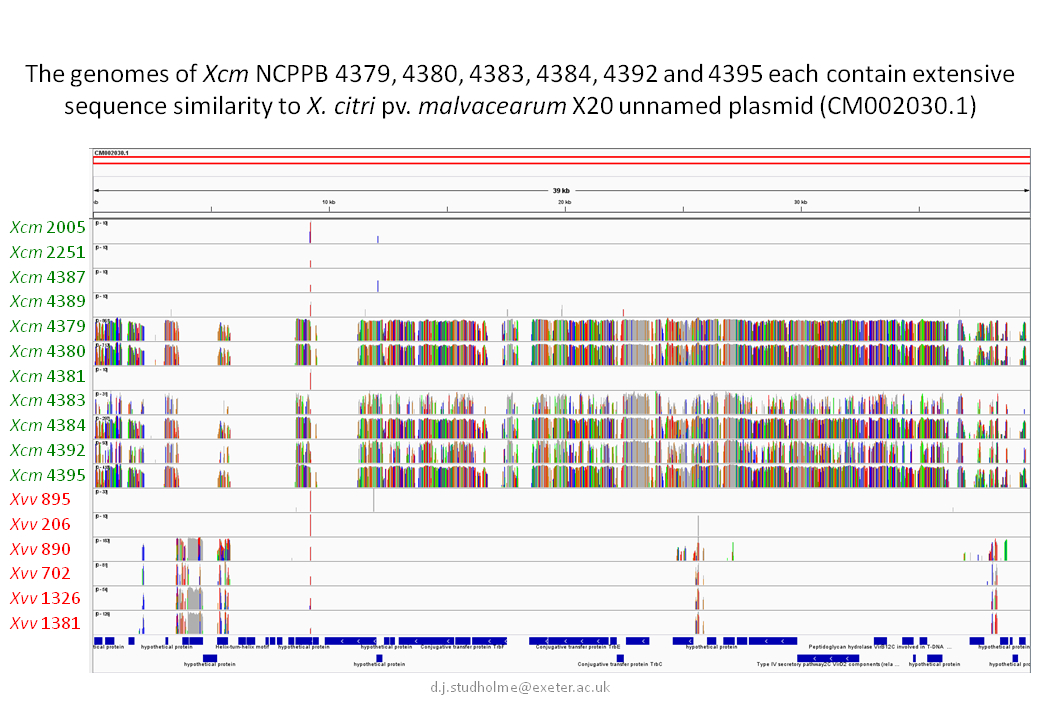

Supplement: Supplementary File 1 — Supplementary Materials (TAR, 5271 KB) [file pathogens-03-00211-s001.tar › supplementary/images/S7.png]

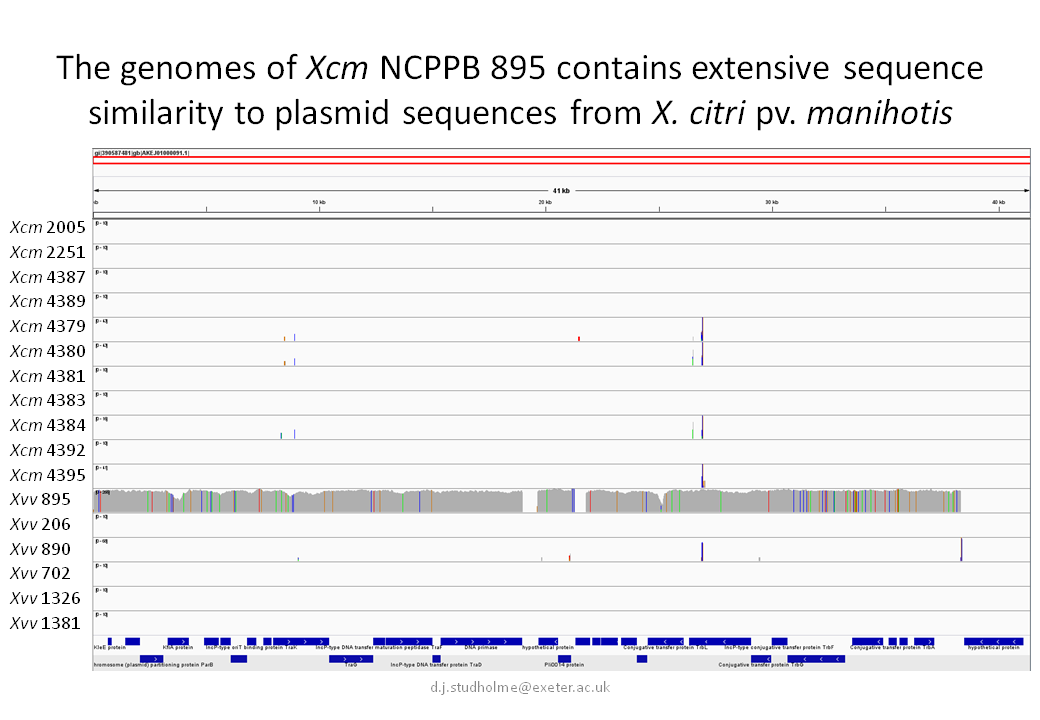

Supplement: Supplementary File 1 — Supplementary Materials (TAR, 5271 KB) [file pathogens-03-00211-s001.tar › supplementary/images/S8.png]

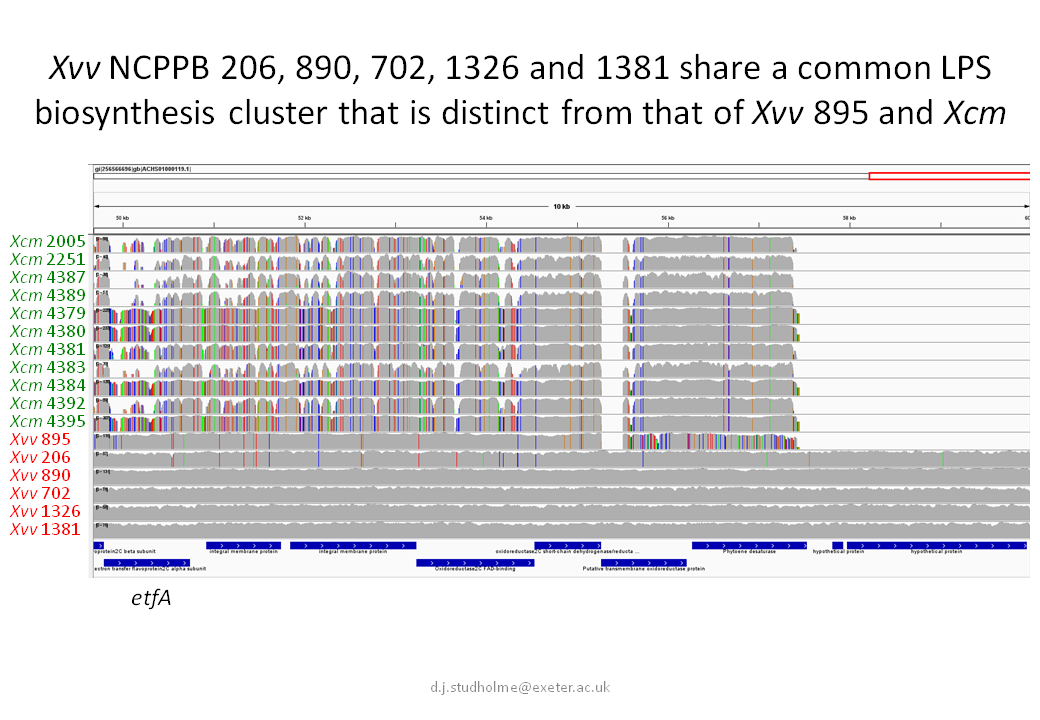

Supplement: Supplementary File 1 — Supplementary Materials (TAR, 5271 KB) [file pathogens-03-00211-s001.tar › supplementary/images/S9.png]

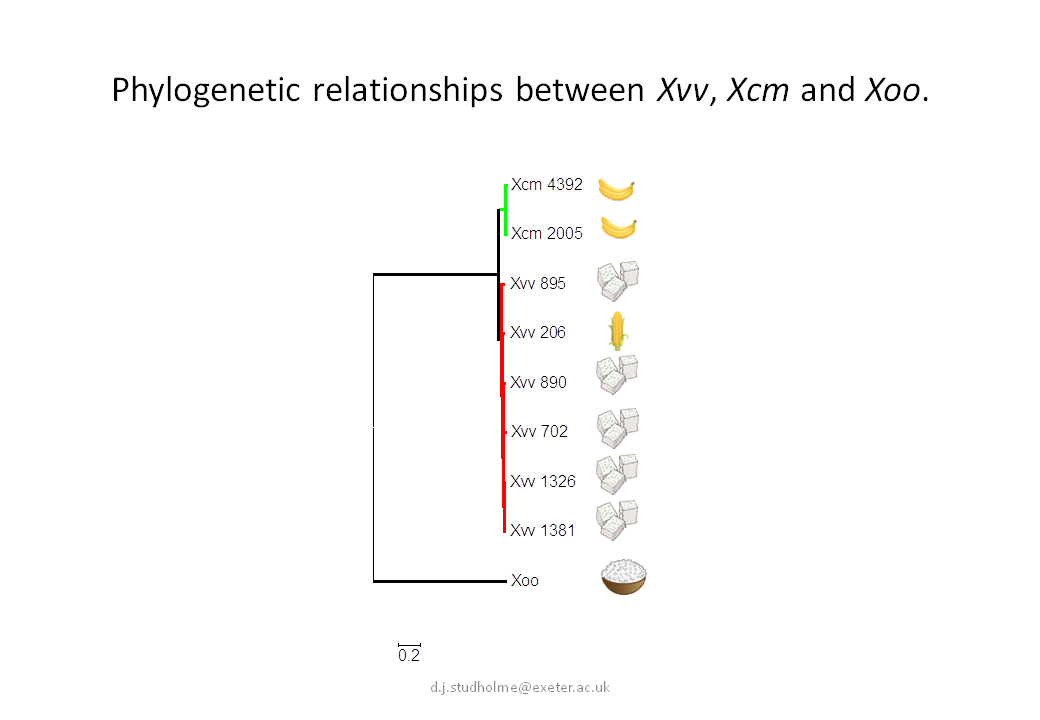

Supplement: Supplementary File 1 — Supplementary Materials (TAR, 5271 KB) [file pathogens-03-00211-s001.tar › supplementary/images/Slide1.PNG]
